# Therapeutic potential of *Leonotis leonurus* and *Mentha longifolia* in depression: insights from network pharmacology and molecular dynamics stimulation-based study

**DOI:** 10.1007/s40203-026-00689-2

**Published:** 2026-07-13

**Authors:** Caroline Mooki, Oladunni M. Ayodele, Saheed Sabiu, Adeyemi O. Aremu, Makhotso Lekhooa

**Affiliations:** 1 Preclinical Drug Development Platform, Faculty of Health Sciences, North-West University, Private Bag X6001, Potchefstroom, 2521 South Africa; 2 Centre of Excellence in Indigenous Knowledge Systems, Faculty of Natural and Agricultural Sciences, North-West University, Private Bag X2046, Mmabatho, 2745 South Africa; 3 South African Research Chairs Initiative in Indigenous Knowledge-Driven Medicinal Plants Utilisation and Conservation Strategies for Human, Animal, Crop Health (IK-Medplants4HAC), Faculty of Natural and Agricultural Sciences, North-West University, Private Bag X2046, Mmabatho, 2745 South Africa; 4 Department of Biotechnology and Food Science, Faculty of Applied Sciences, Durban University of Technology, P.O. Box 1334, Durban, 4000 South Africa

**Keywords:** Biodiversity, Computational analysis, Neurochemical, Network pharmacology, Wild dagga

## Abstract

Medicinal plants offer promising multi-target therapeutic agents due to their diverse bioactive compounds. This study explored LC–MS profiling, network pharmacology, molecular docking, and molecular dynamics simulation to investigate the potential antidepressant-related mechanisms of *Leonotis leonurus* and *Mentha longifolia*. A total of 20 and 15 compounds were identified in *L. leonurus* and *M*. *longifolia*, respectively. Target prediction and overlap analysis revealed 16 and 22 depression-associated targets for each plant. These targets were used to construct protein–protein interaction networks and perform Gene Ontology and KEGG pathway enrichment analyses. For *L*. *leonurus*, the key targets included *SLC*_6_*A*_4_, *PTGS*_2_, *MAOA*, and *MAOB* were enriched in serotonergic and dopaminergic synapse pathways, neuroactive ligand–receptor interaction, and *PI*_*3*_*K/Akt* signalling, suggesting, suggesting potential involvement in neurotransmission and neuroinflammatory regulation. Molecular docking showed strong interactions of procyanidin B5 and chrysoeriol with *SLC*_6_*A*_4_ and *MAOA*, respectively, while baicalin demonstrated the most favourable binding affinity (− 39.45 kcal/mol), exceeding that of fluoxetine (− 14.69 kcal/mol), both surpassing fluoxetine’s affinity (− 14.69 kcal/mol) to *SLC*_6_*A*_4_. For *M. longifolia*, targets such as *DRD*_4_, *DRD*_3_, *GSK*_3_*β*, *COMT*, and *AKT*_*1*_ were linked to dopaminergic synapse and neuroactive ligand–receptor pathways. Key compounds, including baicalin, salvianolic acid A, and rosmarinic acid, showed strong binding to *DRD*_4_, *DRD*_3_, and *GSK*_3_*β*. Notably, quercetin 3-galactoside exhibited the highest affinity toward *DRD*_4_ (− 46.74 kcal/mol), outperforming fluoxetine (− 24.91 kcal/mol). Collectively, these findings suggest that both plants may possess antidepressant-related potential through predicted multi-component, multi-target modulation of neurotransmitter systems and neuroinflammatory pathways. However, these findings remain computational and require experimental validation to confirm biological efficacy and therapeutic relevance. This study provides a mechanistic framework to support future pharmacological investigations into the traditional use of these medicinal plants for mental health applications.

**Graphical abstract:**

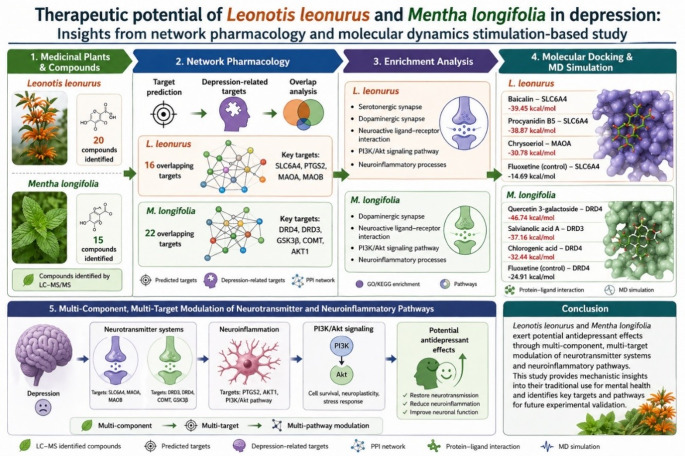

**Supplementary Information:**

The online version contains supplementary material available at 10.1007/s40203-026-00689-2.

## Introduction

Medicinal plants have long been used in traditional treatment systems, and they continue to provide exciting opportunities for the discovery of new neuroactive molecules. Their large reservoir of bioactive chemicals has spurred renewed scientific interest in verifying their therapeutic potential, notably in the modulation of neurochemical pathways linked to depression (Bonokwane et al. 2022). Determining the neuro-modulatory potential of these plants is critical, as depression remains a frequent and debilitating mental health illness characterized by persistent sorrow, lack of interest or pleasure, and different cognitive and physiological abnormalities (Stafford et al. 2008; Wang et al. 2019). Although current pharmacological treatments such as selective serotonin reuptake inhibitors (SSRIs), serotonin and norepinephrine reuptake inhibitors (SNRIs), and monoamine oxidase inhibitors (MAOIs) are effective for many patients, they are often limited by delayed onset of action and undesirable side effects (Short et al. 2018). As a result, there is a growing interest in exploring alternative and complementary treatment strategies, particularly those derived from medicinal plants traditionally used in ethnomedicine (Li et al. 2022; Tao et al. 2013). Several phytochemicals such as flavonoids, saponins, alkaloids, polyphenols, triterpenoids, and fatty acids have shown promising anxiolytic and antidepressant-like effects in preclinical studies (Phootha et al. 2022).

Among the medicinal plants gaining attention for their neuropharmacological potential are *Leonotis leonurus* and *Mentha longifolia* and have been widely recognized for their traditional use in the management of neurological and mood-related disorders (Bonokwane et al. 2022). For instance, *L*. *leonurus*, commonly known as wild dagga or lion’s ear, is native to southern Africa and traditionally used for its calming, sedative, and euphoric properties (Nsuala et al. 2015). Ethnobotanical reports highlight its use in alleviating stress, anxiety, and other nervous system conditions (Bonokwane et al. 2022). The plant is rich in bioactive constituents, including diterpenoids, flavonoids, and alkaloids, which are believed to contribute to its neuropharmacological effects (El-Ansari et al. 2009). Similarly, *M*. *longifolia*, commonly referred to as wild mint, is an aromatic medicinal plant widely distributed across Africa, Europe, and Asia (Al-Taie and Al-Kenane 2020). Traditionally, it has been used for its antispasmodic, antimicrobial, antioxidant, and calming properties (Anwar et al. 2019). *M*. *longifolia* has also been applied to relieve stress, insomnia, and mental fatigue (Elshamy et al. 2021). It contains diverse bioactive compounds, including phenolics, and flavonoids, which are known to interact with neurochemical pathways involved in mood regulation (Nazem et al. 2019).

Despite the promising therapeutic potential of the two plants, their underlying potential antidepressant mechanisms remain unexplored and lack scientific validation. In recent years, computational strategies such as network pharmacology and molecular modelling have emerged as powerful approaches to elucidate the complex interactions between bioactive compounds and molecular targets involved in disease pathways (Gan et al. 2021; Gundogdu et al. 2025; Yang et al. 2023). These computational approaches enable a systems-level understanding of pharmacological actions by identifying potential active compounds, their druggable targets, and associated signalling pathways (Zhang et al. 2025). Similar network pharmacology-based strategies have been successfully applied to elucidate antidepressant mechanisms of medicinal plants, including *Epicedium* (Dong et al. 2021), *Cannabis sativa* (Shi et al. 2021), *Xiaoyaosan* (Liu et al. 2021b) and *sinisan* formula (Wang et al. 2022a) revealing multi-target interactions involved in neurotransmission, neuroinflammation, and *PI3K/Akt* signalling pathways. Additionally, integrated approaches combining network pharmacology with compound screening have been used to identify antidepressant bioactives from *Banxia Houpu* decoction (Chen et al. 2020). This study explored potential mechanisms of antidepressant action of *L*. *leonurus* and *M*. *longifolia* using network pharmacology and molecular dynamics simulation approaches. By integrating compound-target-pathway analysis, the study provides insights into how these plants may exert their antidepressant effects and identifies promising targets for future pharmacological research.

## Materials and methods

### Plant collection and preparations

The leaves of *L*. *leonurus* were collected from the Botanical Garden of the University of KwaZulu-Natal, and thereafter taxonomically identified and authenticated (voucher number NU0094474) at the Bews Herbarium, University of KwaZulu-Natal. On the other hand, *M*. *longifolia* was collected from the Botanical Garden of North-West University. Its voucher specimen was authenticated (voucher number PUC0017098) and deposited in the AP Goossens Herbarium at North-West University. The plant materials (leaves) collected were rinsed with distilled water, oven-dried at 37 °C for 2 days, and ground into a fine powder, which was subsequently sieved to remove coarse particles. The powdered samples were then stored in air-tight containers at room temperature (away from sunlight and moisture) until further use.

### Liquid chromatography–mass spectrometry (LC–MS) analysis of *Leonotis leonurus* and *Mentha longifolia*

A powdered sample of 0.25 g each of the two plants was extracted in 10 mL of 50% methanol with 1% formic acid in water using vortexing and sonication. Following centrifugation, the supernatant was diluted fivefold with 50% methanol containing 0.1% formic acid and transferred to glass vials for analysis (Alam et al. 2017; Kongolo Kalemba et al. 2024). The analysis was then performed on a Waters Cyclic Quadrupole time-of-flight (q-tof) mass spectrometer (MS) connected to a Waters Acquity ultra-performance liquid chromatograph (UPLC) (Waters, Milford, MA, USA). The column eluate first passed through a Photodiode Array (PDA) detector, enabling simultaneous collection of UV and mass spectra. Electrospray ionization was conducted in both positive and negative modes, with a cone voltage of 15 V, a desolvation temperature of 275 °C, and a desolvation gas flow of 650 L/h. Other MS parameters were optimized for resolution and sensitivity. Data were acquired over an m/z range of 100–1500 in both resolution and MSE modes. In MSE mode, two data channels were collected: one at low collision energy (4 V) and another with a collision energy ramp of 40–100 V to generate fragmentation spectra. Leucine enkephalin served as the lock mass for accurate mass determination, and the instrument was calibrated using sodium formate. Chromatographic separation was achieved on a Waters HSS T3 column (2.1 × 150 mm, 1.7 μm) at 60 °C, with a flow rate of 0.3 mL/min and an injection volume of 0.5 µL. The mobile phase consisted of 0.1% formic acid in water (solvent A) and acetonitrile with 0.1% formic acid (solvent B). The gradient began at 100% A for 1 min, increased linearly to 28% B over 11 min, then to 40% B over 50 s, followed by a 1.5 min wash at 100% B and re-equilibration to initial conditions for 2 min. Compounds were quantified relatively using a calibration curve generated from ellagic acid standards (0.2–5 mg/L). Following centroiding, lock mass correction, and data compression, the resulting datasets were processed using MSDIAL and MSFINDER software (RIKEN Centre for Sustainable Resource Science, Kanagawa, Japan) (Lai et al. 2018; Tsugawa et al. 2015).

### Network pharmacology

#### Target prediction

Targets of compounds in *L*. *leonurus* and *M*. *longifolia* were predicted using their “Canonical SMILES” from the Swiss Target Prediction (http://swisstargetprediction.ch) and SEA (Similarity Ensemble Approach) (http://sea.bkslab.org/) databases, with the *Homo sapien* mode used to acquire the genes (Gao et al. 2020).

Depression-related target genes were identified using the DisGeNET (https://www.disgenet.org/search, retrieved on July 10, 2025) and GeneCards (https://www.genecards.org) databases (Piñero et al. 2020). The Venn mapping tool VENNY 2.1 (http://bioinformatics.psb.ugent.be/webtools/Venn/) was used to identify and intersect the shared target genes of *L*. *leonurus* and *M*. *longifolia* with depression-associated genes (Gao et al. 2024). The overlapping genes were examined as prospective target genes of *L*. *leonurus* and *M*. *longifolia* for the treatment of depression.

#### Construction of protein–protein interaction (PPI) networks

The common target genes were entered into the STRING database (https://string-db.org/, accessed July 10, 2025) (Szklarczyk et al. 2021), with the species parameter set to *Homo sapiens* (Gu et al. 2022). The network was subsequently analysed and visualized using the Cytoscape software (Otasek et al. 2019). The interactions between node representations were designated by edges, with nodes having a higher number of direct linkages suggesting greater biological significance (Gan et al. 2021).

#### Gene ontology (GO) enrichment and Kyoto encyclopaedia of genes and genomes (KEGG) pathway analysis

To study the probable mechanisms of *L*. *leonurus* and *M*. *longifolia* in depression treatment, pathway enrichment analysis was performed using DAVID 6.8 (https://david.ncifcrf.gov, accessed on July 10, 2025) (Dennis et al. 2003). Search parameters were set to *Homo sapiens*, with “OFFICIAL GENE SYMBOL” selected as the gene identifier. The resulting gene target list was compiled in a consistent format and subsequently analysed using the “functional annotation” tool. The analysis output was classified into three categories: biological processes (BP), cellular components (CC), and molecular functions (MF). Functions with *p* value < 0.05 were considered statistically significant for each category.

For pathway analysis, the common genes were submitted to STRING and analysed for KEGG pathway enrichment to identify pathways associated with the target genes of *L*. *leonurus* and *M*. *longifolia*. The resulting pathways were examined for direct relevance to depression. Pathways with the lowest false discovery rate (FDR < 0.05) were considered the most enriched and significantly associated with the intersecting targets. The enriched pathways were visualized using SRplot, a free online data analysis and visualization tool (http://www.bioinformatics.com.cn/en).

#### Construction of a compound target pathway network

A network model of components, targets, and signalling pathways was created using the Cytomerger plugin in Cytoscape v3.9.0 software. The Cytoscape Network Analyser plugin was employed to perform network topology analysis, where nodes represented metabolites of *L*. *leonurus* and *M*. *longifolia*, and pathway targets identified from KEGG enrichment analysis. Edges indicated interactions between nodes, illustrating the relationships between metabolites and their corresponding targets. A higher number of edges connecting nodes corresponded to a greater degree of interaction (Gan et al. 2021).

#### Ligand and receptor preparation for molecular docking

After identification of significantly enriched pathways through KEGG analysis, targets exhibiting the highest interaction frequencies with *L. leonurus* and *M. longifolia* metabolites were selected for molecular docking analysis. For *L. leonurus*, *SLC*_*6*_*A*_*4*_, *PTGS*_*2*_, and *MAOA*, and for *M. longifolia*, *DRD*_4_, *DRD*_3_, and *GSK*_3_β, were selected as key receptors based on their biological relevance, degree of interaction in the protein–protein interaction (PPI) network, and pathway enrichment analysis. The three-dimensional crystal structures of the receptors were retrieved from the Research Collaboratory for Structural Bioinformatics Protein Data Bank (RCSB PDB). The corresponding Protein Data Bank identifiers (PDB IDs) used for each receptor are reported in Table 4 to ensure reproducibility (Adams et al. 2017). Protein structures were prepared using UCSF Chimera version 1.14, whereby co-crystallized ligands, water molecules, and non-standard amino acid residues were removed to optimise receptor structures for docking. Polar hydrogens and appropriate atomic charges were added prior to docking, and receptor structures were energy-minimised to reduce steric clashes and improve structural stability (Brändén et al. 2014). The three-dimensional ligand structures of the active metabolites identified from *L. leonurus* and *M. longifolia* were retrieved from PubChem (accessed July 10, 2025) (Kim et al. 2019). Ligands were prepared through geometry optimisation and converted into docking-compatible formats using AutoDock-compatible procedures within PyRx/Open Babel. Protonation states were assigned under physiological conditions to ensure biologically relevant ligand conformations during docking simulations. To ensure docking at the active site, the grid box coordinates were adjusted to correspond to the established x-y-z positions identified in Discovery Studio version 2021. To ensure docking at biologically relevant binding pockets, grid boxes were centred according to the binding coordinates of co-crystallized ligands and validated active-site residues identified using Discovery Studio version 2021. Grid-box dimensions and x-, y-, and z-axis coordinates for each receptor are provided in Tables 3 and 4 (Liu et al. 2024). To validate the docking protocol, redocking/self-docking was performed using co-crystallized ligands, and root-mean-square deviation (RMSD) values below 2.0 Å were considered indicative of acceptable docking reproducibility (Buza et al. 2024).

#### Molecular dynamics simulation

Molecular dynamics (MD) simulations were conducted to further evaluate the stability, binding poses, and dynamic interactions of the top ligand–protein complexes identified during molecular docking. Each complex was parameterized using the AMBER FF18SB force field within the AMBER18 software suite implemented on the in-house computational platform (HEAL1361) hosted at the Centre for High Performance Computing (CHPC), Cape Town, South Africa. Ligand parameters and atomic partial charges were generated using ANTECHAMBER, employing the Restrained Electrostatic Potential (RESP) charge model and the General Amber Force Field (GAFF). All complexes were solvated within a TIP3P explicit water box, and Na⁺ and Cl⁻ counterions were added to neutralize the systems and maintain electrostatic stability (Sivaraman et al. 2026). Each simulation used a step size of 2 fs and was performed in the isobaric-isothermal ensemble (NPT) with randomised seeding, a temperature of 300 K, a constant pressure of 1 bar, and a Langevin thermostat with a collision frequency of 1.0 ps and a pressure-coupling constant of 2 ps. Trajectory analyses included root-mean-square deviation (RMSD), root-mean-square fluctuation (RMSF), solvent-accessible surface area (SASA), radius of gyration (ROG), and hydrogen-bond analysis to evaluate structural stability, flexibility, compactness, and ligand retention throughout the simulation period. Convergence of the simulations was assessed based on RMSD stabilization and the persistence of key ligand–protein interactions across the trajectory. Additionally, the Molecular Mechanics/Generalized Born Surface Area (MM/GBSA) approach was employed to estimate binding free energies of the ligand–protein complexes. Binding free-energy calculations were derived from trajectory snapshots extracted across the 150 ns simulation to improve statistical robustness of energy estimation. Because dense trajectory sampling was used, frame averaging was applied to minimize stochastic fluctuations and ensure convergence of MM/GBSA estimates. The overall binding free energy (ΔGbind) for each ligand–protein complex was subsequently calculated (Ylilauri and Pentikäinen 2013).

## Results and discussion

### Metabolite profiles based on liquid chromatography–mass spectrometry (LC–MS) analysis

The metabolite profiles of *L*. *leonurus* and *M*. *longifolia* as determined by Q-TOF-LC–MS are shown in Tables 1 and 2, respectively. The analysis revealed a diverse range of secondary metabolites, including flavonoids, phenolic acids, terpenoids, and fatty acids. A total of 20 compounds were identified in *L*. *leonurus* (Table 1, Supplementary Fig. S1).

**Table 1:** Metabolites of *Leonotis leonurus* as determined by quadrupole time-of-flight mass spectrometry liquid chromatography

No	Compound name	Retention time (min)	Class	m/z [M + H]
1	Alpigenoside	7.64	Flavonoids	431,19
2	Apiin	7.91	Flavonoids	563,14
3	Baicalin	9.52	Flavonoids	445,08
4	Chrysoeriol	11.55	Flavonoids	299,06
5	Genistein	11.30	Flavonoids	269,05
6	Isovitexin 2′′-O-glucoside	7.38	Flavonoids	593,15
7	Kaempferol glucuronide	8.80	Flavonoids	461,07
8	Procyanidin B5	11.01	Flavonoids	577,14
9	Geniposidic acid	5.29	Iridoid Glycosides	373,11
10	Loganic acid	6.03	Iridoid Glycosides	375,13
11	1α,3β,22R-Trihydroxyergosta-5,24E-dien-26-oic acid 3-O-β-D-glucoside 26-O-[β-D-glucosyl-(1→2)-β-D-glucosyl] ester	11.89	Steroidal	945,47
12	26-(2-Glucosyl-6-acetylglucosyl]-1,3,11,22-tetrahydroxyergosta-5,24-dien-26-oate	12.29	Steroidal	841,43
13	7-Epi-12-hydroxyjasmonicacid glucoside	7.42	Fatty Acid	387,17
14	Pinellic acid	11.96	Fatty Acid	329,23
15	(Z)-3-Hexenyl β-D-glucopyranoside	9.39	Fatty Acid	261,13
16	Dimethyl 2-galloylgalactarate	5.33	Phenolics	389,07
17	Theogallin	5.68	Phenolics	389,07
18	Broussonetine F	9.91	Phenolics	360,24
19	Gibberellin A53	12.14	Terpenoids	347,19
20	4-[(1R,2R,3 S,4 S)-1,2,4-trihydroxy-2,6,6-trimethyl-3-[(2 S,3R,4 S,5 S,6R)-3,4,5-trihydroxy-6-(hydroxymethyl)oxan-2-yl]oxycyclohexyl]but-3-en-2-one	7.78	Terpenoids	431,19

Fingerprinting of *M*. *longifolia* using LC–MS produced 15 compounds, primarily phenolic acids and flavonoids (Table 2, Supplementary Fig. S2).

**Table 2:** Metabolites of *Mentha longifolia* as determined by quadrupole time-of-flight mass spectrometry liquid chromatography

No	Compound name	Retention time(min)	Class	m/z [M + H]
1	Astragalin 7-rhamnoside	16.52	Flavonoids	593,15
2	Hesperetin	18.62	Flavonoids	301,07
3	Quercetin 3-galactoside	16.21	Flavonoids	463,08
4	3-O-*p*-Coumaroylquinic acid	9.47	Phenolics	337,09
5	Caffeoylmalic acid	9.04	Phenolics	295,04
6	Caftaric acid	7.36	Phenolics	311,04
7	Chlorogenic acid	7.99	Phenolics	353,08
8	Hydroxytyrosol 1-O-glucoside	7.40	Phenolics	315,11
9	Lithospermic acid B	19.26	Phenolics	717,14
10	Rosmarinic acid	16.3	Phenolics	359,07
11	Salvianolic acid A	18.48	Phenolics	493,11
12	Salvianolic acid K	14.90	Phenolics	555,11
13	Tryptophan	7.65	Amino Acids	203,08
14	(1 S,6R)-2-succinyl-6-hydroxycyclohexa-2,4-diene-1-carboxylic acid	9.86	Miscellaneous	239,05
15	Xanthochymusside	17.34	Terpenoids	735,15

According to the Q-TOF-LC-MS analysis of *L. leonurus*, the primary chemicals discovered were largely flavonoids 40%, whereas *M*. *longifolia* was mostly composed of phenols 60%. The presence of these phenolic compounds and flavonoids is particularly important because both classes of compounds are linked to a variety of biological functions (Dubey and Pandey 2023). Various phenolics are well known for their antioxidant, anti-diabetic, anti-inflammatory, and enzyme inhibitor activities (De Lima et al. 2025; Roshanak et al. 2016). Their antioxidant activity reduces oxidative stress, which has been linked to diabetes and neurodegeneration; their anti-inflammatory properties help control chronic inflammation, which is a cause of metabolic syndrome and depression; and their enzyme inhibitory capacity, particularly against monoamine oxidase and cholinesterases, promotes neurotransmitter balance in neurological disorders. These mechanism highlight their therapeutic promise in controlling metabolic and neurological disorders (Khade et al. 2023). Plants high in flavonoids, such as *L*. *leonurus* and *M*. *longifolia*, also provide additional potential therapeutic benefits, including neuroprotective, cardioprotective, and anti-inflammatory characteristics (Choy et al. 2019). Several phytochemicals found in *L*. *leonurus* and *M*. *longifolia*, as reported in preclinical research, have been shown to exhibit antidepressant-like properties (Silva dos Santos et al. 2021). Compounds found in *L*. *leonurus*, such as baicalin, genistein, kaempferol, and apigenin (the apiin aglycone), have been shown to alleviate depressive-like behaviours through mechanisms such as modulation of BDNF/TrkB signalling, HPA axis regulation, inhibition of monoamine oxidase, and suppression of neuroinflammation (Ekici et al. 2023; Fang et al. 2021). Iridoid glycosides structurally similar to loganin and loganic acid have also shown antidepressant action, adding to the medicinal usefulness of this species (Aktar et al. 2024). Similarly, with *M. longifolia *metabolites, hesperetin, quercetin, and its glycosides, chlorogenic acid, rosmarinic acid, salvianolic acid A, and tryptophan have been extensively studied for their antidepressant properties (Ghasemzadeh Rahbardar and Hosseinzadeh 2020; Paudel et al. 2018). These phytochemicals have antioxidant and anti-inflammatory properties, modulate monoamine neurotransmitters, regulate neurotrophic factors including BDNF, and restore stress-related neuroendocrine function (Young and Leyton 2002). Although direct antidepressant evidence is limited for some other phytochemicals identified in both plants, many exhibit strong neuroprotective, antioxidant, and enzyme-inhibitory properties, which may contribute synergistically to the overall antidepressant potential of *L*. *leonurus* and *M*. *longifolia.* The presence of these bioactive chemicals highlights the plants’ potential as multi-target medicines for addressing complicated illnesses including depression. The identified compounds in the two plants were subsequently used as ligands for the network pharmacology analysis.

### Network pharmacology

#### Target prediction

The Venn diagram analysis found 66 important overlapping targets for *L*. *leonurus* and 95 for *M*. *longifolia*. When compared to depression-associated genes, *L*. *leonurus* has 22 common targets, and *M*. *longifolia* has 16 common targets, respectively (Supplementary Figs. S3 and S4). These overlapping targets demonstrate the ability of both plants to influence depression-related pathways via various molecular interactions. The analysis revealed that common targets were highly implicated in serotonergic, dopaminergic, and cholinergic synapses. Targets related to inflammation and oxidative stress regulation, such as TNF signalling, NF-κB signalling, and IL-17 signalling, were shown to be enriched (Yang et al. 2024).

#### Protein-protein interaction (PPI) network analysis

The protein-protein interaction networks of 22 common targets (supplementary Fig. S5) for *L*. *leonurus* and 16 targets (Supplementary Fig. S6) for *M*. *longifolia* revealed complex and linked interactions, emphasising the multi-target nature for both plants. *L*. *leonurus* key hub targets were solute carrier family 6 member 4 (*SLC*_6_*A*_4_), Prostaglandin-Endoperoxide Synthase 2 (*PTGS*_2_), Monoamine Oxidase B (*MAOB*), and Monoamine Oxidase A (*MAOA*), whereas *M*. *longifolia* core targets included Dopamine Receptor D4 (*DRD*_*4*_), Glycogen Synthase Kinase 3 Beta (*GSK*_3_*β*), Catechol-O-Methyltransferase (*COMT*), Dopamine Receptor D3 (*DRD*_3_), Glutamate Ionotropic Receptor AMPA Type Subunit 1 (*GRIA*_1_), and Threonine Kinase 1 (*AKT*_1_). The protein-protein interaction (PPI) network analysis revealed complicated interactions between the target proteins of both plants, supporting the concept that their effects are mediated through complex and interconnected molecular networks. Furthermore, this integrated network analysis supports the notion that depression is a very complicated illness with various molecular and cellular pathways (Güleç et al. 2025). The ability of numerous bioactive compounds to interact with multiple targets shows the complex character of depression and reinforces the therapeutic promise of *L*. *leonurus* and *M*. *longifolia* in altering important biological processes related to its therapy.

#### Gene ontology (GO) enrichment analysis

The top ten terms for Molecular Function (MF), Cellular Component (CC), and Biological Process (BP) in the GO enrichment analyses are shown in supplementary Fig. S7. The neurotransmitter reuptake, transport, and lipid metabolism were the main enriched BP terms in *L*. *leonurus*, indicating a function in regulating synaptic signalling and neuronal communication. MF enrichment focused on protein binding and monoamine transmembrane transporter activity, whereas CC terms were concentrated in the presynaptic membrane, presynapse, and axon. These findings are in line with earlier research that demonstrated that compounds derived from plants, such as flavonoids and iridoids, can modulate neurotransmitter transport and synaptic function to produce effects similar to those of antidepressants (Supplementary Fig. S7) (Wang et al. 2022a).

In *M*. *longifolia*, BP enrichment includes chemical synaptic transmission and negative signalling control, with major CC keywords connected with the cell membrane and periphery (Supplementary Fig. S8). MF terminology emphasised postsynaptic neurotransmitter receptor activity, indicating potential regulation of receptor-mediated neural responses (Supplementary Fig. S8). These data are consistent with studies from other medicinal plants, including *Hypericum perforatum*, whose chemicals were shown to modulate postsynaptic receptors, synaptic signalling, and neurotransmitter receptor activity via serotonin transporters and postsynaptic receptors pathways relevant to depression (Wang et al. 2022a). Similarly, *Rosmarinus officinalis* extracts high in rosmarinic acid have been shown to influence synaptic plasticity and monoamine modulation, which is compatible with our findings in *M*. *longifolia* (Ghasemzadeh Rahbardar and Hosseinzadeh 2020). Flavonoids found in *L*. *leonurus*, including quercetin and kaempferol, have previously been linked to modulation of GABAergic and serotonergic neurotransmission, as well as neurotrophic signalling (Rebas et al. 2020).

Furthermore, GO keywords connected with lipid metabolism in *L*. *leonurus* overlap with research on polyphenol-rich plants such as *Camellia sinensis* and *Ginkgo biloba*, where changes in lipid-associated signalling cascades contributed to antidepressant-like effects (Ali et al., 2025). The receptor-mediated signalling enrichment in *M*. *longifolia* is consistent with studies from *Salvia miltiorrhiza*, where salvianolic acids bind postsynaptic neurotransmitter receptors and alter synaptic activity (Li et al. 2025). Overall, these findings suggest that the investigated plants are predicted to modulate depression-related pathways through interactions with molecular targets involved in neurotransmission, synaptic function, and neuronal signalling, warranting further experimental validation.

#### Kyoto encyclopedia of genes and genomes (KEGG) pathway enrichment

**Fig. 1:**
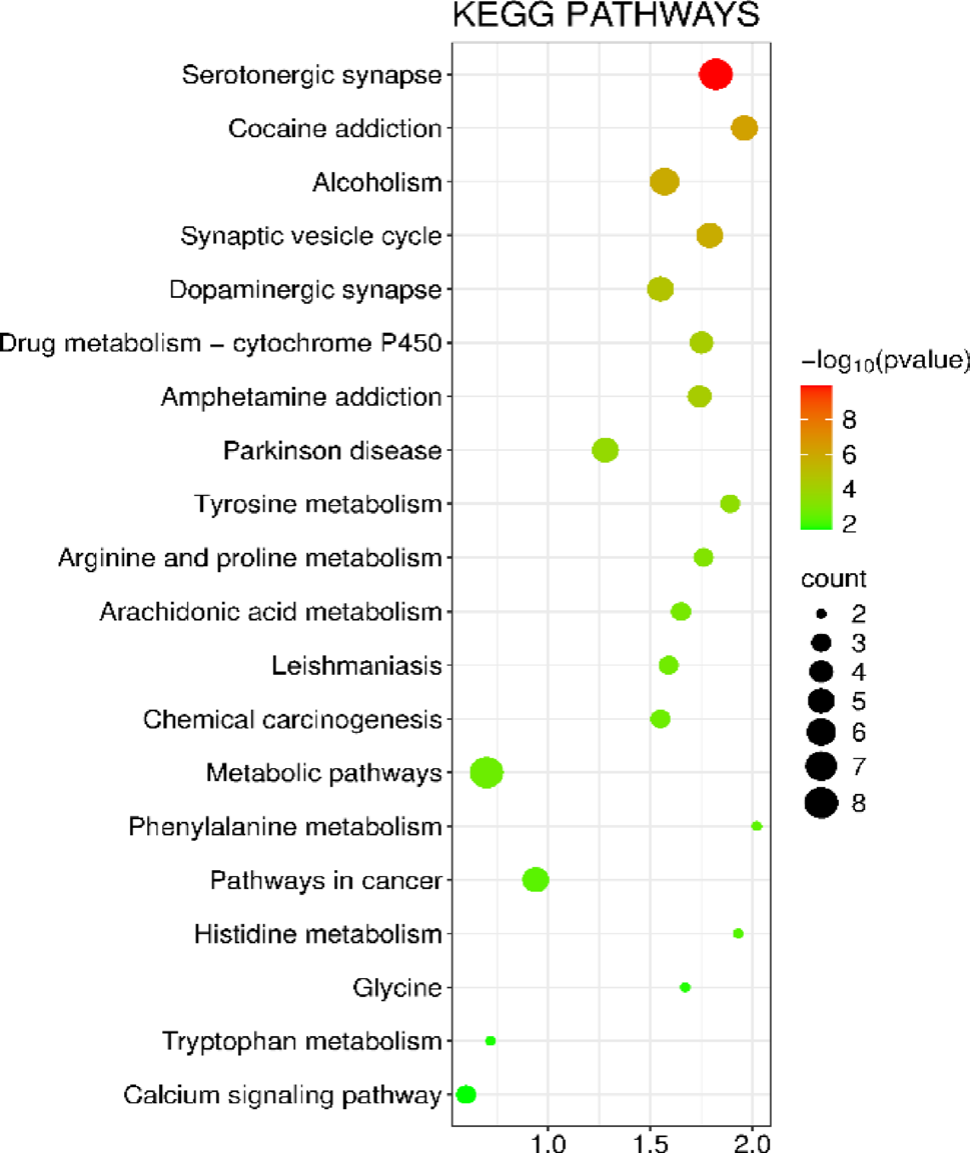
Enriched bubble plot of the top 20 KEGG pathways related to metabolites of *Leonotis leonurus*. The x-axis represents the gene ratio, defined as the proportion of input genes enriched in a specific pathway relative to the total number of input genes. The y-axis lists the KEGG pathways. Bubble size corresponds to the number of genes enriched in each pathway, while bubble color indicates statistical significance based on adjusted *p* values (false discovery rate, FDR), with darker shades indicating higher significance. Enrichment analysis was performed using a hypergeometric distribution model, and *p* values were corrected using the Benjamini–Hochberg procedure to control for false discovery. The enrichment factor reflects the ratio of observed gene hits to expected gene hits per pathway background

**Fig. 2:**
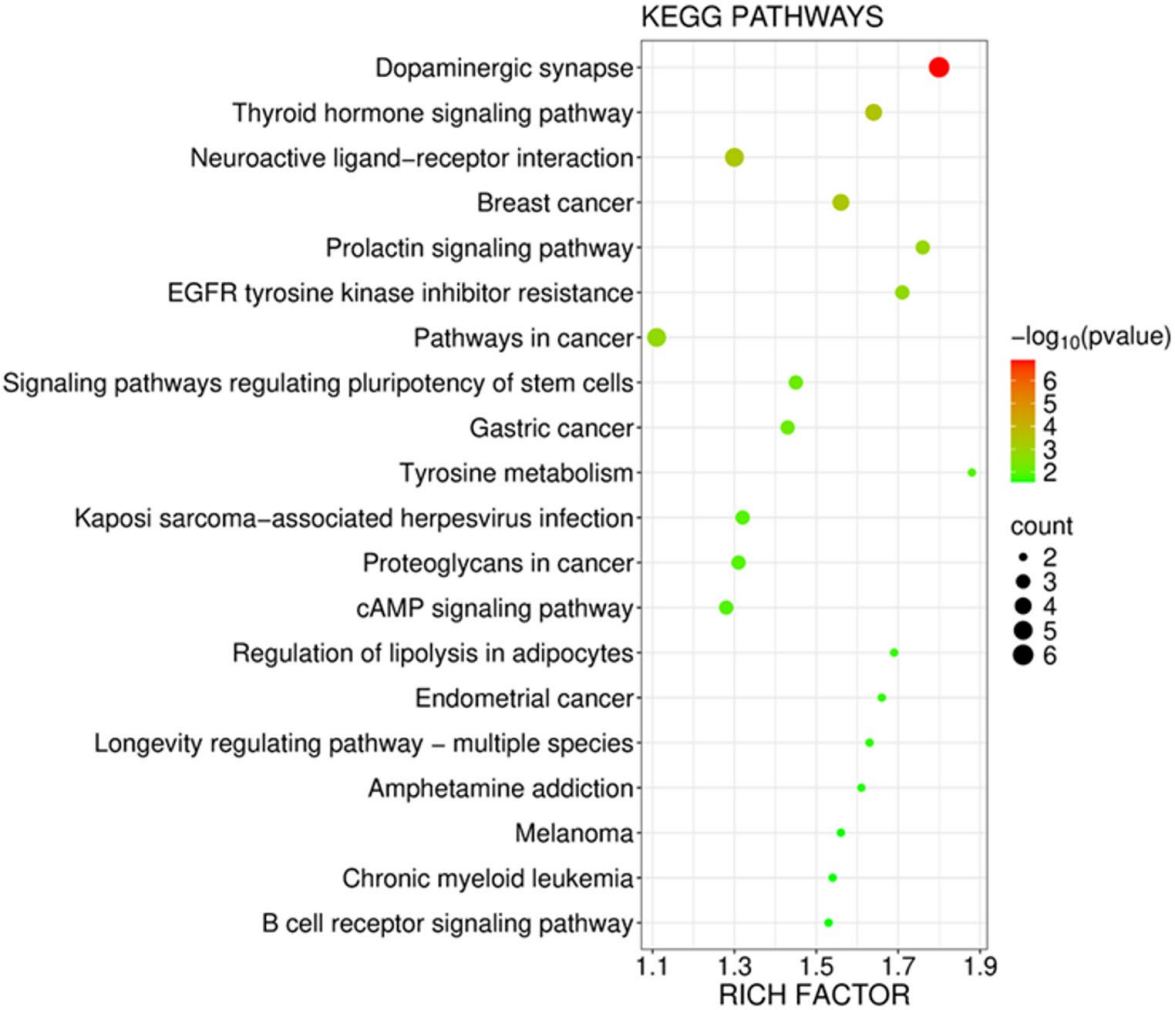
Enriched bubble plot of the top 20 KEGG pathways related to metabolites of *Mentha longifolia*. The x-axis represents the gene ratio, defined as the proportion of input genes enriched in a specific pathway relative to the total number of input genes. The y-axis lists the KEGG pathways. Bubble size corresponds to the number of genes enriched in each pathway, while bubble color indicates statistical significance based on adjusted *p* values (false discovery rate, FDR), with darker shades indicating higher significance. Enrichment analysis was performed using a hypergeometric distribution model, and *p* values were corrected using the Benjamini–Hochberg procedure to control for false discovery. The enrichment factor reflects the ratio of observed gene hits to expected gene hits per pathway background

To acquire a better understanding of the biological mechanisms involved, KEGG pathway enrichment analysis was used to connect the common targets to depression-related signalling pathways. The top 20 KEGG pathways with the lowest false discovery rate (FDR) values for *L*. *leonurus* and for *M*. *longifolia* are presented in Figs. 1 and 2, respectively. For *L*. *leonurus* the most significantly enriched pathways included the serotonergic synapse, cocaine addiction, and dopaminergic synapse pathways. Enrichment significance was evaluated based on *p* values and the number of genes involved (Fig. 1).

Similarly, for *M*. *longifolia*, the top 20 enriched pathways featured the dopaminergic synapse, neuroactive ligand–receptor interaction, and thyroid hormone signalling pathways (Fig. 2). Notably, most of the hub genes identified within the PPI network were associated with the dopaminergic synapse pathway, highlighting its potential central role in mediating the antidepressant effects of *M*. *longifolia.*

The serotonergic synapse pathway enriched in *L*. *leonurus*, plays a critical role in the pathology of depression, as modulation of serotonin levels is strongly associated with depression and anxiety disorders (Al-Khatib et al. 2022). This finding aligns with previous studies where plant-derived flavonoids and alkaloids were shown to enhance serotonergic transmission, thereby exerting antidepressant-like effects (Gholami et al. 2025; Mallick and Banerjee 2023). These pathway interactions suggest that *L*. *leonurus* may influence depression-related signalling cascades either by directly modulating neurotransmitter systems or by impacting neuroplasticity and cellular metabolism. Furthermore, metabolomic analysis of the hippocampus in a rat model of chronic unpredictable mild stress (CUMS)-induced depression revealed significant alterations in metabolites linked to the serotonergic, dopaminergic, and glutamatergic synapses, further supporting their involvement in the pathophysiology of depression (Gao et al. 2021; Liu et al. 2021a).

In this study, *M. longifolia* compounds were found to specifically target the depression related pathways through key genes *DRD*_4_, *GSK*_3_*B*,* COMT*,* DRD*_3_, *GRIA*_1_, and *AKT*_*1*_. *GSK*_3_*β* is essential for regulating neurogenesis, neuronal survival, and the secretion of pro-inflammatory cytokines such as *IL-1β*, *IL-6*, and *TNF-α*. Its inactivation, either via gene knockout or pharmacological inhibitors, has been shown to produce antidepressant-like effects in animal models, and disruptions in the *Akt/GSK*_3_*β* pathway have been observed in the brains of patients with depression (Shen et al. 2019; Yan et al. 2017).

Dopamine, a key monoamine neurotransmitter, plays a central role in the hypothalamus and pituitary gland and is critical for mood regulation and reward processing. Dysregulation of dopamine synthesis, release, reuptake, or metabolism has been closely linked to the pathophysiology of depression (Jatana et al. 2015). Dopamine deficiency can lead to downregulation of the dopamine transporter and upregulation of D_2_/_3_ receptor concentrations, as observed in the amygdala of patients with depression, indicating that altered dopaminergic signalling contributes to depressive phenotypes (Pritchard et al. 2009). Moreover, a reduction in dopaminergic neurons and dysfunction of dopamine receptors, including *DRD*_4_ and *DRD*_3_, are considered major risk factors for depression. *DRD*_4_, predominantly expressed in the prefrontal cortex, and *DRD*_3_, localized in the limbic system, are involved in regulating cognition, emotion, and reward-related behaviours, and their dysregulation may exacerbate depressive symptoms (Jatana et al. 2015). Polymorphisms in *DRD*_4_ are associated with altered dopaminergic signalling, heightened susceptibility to mood disorders, and variable responses to antidepressant treatment (Ferré et al. 2022). Similarly, *DRD*_3_, primarily expressed in the limbic system, modulates emotional processing and reward-related behaviours, and its dysregulation has been implicated in depressive phenotypes (Dannlowski et al. 2013). Targeting *DRD*_3_ may therefore help restore dopaminergic neurotransmission and alleviate depressive symptoms. The molecular docking results provide mechanistic support for the potential antidepressant effects of *M*. *longifolia*. Several bioactive compounds, including salvianolic acid A, rosmarinic acid, quercetin 3-galactoside, chlorogenic acid, hesperetin, and astragalin 7-rhamnoside, demonstrated stronger binding affinities to key depression-related targets, *DRD*_4_, *DRD*_3_, and *GSK*_3_*β* than the clinically used antidepressant fluoxetine. These findings suggest that compounds from *M. longifolia* may form stable predicted interactions with dopaminergic receptors and signaling-related proteins implicated in depression-associated pathways. The docking and molecular dynamics analyses indicate potential multi-target engagement, which may be relevant to neurotransmission and neuroinflammatory processes. However, these observations are based on computational predictions and should not be interpreted as evidence of confirmed neuropharmacological or antidepressant effects. Further in vitro and in vivo studies are required to validate the biological significance of these interactions.

#### Pathway compound target network

**Fig. 3:**
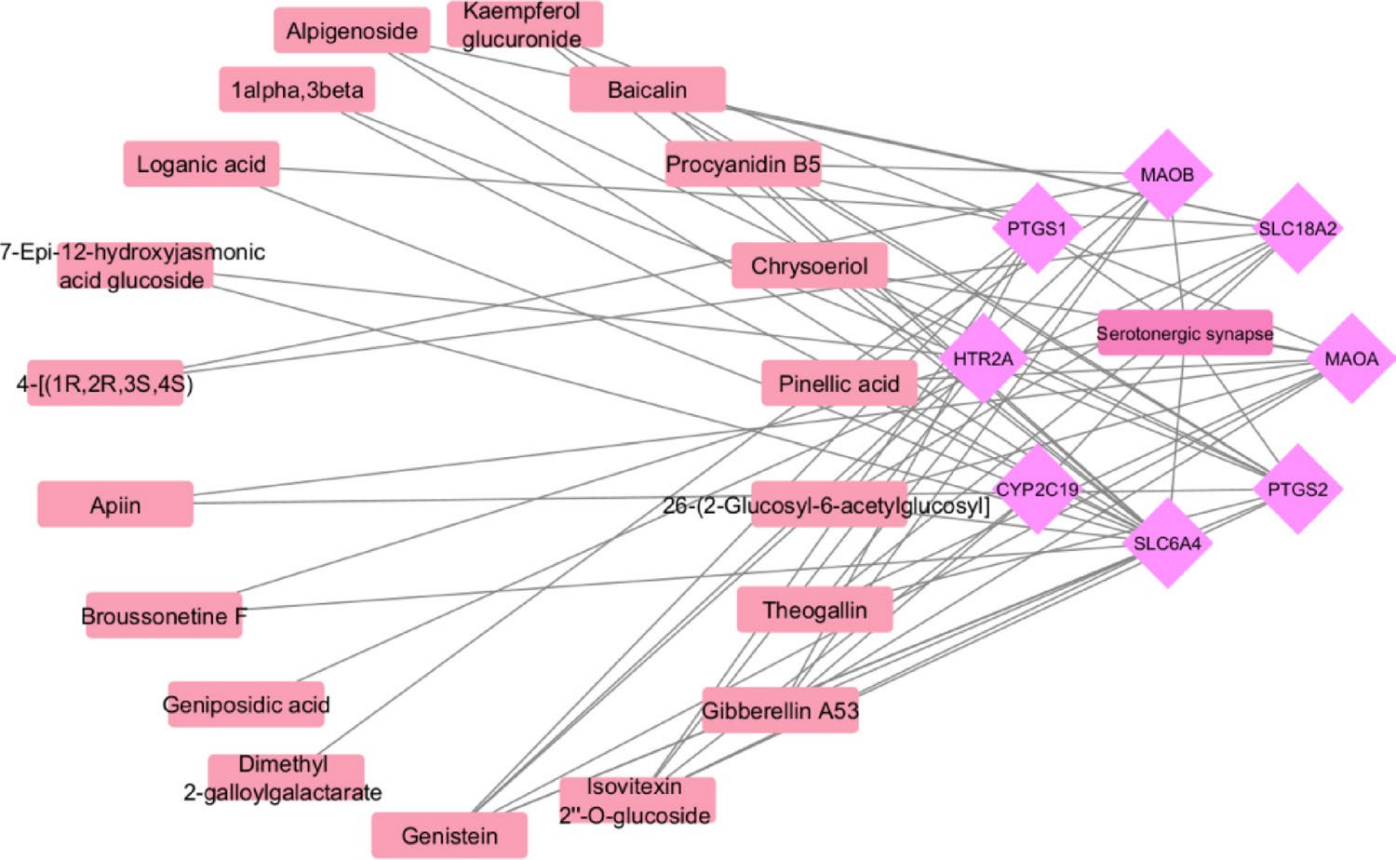
Protein-Protein Interaction (PPI) network derived from plant compounds associated with the pathway genes of *Leonotis leonurus*

*Leonotis leonurus* network search found *SLC*_6_*A*_4_ (11 compounds), *PTGS*_2_ (9 compounds), and *MAOA* (8 compounds) as major hub targets due to their high linkage to many metabolites (Fig. 3). These proteins are well-known regulators of neurotransmitter levels and neuroinflammation, highlighting their critical role in the plant’s possible antidepressant processes. Specifically, *SLC*_6_*A*_4_, which encodes the serotonin transporter, is critical for serotonergic synapse function by mediating serotonin reuptake from the synaptic cleft into presynaptic neurones, highlighting a mechanistic link between the bioactive compounds of *L*. *leonurus* and modulation of depression-related pathways (Abdelhamid et al. 2014). Several studies have linked *SLC*_6_*A*_4_ polymorphisms with the efficacy of antidepressant treatments. It is also the primary pharmacodynamic gene associated with selective serotonin reuptake inhibitors (SSRIs), the most commonly used class of antidepressants (Bi et al. 2020; Dong et al. 2009; Kautto et al. 2015). Additionally, *SLC*_6_*A*_4_ methylation has been found to be positively correlated with stress and depression, presenting its potential for diagnosis and treatment in major depressive disorders (Lockwood et al. 2022). A meta-analysis has shown that serotonin transporter availability is reduced in key limbic system areas of depressed patients compared to healthy controls (Kambeitz and Howes 2015). Cyclooxygenase-2 (*PTGS*_2_*/COX-*_2_), a crucial enzyme involved in prostaglandin synthesis, plays an important role in biological processes such as inflammation and pain (Kaur and Singh 2022). Evidence has shown that prenatal activation of COX-2 disrupts blood–brain barrier development, resulting in persistent neuroinflammation and increasing susceptibility to neuropsychiatric disorders (Zhao et al. 2022). *PTGS*_2_ is therefore recognized as a critical factor in the pathogenesis of such conditions. Numerous chemical agents and natural compounds have demonstrated antidepressant effects by modulating COX-2 activity, primarily through mechanisms involving neuroinflammation, gut microbiota regulation, neurotransmitter balance, HPA axis modulation, mitochondrial function, and protection against hippocampal neuronal injury (He et al. 2022). Oxidative stress and neuroinflammatory signaling pathways are increasingly recognized as key contributors to the pathophysiology of depression and other neuropsychiatric disorders. In this context, natural products that modulate redox balance and inflammatory mediators may offer multi-target therapeutic potential. Recent integrative approaches combining in silico and in vitro strategies have further emphasized the importance of evaluating compounds across multiple biological endpoints, including redox enzymes and inflammatory signaling cascades, to better predict pharmacological relevance (Zognjani et al. 2025). Such multiparametric evaluation frameworks support the view that plant-derived metabolites may exert broader systems-level effects rather than acting on single isolated targets.

Monoamine oxidases (*MAOs*), specifically *MAOA* and *MAOB*, are essential for regulating the levels of neurotransmitters, including serotonin and dopamine (Edmondson and Binda 2018). These enzymes are located on the outer membrane of mitochondria (Mathew et al. 2024). *MAOA* breaks down serotonin, noradrenaline, and adrenaline, making it a significant target in the treatment of depression due to its impact on these critical neurotransmitters (Shi et al. 2024).

**Fig. 4:**
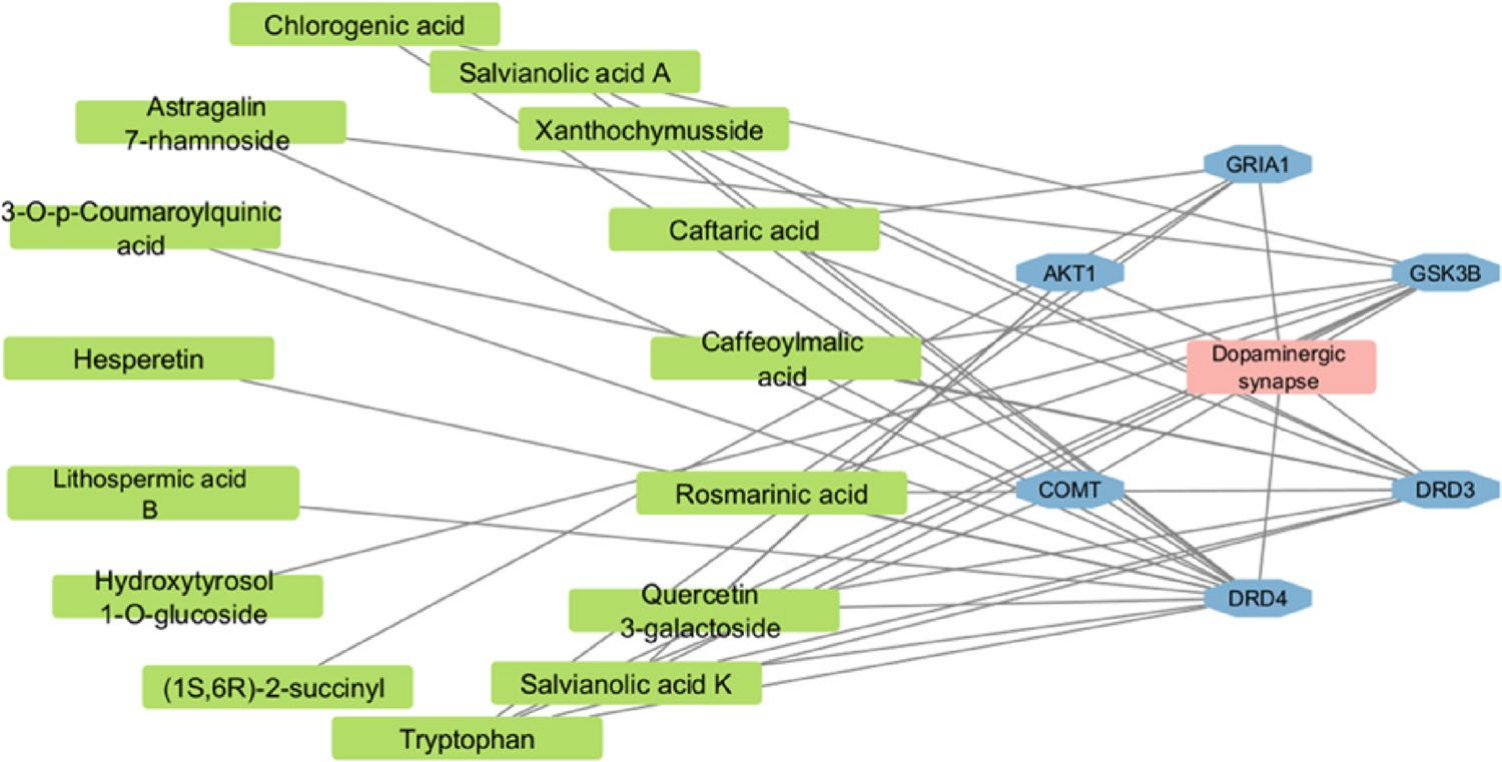
Protein-Protein Interaction (PPI) network derived from compounds associated with the pathway genes of *Mentha longifolia*

On the other hand, network analysis of *M*. *longifolia* (Fig. 4) constructed interactions among bioactive compounds, hub gene targets, and the significantly enriched KEGG pathway, specifically the dopaminergic synapse (Fig. 4). This network revealed that several compounds were closely associated with key hub genes, among which *DRD*_4_ (11 compounds), *DRD*_3_ (8 compounds), and *GSK*_3_*β* (7 compounds) emerged as essential targets, being implicated by most compounds. Collectively, these findings highlight the multi-component, multi-target nature of *M. longifolia* and its potential to modulate key molecular pathways associated with depression. The observed interactions suggest that its bioactive metabolites may act on multiple targets involved in neurotransmitter regulation and neuroinflammatory processes, thereby providing a mechanistic basis for further investigation of its neuropharmacological relevance.

The *DRD*_4_ receptor that is associated with the dopaminergic synapse pathway enriched in *M*. *longifolia* has been implicated in both schizophrenia and depression (Nakajima et al. 2007). In schizophrenia, postmortem and PET investigations have revealed elevated *DRD*_4_ expression in patient brain tissue, and particular exon polymorphisms have been related to the disorder, underlining its role in disease development (Chen and Shu 2006). Emerging evidence also points to *DRD*_4_*’s* involvement in depression: *DRD*_4_ mRNA serves as a peripheral marker of dopaminergic activity, is highly expressed in the amygdaloid nuclei, and is preferentially localized in GABAergic interneurons across brain regions involved in emotional processing, such as the prefrontal cortex, hippocampus, and thalamus (Xiang et al. 2008). Genetic studies further support this association, linking *DRD*_4_ repeat polymorphisms and exon III tandem repeat variations with susceptibility to depression (León et al. 2005). Collectively, these findings suggest that *DRD*_4_ plays a central role in modulating dopaminergic and emotional pathways relevant to both schizophrenia and depression.

*DRD*_3_, another dopamine receptor identified as a hub target in *M*. *longifolia*, modulates dopaminergic signalling in the limbic system. It is primarily expressed in the ventral striatum and globus pallidus, where it regulates dopamine release and clearance via the dopamine transporter (DAT) (Chang et al. 2013). This receptor is particularly enriched in limbic brain regions that receive dopamine from the ventral tegmental area and plays an important role in cognitive, affective, and endocrine function. Importantly, the Ser9Gly polymorphism of *DRD*_3_ has been widely related to major depressive illness, with Gly carriers demonstrating more anhedonia than Ser/Ser carriers, implying a molecular relationship to depression pathophysiology (Lee et al. 2012). Recent research suggests that this polymorphism is linked to other neuropsychiatric diseases such as bipolar disorder, anxiety, and Parkinson’s disease, influencing reward pathways and emotional behaviour (Korhonen et al. 2014).

Glycogen synthase kinase-3 beta (*GSK*_3_*β*) is a multifunctional serine/threonine kinase and downstream component of the Wnt signalling pathway that is well known for its role in depression and schizophrenia. *GSK*_3_*β* is involved in a variety of signalling cascades, including Wnt and *PI*_*3*_*K/Akt*, and is essential for neuroplasticity, circadian rhythm modulation, and mood stabilisation. *GSK*_3_*β* dysregulation has been linked to depression pathophysiology and inhibiting it is thought to be one of the mechanisms by which many antidepressants work (Chen et al. 2015). The Wnt/*GSK*_3_β pathway is crucial for the correct development of the foetal forebrain, including the hippocampus and midbrain dopaminergic systems brain regions frequently damaged in mood disorders such as bipolar disorder and other psychiatric diseases (Ripke et al. 2011).

Previous research has demonstrated that inhibiting *GSK*_3_ activity can improve antidepressant efficacy, and the mood stabiliser lithium directly inhibits the *GSK*_3_*β* enzyme in vitro and in human peripheral blood mononuclear cells (Diniz et al. 2011). Furthermore, lower *GSK*_3_*β* phosphorylation has been seen in platelets of depressive patients and Alzheimer’s disease patients who exhibit depressive symptoms, suggesting the link between *GSK*_3_*β* overactivity and mood disorders. Creatine’s antidepressant-like effects in the tail suspension test appear to entail activation of the *PI*_*3*_*K/Akt* pathway, downstream targets such as Nrf2/HO-1, GPx, mTOR, and concurrent suppression of *GSK*_3_*β* (Zhang et al. 2010).

### Molecular docking with the enriched genes

Molecular docking was performed to evaluate the binding affinity of phytochemicals from *L. leonurus* and *M. longifolia* against key depression-related targets identified through PPI network construction, compound–target mapping, and KEGG pathway analysis. Fluoxetine, a clinically established selective serotonin reuptake inhibitor (SSRI), was used as a reference standard to benchmark predicted binding interactions. Docking calculations were carried out using PyRx to predict ligand–receptor conformations and binding energies. Lower docking scores indicate stronger binding affinity and greater stability of the protein–ligand complex. Target selection was guided by their centrality in the PPI network, biological relevance to neuropsychiatric pathways, and availability of high-quality crystal structure (Gao et al. 2020). Fluoxetine was used as a positive control. Fluoxetine was included as a reference ligand across all docking experiments to ensure methodological consistency and enable comparative evaluation of binding affinities across different target proteins. As a well-characterized selective serotonin reuptake inhibitor (SSRI) with established clinical efficacy in the treatment of depression and anxiety disorders, fluoxetine provides a standardized benchmark for interpreting the relative binding strengths of the investigated phytochemicals. Its use as a common positive control allows for direct comparison of ligand–receptor interactions across serotonergic, dopaminergic, inflammatory, and neuroplasticity-related targets, thereby improving the reliability and interpretability of the docking results (Choudhary et al. 2020). Only compounds with strong predicted relevance to hub targets and available structural compatibility were selected for docking analysis. For *L*. *leonurus*, docking results (kcal/mol) against *SLC*_*6*_*A*_*4*_ (PDB ID: 6DZV), *PTGS*_*2*_ (PDB ID: 5IKQ), and *MAOA* (PDB ID: 2BXR) are displayed in Table 3. The results revealed strong binding affinities across all targets (Table 3). Compounds including procyanidin B5, baicalin, chrysoeriol, kaempferol glucuronide, apiin, and isovitexin 2′′-O-glucoside demonstrated binding energies comparable to or stronger than fluoxetine, indicating potential pharmacological relevance in depression-related pathways. Notably, procyanidin B5 showed the strongest binding (–9.5 kcal/mol) with *SLC*_*6*_*A*_*4*_ in the serotonergic pathway, surpassing fluoxetine (− 8.5 kcal/mol), suggesting a possible role in serotonergic reuptake modulation. Similarly, baicalin demonstrated stronger binding to *PTGS*_*2*_ (− 6.5 kcal/mol) than fluoxetine (− 5.3 kcal/mol), while chrysoeriol exhibited higher affinity for *MAOA* (– 9.5 kcal/mol) compared to fluoxetine (− 9.0 kcal/mol), reinforcing the potential of these compounds in modulating monoamine metabolism and inflammatory pathways. In a recent epilepsy-network pharmacology study, baicalin docked to *PTGS*_*2*_ with a score of − 10.1 kcal/mol, suggesting that similarly structured flavonoids can attain stronger affinities under optimised conditions (Wang et al. 2022b). The current results are consistent with previous reports that these active components of *L*. *leonurus* directly inhibit *PTGS*_2_-mediated inflammatory signalling. In another investigation using *Si-Miao* decoction, baicalein bound *PTGS*_2_ at − 8.4 kcal/mol, MMP9 at − 8.0 kcal/mol, and *PPARG* at − 7.9 kcal/mol (Ma et al. 2024). Similarly, the strong binding of procyanidin-type compounds in published studies (e.g., procyanidin vs. SARS-CoV-2 Mpro at − 9.132 kcal/mol) suggests that the procyanidin B5 → SLC_6_A_4_ binding affinity we observe (− 9.5 kcal/mol) is within the realm of other successful natural product docking studies (Fatriansyah et al. 2022). Despite the promising molecular interactions and predicted antidepressant-related mechanisms observed for flavonoids such as baicalin and procyanidin B5 present in *L. leonurus*, their translation into in vivo therapeutic applications may be constrained by pharmacokinetic limitations and poor bioavailability. Flavonoids often exhibit low oral bioavailability due to poor intestinal absorption, extensive first-pass metabolism, and rapid systemic elimination (Chen et al. 2022). Additionally, limited blood–brain barrier permeability may restrict central nervous system exposure, which is essential for antidepressant efficacy. Baicalin has been reported to possess poor aqueous solubility, limited permeability, and extensive gastrointestinal metabolism, all of which may affect systemic availability. Likewise, procyanidins generally demonstrate low bioavailability due to limited absorption and extensive metabolic transformation following oral administration (Huang et al. 2019; Zhang et al. 2016). Therefore, although these compounds demonstrated promising in silico affinity toward depression-related targets, further in vivo pharmacokinetic and efficacy studies are required to validate their antidepressant potential. Future approaches such as nanoformulations or targeted drug delivery systems may improve bioavailability and enhance therapeutic applicability.

**Table 3:** Molecular docking scores (kcal/mol) of *Leonotis leonurus* compounds with key depression-related targets (*SLC*_*6*_*A*_*4*_, *PTSG*_*2*_, and *MAOA*) compared to the positive control, fluoxetine

Target	Active compound	Docking scores (kcal/mol)
*SLC*_*6*_ *A*_*4*_ **PDB ID: 6DZV** Dimensions **X**:31.11; **Y**:26.6;**Z**:29.23	Fluoxetine	− 8.5
	Procyanidin B5	− 9.5
	Chrysoeriol	− 9.3
	Baicalin	− 9.2
	Kaempferol glucuronide	− 9.1
	Apiin	− 9.1
	Genistein	− 8.7
	Loganic acid	− 8.3
	Theogallin	− 8.2
	Isovitexin 2′′-O-glucoside	− 8.1
	Broussonetine F	− 6.8
*PTSG*_*2*_ **PDB ID: 5IKQ** Dimensions **X**:16.17; **Y**:18.93.84;**Z**:17.83	Fluoxetine	− 5.3
	Baicalin	− 6.5
	Apiin	− 6.4
	Isovitexin 2′′-O-glucoside	− 5.6
	Genistein	− 5.2
	Chrysoeriol	− 5.2
	Kaempferol glucuronide	− 5.2
	Theogallin	− 5.0
	Alpigenoside	− 4.9
*MAOA* **PDB ID: 2BXR** Dimensions **X**:24,22; **Y**:32.50;**Z**:32.62	Fluoxetine	− 9.0
	Chrysoeriol	− 9.5
	Gibberellin A53	− 8.9
	Genistein	− 8.8
	Pinellic acid	− 8.6
	Theogallin	− 8.4
	Apiin	− 7.0
	Kaempferol glucuronide	− 6.6

For *M*. *longifolia*, docking analysis against dopaminergic receptors (*DRD*_4_ (PDB ID: 6IQL); *DRD*_3_ PDB ID: 3PBL), and *GSK*_3_*β* (PDB ID: 1UV5) revealed strong binding affinities across all targets (Table 4). Several compounds, including salvianolic acid A, rosmarinic acid, chlorogenic acid, and quercetin derivatives, consistently outperformed fluoxetine across multiple targets. Among the tested compounds, Salvianolic acid A showed the strongest binding to *DRD*_3_ (–9.5 kcal/mol), while rosmarinic acid also demonstrated strong binding to *DRD*_3_ (– 9.0 kcal/mol) and *GSK*_3_*β* (– 8.7 kcal/mol). Chlorogenic acid and quercetin 3-galactoside demonstrated high binding scores against *DRD*_4_ (PDB ID: 6IQL) and *GSK*_3_*β* (PDB ID: 1UV5) indicating multi-target potential within dopaminergic and kinase-mediated signalling pathways. These results are broadly compatible with prior in-silico studies on related chemicals. Salvianolic and rosmarinic acids have been discovered as potential *GSK*_*3*_*β* inhibitors in docking studies of Alzheimer’s disease models, frequently occupying the ATP-binding pocket and generating stable hydrogen-bond interactions with catalytic residues. In one investigation, salvianolic acid was shown to penetrate extensively into the *GSK*_3_*β* cavity, but rosmarinic acid had binding energies ranging from − 8.0 to − 9.0 kcal/mol, which are similar to the values observed here (Zareei et al. 2024). Similarly, salvianolic acid B, a structural counterpart, was discovered to create numerous hydrogen bonds within *GSK*_3_*β*’s ATP pocket, despite having a lower reported binding energy than typical kinase inhibitors (Marafie et al. 2024).

**Table 4:** Molecular docking scores (kcal/mol) of *Mentha longifolia* compounds with key depression-related targets (*DRD*_4_, *DRD*_3_, and *GSK*_3_*β*) compared to the positive control, fluoxetine

Target	Active compound	Docking scores (kcal/mol)
*DRD*_4_ **PDB ID**: 6IQL Dimensions **X**:31.25; **Y**:30.31;**Z**:29.32	Fluoxetine	− 7.6
	Salvianolic acid A	− 8.4
	Quercetin 3-galactoside	− 8.2
	Chlorogenic acid	− 8.0
	Hesperetin	− 8.0
	3-O-p-Coumaroylquinic acid	− 7.9
	Salvianolic acid K	− 7.7
	Astragalin 7-rhamnoside	− 7.6
	Caffeoylmalic acid	− 7.5
	Caftaric acid	− 7.0
	Tryptophan	− 7.7
*GSK*_3_ *β* **PDB ID: 1UV5** Dimensions **X**:34.27; **Y**:35.00;**Z**:35.24	Fluoxetine	− 8.1
	Rosmarinic acid	− 8.7
	Astragalin 7-rhamnoside	− 8.7
	Quercetin 3-galactoside	− 8.4
	Chlorogenic acid	− 8.4
	Salvianolic acid K	− 8.1
	Hydroxytyrosol 1-O-glucoside	− 7.7
	Caffeoylmalic acid	− 7.2
*DRD*_3_ **PDB ID**: 3PBL Dimensions **X**:32.32; **Y**:31.28;**Z**:27.28	Fluoxetine	− 7.8
	Salvianolic acid A	− 9.5
	Rosmarinic acid	− 9.0
	Salvianolic acid K	− 8.7
	3-O-p-Coumaroylquinic acid	− 8.0
	Caffeoylmalic acid	− 7.3
	Caftaric acid	− 7.2
	Quercetin 3-galactoside	− 7.0
	Tryptophan	− 6.8

Molecular docking results indicate that both *Leonotis leonurus* and *Mentha longifolia* exhibit multi-target antidepressant potential. *L. leonurus* compounds showed strong binding to serotonergic, inflammatory, and monoamine-related targets (SLC_6_A_4_, PTGS_2_, MAOA), while *M. longifolia* phytochemicals demonstrated high affinity for dopaminergic receptors (*DRD*_4_ and *DRD*_3_) and GSK3β, suggesting roles in dopamine signalling and synaptic plasticity. Collectively, the two plants may exert complementary antidepressant effects through serotonergic, dopaminergic, inflammatory, and neuroplasticity-related pathways. Redocking validation yielded an RMSD of 0.50 Å, confirming the reliability and reproducibility of the docking protocol. The docking validation results are presented in the supplementary materials (Supplementary Figs. S9–S14) (Buza et al. 2024). However, these predictions are primarily based on in silico approaches, and molecular docking and network pharmacology studies alone are insufficient to confirm pharmacological activity without experimental validation (Gundogdu et al. 2025).

### Binding free energy (MM-GBSA) and energy decomposition analysis for *SLC*_*6*_*A*_*4*_ and the top three ligands of *Leonotis leonurus*

Interactions between proteins and ligands often involve electrostatic, van der Waals, and hydrophobic forces (Ferenczy and Kellermayer 2022) forming complexes that require atomic-level examination to understand recognition and structure-activity relationships. Among analyzed systems, baicalin (− 39.45 kcal/mol) showed the most favorable binding energy, outperforming procyanidin B5 and chrysoeriol (− 38.87 and − 30.78 kcal/mol), and even fluoxetine (− 14.69 kcal/mol). Baicalin’s superior binding is due to strong electrostatic and van der Waals forces, offsetting energetic loss from solvation. Its binding to SLC6A4 displaced water molecules, disrupting solvation shells and causing an entropic penalty in ΔGsolv values, especially as water is confined in the binding site. These losses were balanced by enthalpic gains from stable water-mediated hydrogen bonds, notably in baicalin, which had high hydrogen-bond counts. Similarly, baicalein, a derivative of baicalin, showed van der Waals (− 44.37 kcal/mol) and electrostatic (− 17.84 kcal/mol) contributions that offset polar (20.69 kcal/mol) and nonpolar (5.72 kcal/mol) effects, affecting its overall binding free energy (− 46.56 kcal/mol) compared to chrysin (− 12.89 kcal/mol) (Li et al. 2022). Energy decomposition revealed that van der Waals interactions support aromatic stacking, while electrostatic forces promote hydrogen bonds and polar solvation among the top metabolites (Table 5).

**Table 5:** The energy component details for the top three compounds and fluoxetine complexed with *SLC*_*6*_*A*_*4*_

Energy components (kcal/mol)					
	ΔE_vdW_	ΔE_elec_	ΔG_gas_	ΔG_solv_	ΔG_bind_
Fluoxetine_*SLC*_6_*A*_4_	− 17.12 ± 4.68	− 28.42 ± 95.82	− 45.54 ± 97.37	30.85 ± 93.23	− 14.69 ± 8.80
Baicalin_ *SLC*_*6*_*A*_*4*_	− 34.26 ± 3.83	− 57.40 ± 11.47	− 91.67 ± 10.59	52.22 ± 9.11	− 39.45 ± 4.05
Procyanidin B5_ *SLC*_*6*_*A*_*4*_	− 38.85 ± 6.88	− 42.38 ± 14.04	− 81.24 ± 14.52	42.36 ± 8.86	− 38.87 ± 6.75
Chrysoeriol_ *SLC*_*6*_*A*_*4*_	− 37.42 ± 3.16	− 13.04 ± 7.01	− 50.47 ± 7.62	19.69 ± 6.07	− 30.78 ± 3.37

The three metabolites bind better than fluoxetine, likely because flavonoids’ planar aromatic rings facilitate π–π stacking with transporter amino acids (Vikhar Danish Ahmad et al. 2024). These compounds engage in hydrophobic, hydrogen-bonding, and π-stacking interactions, while fluoxetine mainly relies on hydrophobic contacts. Flavonoids also have antioxidant and neuroprotective roles, with baicalin reducing oxidative stress and modulating neurotransmitters, and chrysoeriol promoting anti-inflammatory effects by increasing Nrf2 and related genes (Kim et al. 2021; Wang et al. 2024; Zhong et al. 2019). Flavonoids influence monoaminergic neurotransmission, including serotonin, providing structural stability, neuroprotection, and multi-target effects, thereby supporting their potential for in vivo benefits and aligning with computational findings (Naoi et al. 2025).

**Fig. 5:**
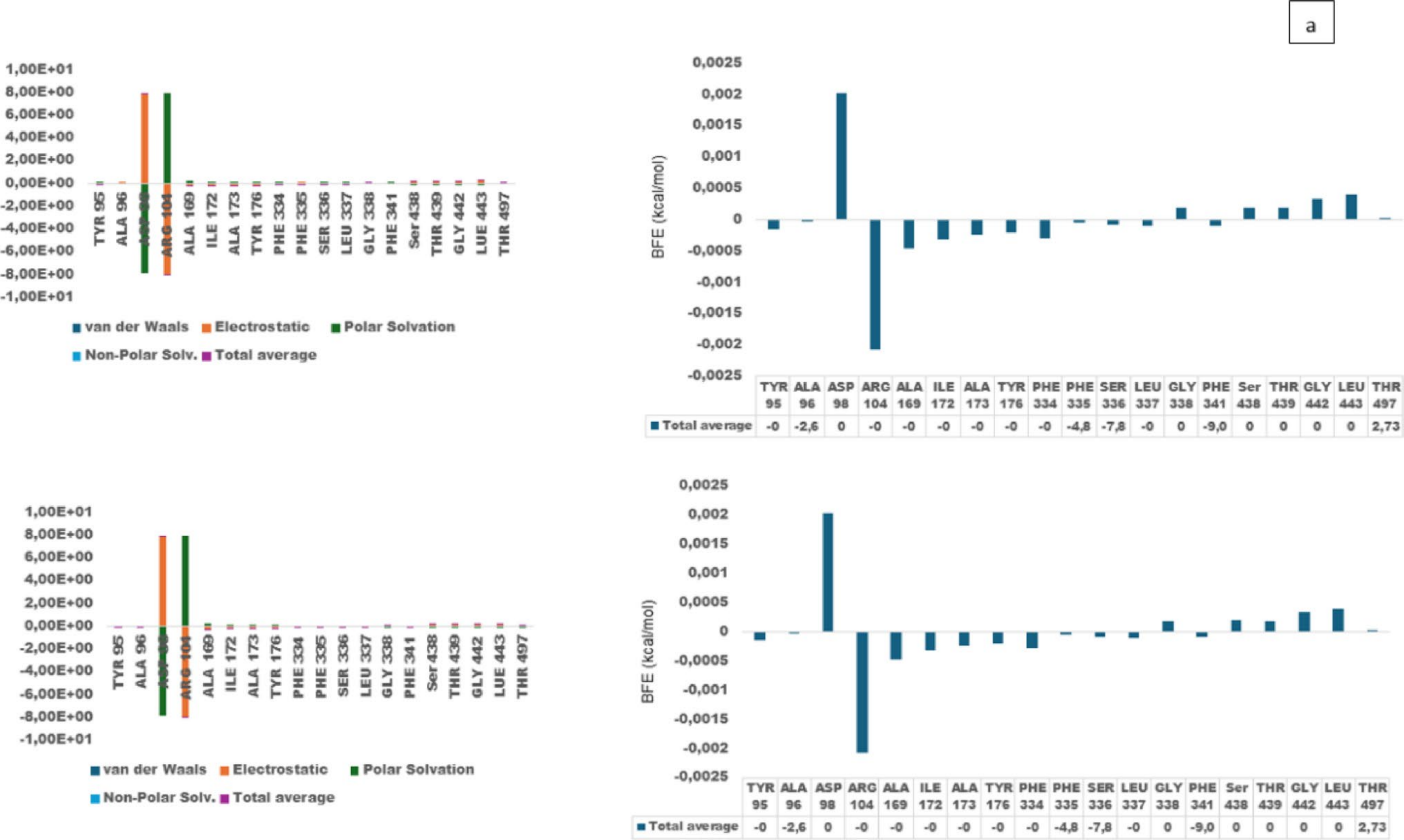
Residue decomposition for **a** Fluoxetine_*SLC*_*6*_*A*_*4*_ and **b** Baicalin_ *SLC*_*6*_*A*_*4*_

**Fig. 6:**
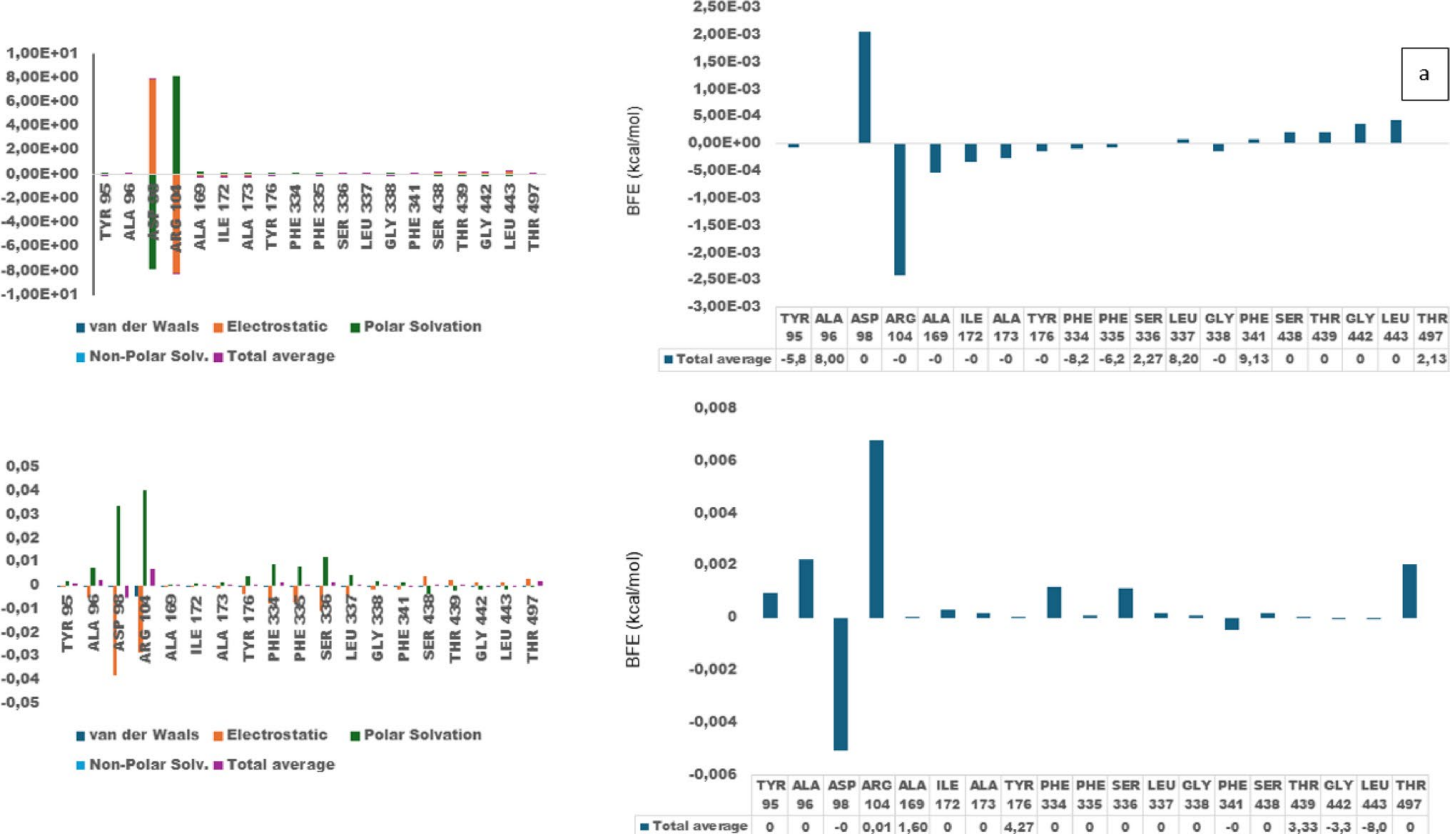
Residue Decomposition for **a** Procynaidin B5_*SLC*_*6*_*A*_*4*_ and **b** Chrysoeriol_*SLC*_*6*_*A*

Fluoxetine, though hydrophobic, lacks the aromatic surface area and polar interaction capacity of other antidepressants, did not form hydrogen bonds (Fig. S9), and could not stabilize aromatic stacking, electrostatic, and polar interactions. Its weak interactions lead to a more dynamic complex, thereby negatively impacting binding free energy, indicating that its therapeutic effects are mainly due to pharmacokinetics and serotonin reuptake inhibition, rather than strong receptor binding (Fig. S9, Table 5). Hence, natural metabolites such as baicalin, chrysoeriol, and procyanidin B5 could be promising scaffolds for new antidepressant or anxiolytic drugs. Binding free-energy calculations confirmed trends in their structural analyses at a specific simulation time, with energy decomposition revealing atomic-level structure-activity interactions. Key residues involved in these ligand-binding interactions include ALA96, ASP98, ARG104, LEU443, and GLU301, with the top three metabolites mainly depending on these residues (Figs. 5 and 6).

This differs from fluoxetine, which involves PHE335, PHE341, and SER336, with ALA96 as the only common residue. In the baicalin complex, 15 and 14 residues formed contacts at different timepoints, with ALA96 contributing significantly to binding energy, along with electrostatic and van der Waals interactions from ASP98 and ARG104 (Figs. 5b and S9) (Case et al. 2023; Kulikov et al. 2018).

Procyanidin B5_*SLC*_*6*_*A*_*4*_ also made notable energy contributions through TYR95 (− 5.8 kcal/mol), PHE334 (− 8.2 kcal/mol), and PHE335 (− 6.2 kcal/mol), with a total of 20 interactions (Figs. 6a, S10). This is important because TYR95 helps orient serotonin in the binding pocket (Coleman et al. 2016). LEU443 contributed the most to the binding energy of Chrysoeriol_*SLC*_*6*_*A*_*4*_ (− 8.0 kcal/mol) and formed 16 interactions (Figs. 6b, S11). Conversely, Fluoxetine_*SLC*_*6*_*A*_*4*_ showed important energy contributions from ALA96 (− 2.6 kcal/mol), PHE335 (− 4.8 kcal/mol), PHE341 (− 9.0 kcal/mol), and notably SER336 (− 7.8 kcal/mol), a chloride-binding amino acid (Figs. 5b , S12). However, they formed only seven interactions at 50 ns, decreasing to five at 100 ns and 150 ns. Nonetheless, it is crucial to analyse additional post-dynamics parameters, such as RMSD, RMSF, ROG, SASA, and H-Bond, to further confirm the stability and compactness of the complex between these top metabolites and the protein.

### Structural stability of the resulting complexes

**Fig. 7:**
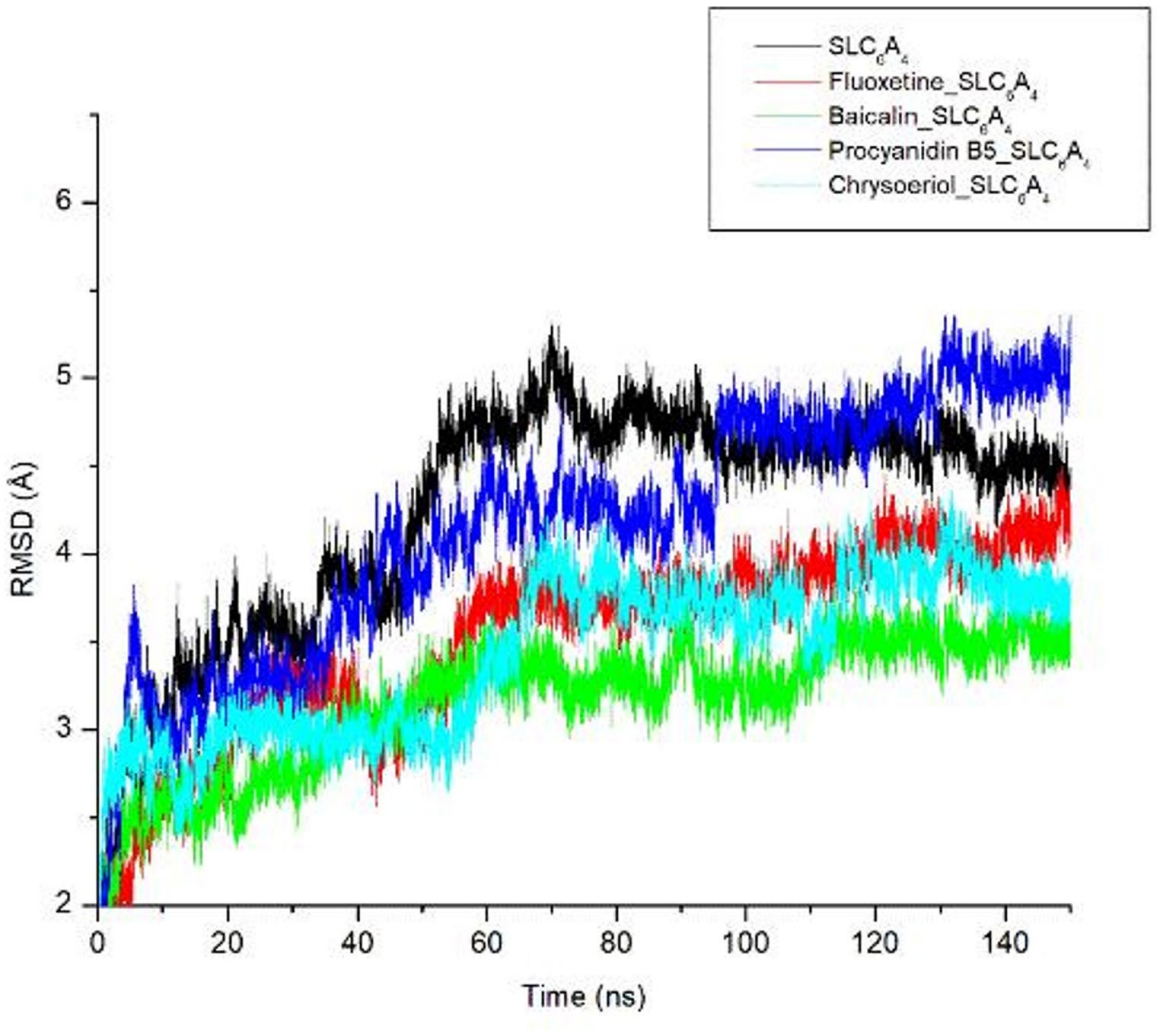
Backbone RMSD profile of *SLC*_*6*_*A*_*4*_–ligand complexes during 150 ns molecular dynamics simulation

The RMSD of C-α backbone atoms of SLC6A4 complexes with baicalin, chrysoeriol, procyanidin B5, and fluoxetine was monitored over 150 ns. The fluoxetine complex reached equilibrium after initial equilibration, validating the protocol. All complexes stabilized within 10 ns (Fig. 7), but differences arose around 6 ns. Baicalin and chrysoeriol exhibited lower, more stable RMSD values than fluoxetine, indicating enhanced conformational stability. Fluoxetine showed moderate stabilization (3.56 Å), typical for SSRIs that mainly rely on hydrophobic contacts due to its flexible chain and limited hydrogen bonding. Baicalin_*SLC*_*6*_*A*_*4*_ and chrysoeriol_*SLC*_*6*_*A*_*4*_ showed lower RMSD (3.16 Å and 3.44 Å), while procyanidin B5 had higher RMSD (4.19 Å), suggesting weaker stabilization (Table 6) but still acceptable compared to unbound protein (4.25 Å).

Procyanidin B5, a flavonoid with a non-planar structure, showed higher fluctuations due to fewer π-stacking interactions. The metabolites stabilize SLC6A4 with RMSD values aligning with their structures’ tendency for hydrogen bonding and π–π stacking (Fan et al. 2021). Overall, these metabolites fit in the binding site and restrict conformational motion, indicating receptor stabilization, higher binding affinity, and longer inhibition, supporting the better binding energies of baicalin and chrysoeriol_*SLC*_*6*_*A*_*4*_ (Table 5).

**Table 5:** The Post Dynamic data for the top three metabolites and fluoxetine complexed with SLC6A4

Dynamics	RMSD (Å)	RMSF (Å)	RoG (Å)	SASA (Å)	H-Bonds
*SLC*_*6*_ *A*_*4*_	4.25 ± 0.65	1.70 ± 1.14	24.01 ± 0.15	25131.31 ± 394.91	237.06 ± 11.15
Fluoxetine_*SLC*_*6*_*A*_*4*_	3.56 ± 0.57	1.62 ± 0.83	24.20 ± 0.13	25956.74 ± 554.64	228.89 ± 11.57
Baicalin_*SLC*_*6*_*A*_*4*_	3.16 ± 0.37	1.50 ± 0.67	24.11 ± 0.10	25394.96 ± 501.67	237.04 ± 10.85
Procyanidin B5_ *SLC*_*6*_*A*_*4*_	4.19 ± 0.72	1.79 ± 1.24	24.14 ± 0.14	25629.25 ± 474.52	237.95 ± 11.96
Chrysoeriol_ SALC_6_A_4_	3.44 ± 0.44	1.57 ± 1.19	24.15 ± 0.08	24937.38 ± 485.00	235.40 ± 10.45

### Residue flexibility and active site stabilization

**Fig. 8:**
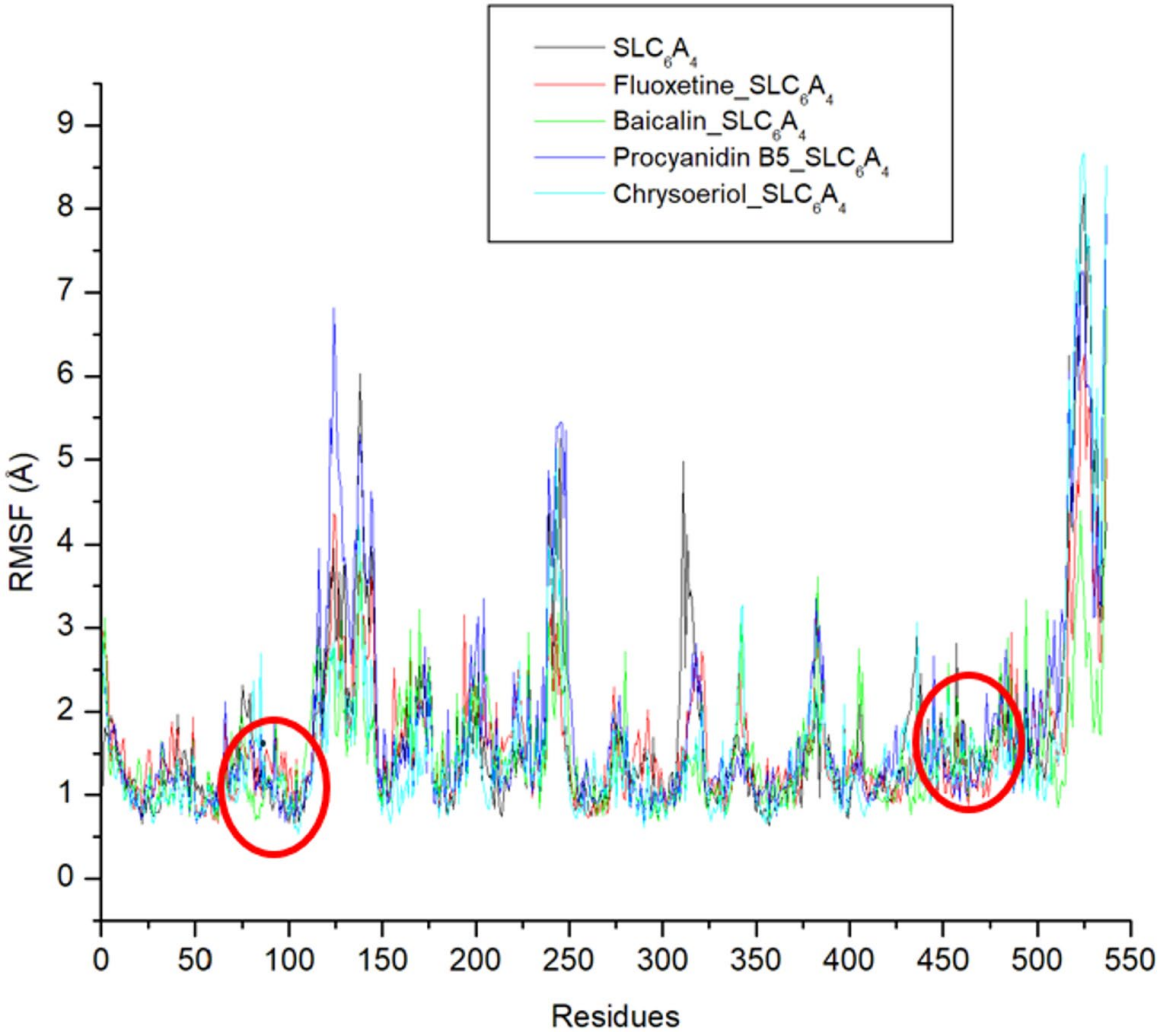
Residue flexibility (RMSF) analysis of *SLC*_*6*_*A*_*4*_–ligand complexes

Residue fluctuation analysis revealed that the baicalin and chrysoeriol-SLC6A4 complex showed significantly reduced fluctuations at key binding residues compared to the fluoxetine and procyanidin B5 complex (Fig. 8), aligning with RMSD and binding free energy data (Tables 4 and 5). Functional proteins like SERT depend on conformational shifts; a strong inhibitor stabilizes in a locked state. The decreased RMSF values (1.50 Å, 1.57 Å) for the baicalin and chrysoeriol complexes suggest they prevent the conformational changes required for serotonin transport, unlike fluoxetine, procyanidin B5, and unbound *SLC*_6_*A*_4_, which showed higher fluctuations (1.62 Å, 1.79 Å, 1.70 Å) (Table 5). Fluoxetine mainly stabilized residues near the binding site, whereas baicalin and chrysoeriol stabilized broader regions, acting as both competitive and allosteric stabilizers (Figs. 5 and 6). Procyanidin B5 exhibited greater fluctuations in loop regions, indicating weaker binding, consistent with the RMSD findings (Fig. 7).

### Hydrogen bonding and binding persistence

**Fig. 9:**
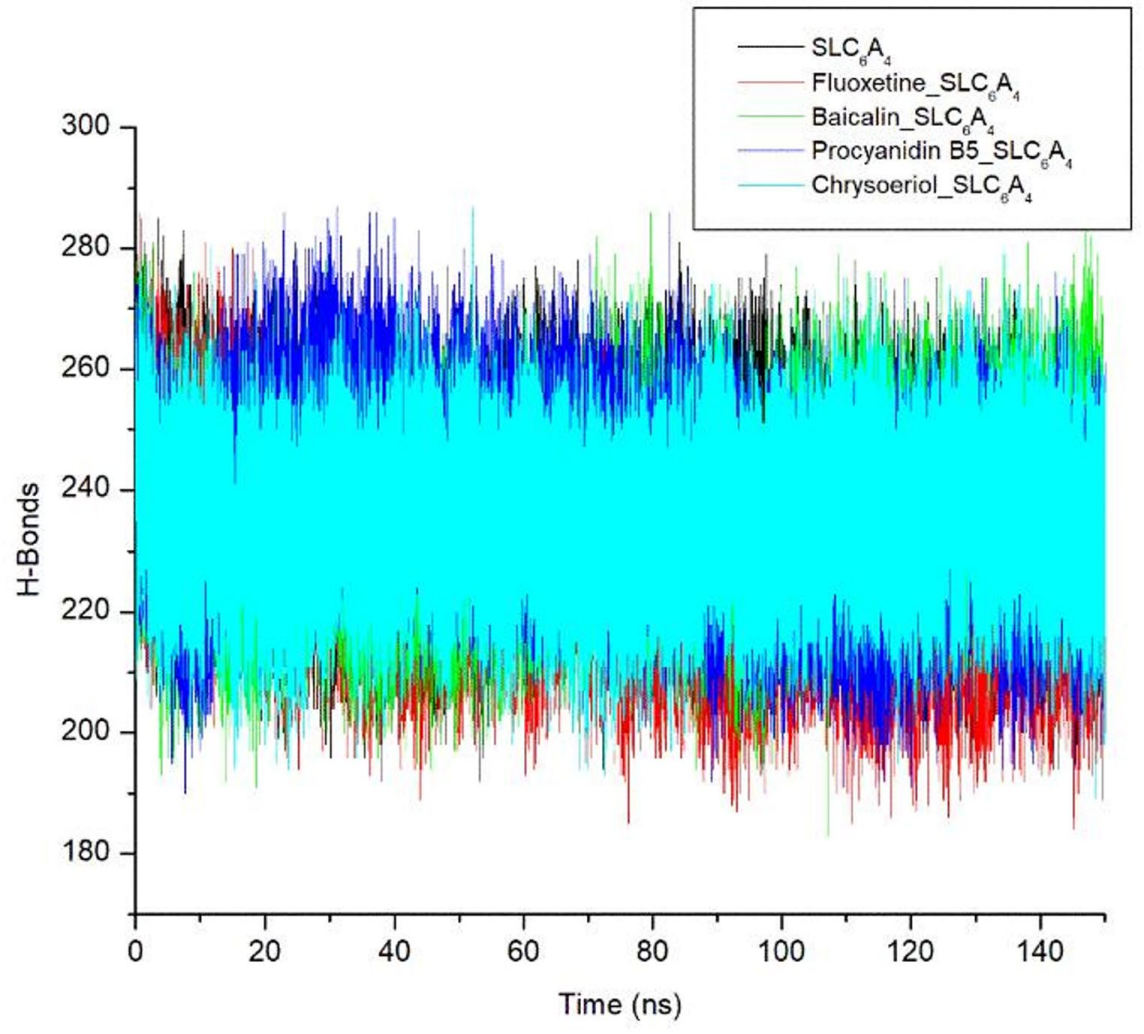
Time-dependent hydrogen bond interactions between *SLC*_*6*_*A*_*4*_ and the top three metabolites

Hydrogen-bonding analysis revealed a key difference between metabolites and the reference drug. Fluoxetine (228) formed few hydrogen bonds due to limited donor/acceptor groups, with hydrophobic interactions dominating. Flavonoids, with multiple hydroxyl groups on a planar aromatic framework, formed many hydrogen bonds (235–237), comparable to the unbound protein (237) (Table 5; Fig. 9). Baicalin consistently formed the most bonds (237), indicating strong residence time within the receptor (Güleç et al. 2025). Chrysoeriol was slightly weaker, likely due to fewer hydrogen bonds. Procyanidin B5 formed more bonds, partly due to TYR95 interactions that help orient serotonin, though these may have been intermittent, as reflected in higher RMSD and RMSF (Joy 2019) (Fig. 6a). Overall, binding persistence depends on the number and duration of hydrogen bonds (Zhang et al. 2024).

### Compactness of the target/resulting complexes

**Fig. 10:**
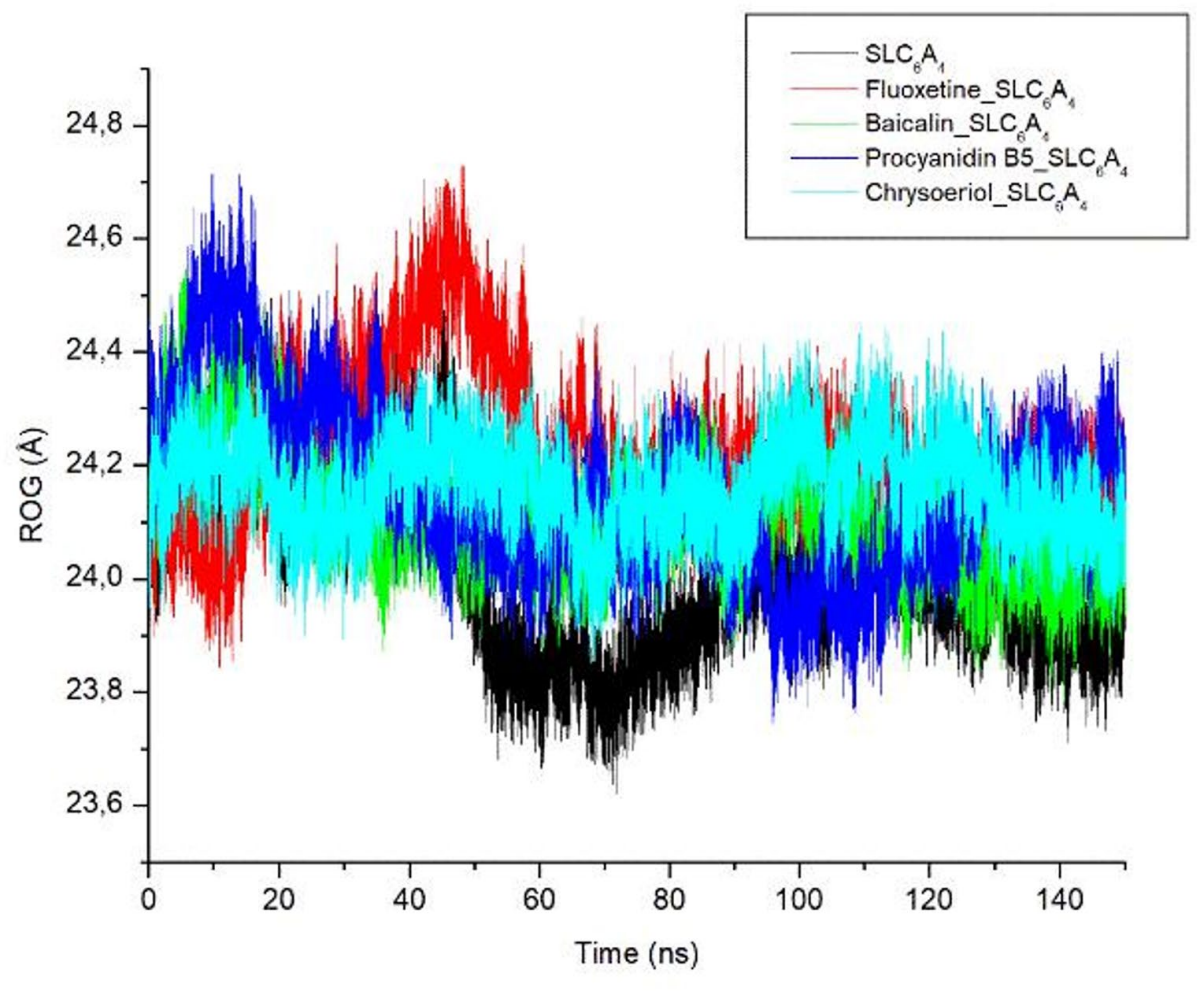
Compactness analysis of protein-ligand structures during simulation. The radius of gyration (Rg) was computed for *SLC*_*6*_*A*_*4*_ to assess the protein’s structural compactness throughout the simulation

The radius of gyration (ROG) reflects the compactness of the protein structure during simulation. A stable ROG indicates that ligand binding does not destabilize the tertiary structure. The top three metabolite complexes maintained nearly constant and similar ROG values (24.11 Å, 24.14 Å, 24.15 Å) (Table 5), indicating that the protein remained tightly folded despite fluctuations in the procyanidin B5 system. In contrast, fluoxetine caused a slight expansion of the protein (24.20 Å) (Fig. 10). This suggests that although fluoxetine binds correctly, it does not maintain the receptor’s structural integrity to the same extent as the flavonoids do. Maintaining structural compactness is particularly important for membrane transport proteins, where conformational shifts regulate substrate transport. By preventing expansion or conformational flexibility, a ligand effectively inhibits transporter activity. Baicalin- and chrysoeriol-bound complexes, however, by maintaining nearly constant ROG values, reflect the preservation of the folded protein architecture, in contrast to the slight expansion observed in the fluoxetine, suggesting relatively weaker structural stabilization. Stable ROG profiles confirm favorable ligand-induced conformational stability.

### Solvent accessibility (SASA)

**Fig. 11:**
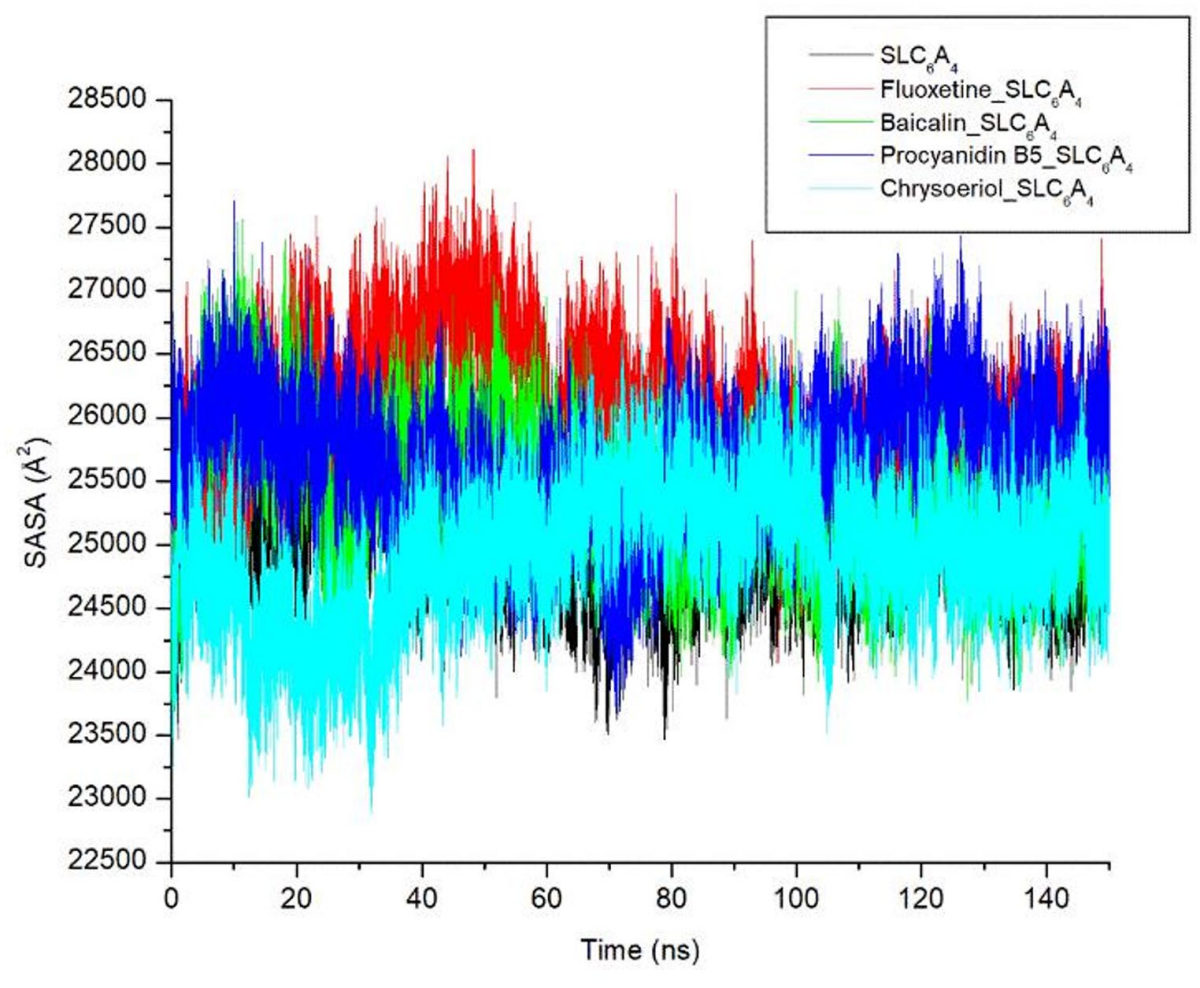
Solvent accessible surface area (SASA) analysis of protein-ligand structures during simulation

SASA analysis revealed lower solvent exposure for the baicalin and chrysoeriol complexes (24937.38 Å and 25394.96 Å, respectively). Lower SASA values indicate deeper insertion of the ligand into the binding cavity. This means the ligand is protected from water molecules and is less likely to dissociate. Fluoxetine (25956.74 Å) remained partially solvent-exposed during the simulation. Such exposure suggests shorter residence time in the binding pocket, which correlates with its reversible inhibition profile observed pharmacologically. However, among the three metabolites, procyanidin B5 (25629.25 Å) exhibited the greatest solvent exposure, which may explain its reduced stability and affinity (Tables 4 and 5; Fig. 11).

### Binding free energy (MM-GBSA) and energy decomposition analysis for *DRD*_4_ and the top three ligands of *Mentha longifolia*

Molecular dynamics simulation analysis of chlorogenic acid, quercetin 3-galactoside, and salvianolic acid A against *DRD*_4_ highlights these compounds’ potential as natural modulators of 6IQL dopamine signalling, similar to fluoxetine’s serotonin-targeting mechanism. The protein-ligand interactions between the three top metabolites, namely chlorogenic acid, quercetin 3-galactoside, and salvianolic acid A, were assessed for their involvement in hydrophobic and hydrophilic forces, such as electrostatic, van der Waals, and hydrophobic interactions upon binding with *DRD*_4_. These interactions form complexes that require atomic-level examination to elucidate recognition patterns and structure-activity relationships (Ferenczy and Kellermayer 2022). The three metabolites had a higher affinity for *the DRD*_4_ binding site than the reference standard (fluoxetine_*DRD*_*4*_ (− 24.91 kcal/mol). Quercetin 3-galactoside**_***DRD*_4_ (− 46.74 kcal/mol), however, has the better affinity compared with chlorogenic acid_*DRD*_4_ (− 32.44 kcal/mol) and salvianolic acid A_*DRD*_4_ (− 37.16 kcal/mol). Quercetin 3-galactoside, salvianolic acid A, and chlorogenic acid showed a fair balance between entropy (water dispersion) and enthalpy (hydrogen bonds, van der Waals forces, electrostatics), attributed to the significant contributions from electrostatic and van der Waals forces, which compensated for the loss due to solvation. Fluoxetine_ *DRD*_4_ complex, on the other hand, was favoured by entropy; however, true binding strength is a tug-of-war between entropy and enthalpy. The insufficient enthalpy from electrostatic and van der Waals interactions to offset the solvation penalty ultimately reduced the binding affinity of fluoxetine for *DRD*_4_. A compound must maintain a balance between entropy and very tight, favourable contacts. Despite the robust contribution from electrostatic energy, the solvation energy imposes a greater penalty, significantly reducing the overall binding free energy of fluoxetine for *DRD*_4_ (Table 6).

**Fig. 12:**
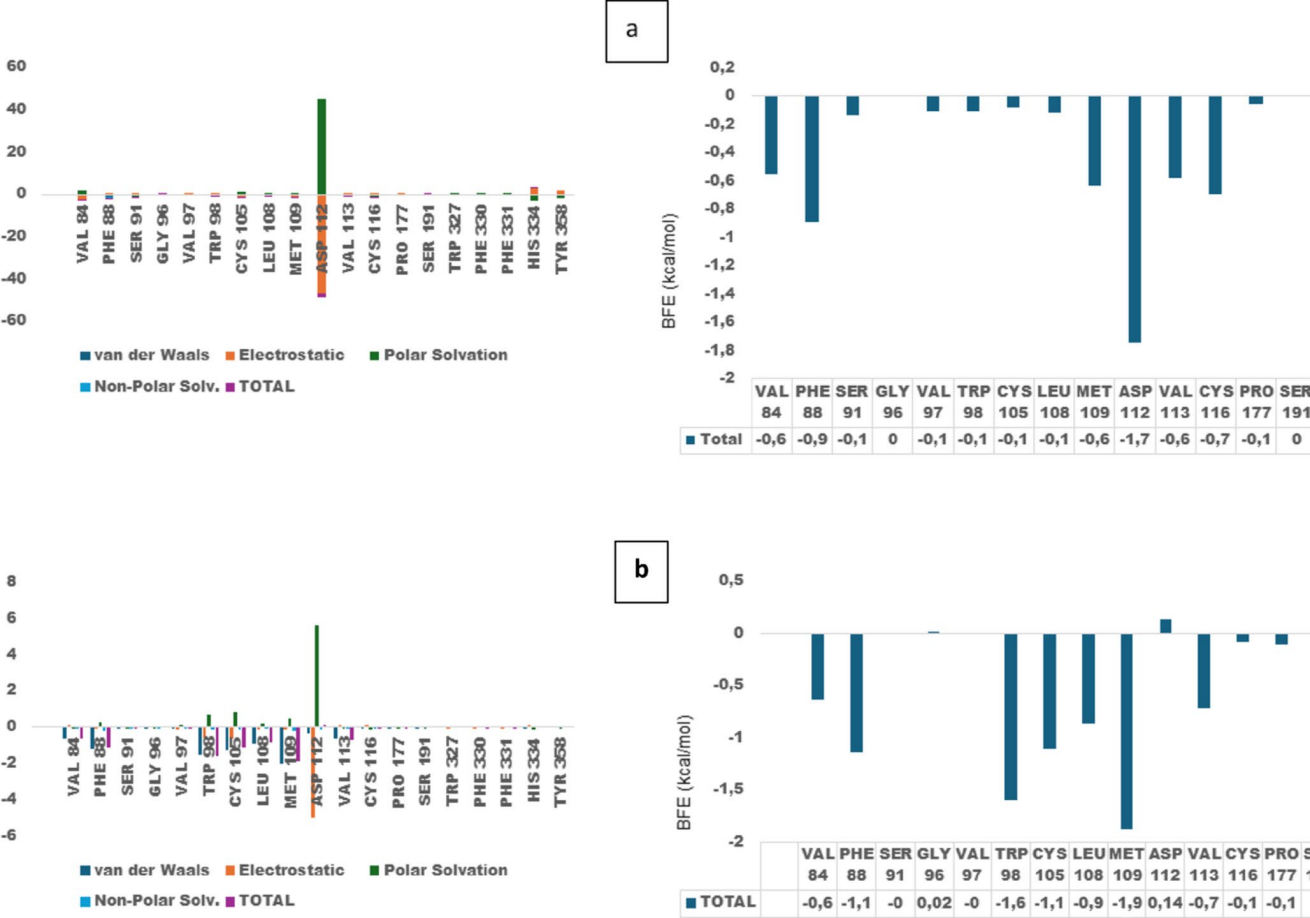
Per residue decomposition for **a** Fluoxetine_*DRD*_4_ and **b** Chlorogenic Acid_*DRD*_4_

**Fig. 13:**
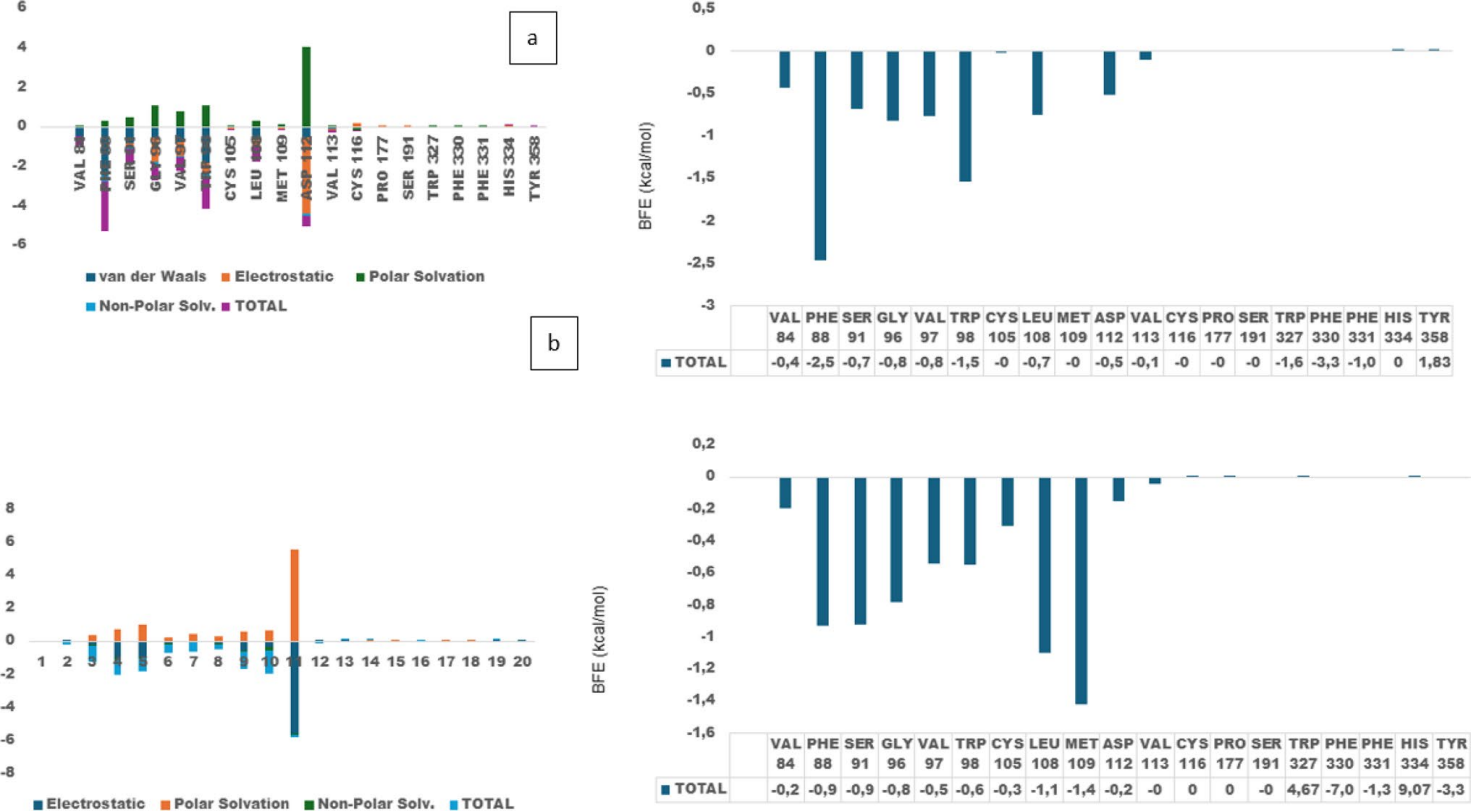
Per residue decomposition for **a** Quercetin 3-galactoside_*DRD*_4_ and **b** Salvianolic acid A_*DRD*_4_

The binding free energy of the interactions involving chlorogenic acid_*DRD*_4_, quercetin 3-galactoside_*DRD*_4_, salvianolic acid A_*DRD*_4_, and fluoxetine**_***DRD*_4_ was further broken down into contributions from individual amino acids to identify key residues involved in ligand binding. In the chlorogenic acid_*DRD*_4_ complex, 19 residues of the protein contacted the ligand at 50 ns and 100 ns, while 20 did so at 150 ns (Fig. S13). Among these, only MET109 (− 1.9 kcal/mol) contributed significantly to the total binding energy (Fig. 12b). Quercetin 3-galactoside_*DRD*_4_ also made a notable energy contribution through PHE88 (− 2.5 kcal/mol), with 19 interactions at 50 ns, increasing to 21 at 100 ns, and 22 at 150 ns (Figs. 13a, S14). This shows an increased interaction with the important amino acid residues over the simulation period. This is important because of its cumulative effect, which results in the highest average binding free energy (Joy 2019) (Fig. 13b). MET109 contributed most significantly to the binding energy of salvianolic acid A_*DRD*_4_ (− 1.4 kcal/mol), forming 17 interactions at 50 ns, 18 at 100 ns, and 17 at 150 ns (Fig. S15). Conversely, fluoxetine_*DRD*_4_ contributed the most through Asp112 (− 1.7 kcal/mol), forming 15 interactions at 50 ns, then increasing to 16 at 100 ns, and remaining at 15 at 150 ns (Figs. 12a, S16).

**Table 6:** The energy component details for the top three metabolites and fluoxetine complexed with *DRD*_4_

	Energy components (kcal/mol)				
	ΔE_vdW_	ΔE_elec_	ΔG_gas_	ΔG_solv_	ΔG_bind_
Fluoxetine_*DRD*_4_	− 29.80 ± 3.22	− 98.34 ± 15.26	− 128.15 ± 15.24	103.24 ± 13.54	− 24.91 ± 3.75
Chlorogenic acid_*DRD*_4_	− 43.50 ± 3.92	− 21.11 ± 11.89	− 64.61 ± 10.64	32.17 ± 7.87	− 32.44 ± 4.83
Quercetin 3-galactoside_*DRD*_4_	− 43.54 ± 6.21	− 58.12 ± 21.28	− 101.67 ± 17.33	54.92 ± 11.85	− 46.74 ± 6.90
Salvianolic acid A_*DRD*_4_	− 41.92 ± 7.53	− 47.38 ± 17.99	− 89.30 ± 18.47	52.14 ± 9.88	− 37.16 ± 10.17

### Structural stability of complexes

**Fig. 14:**
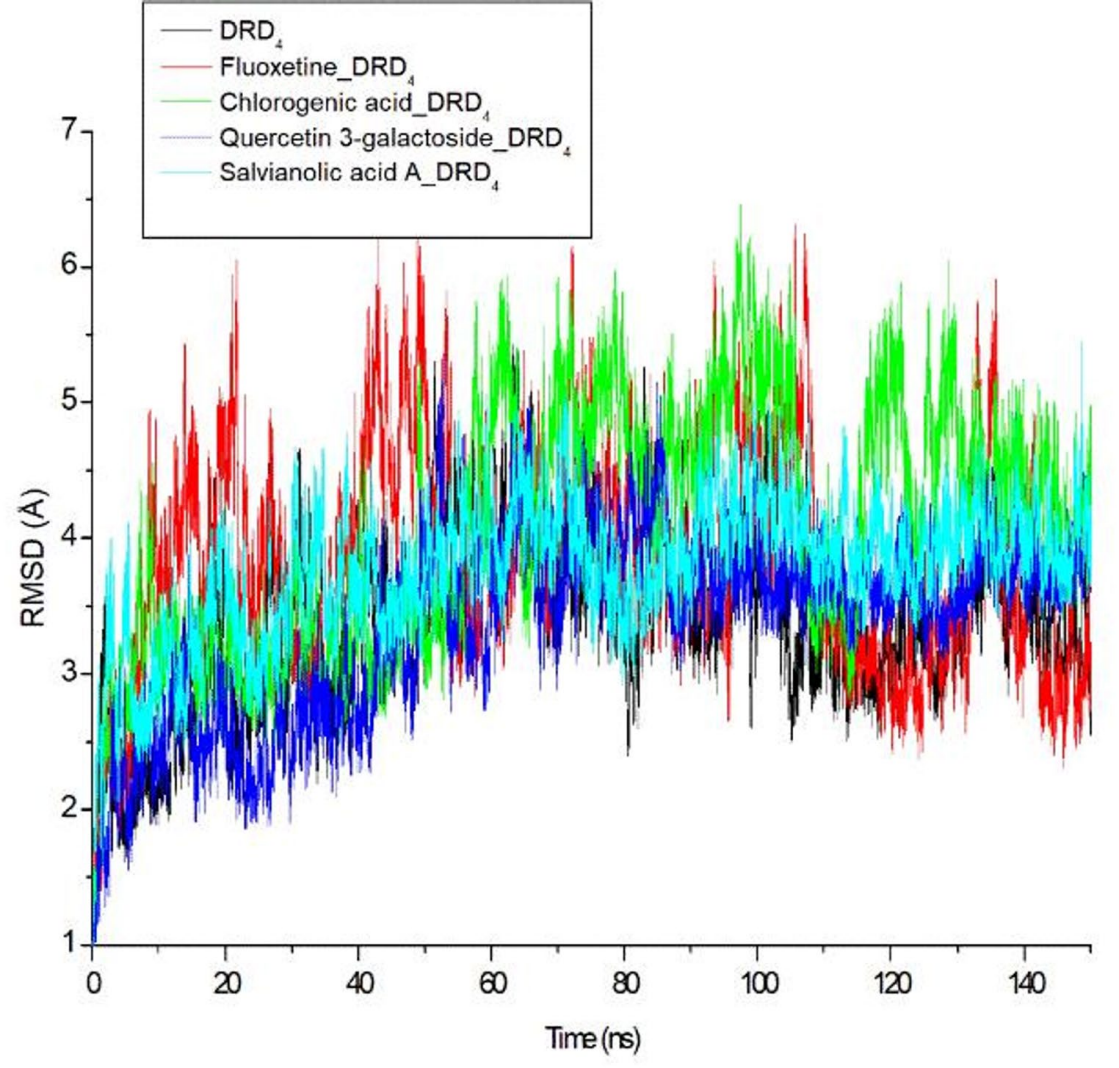
Backbone RMSD profile of *DRD*_*4*_–ligand complexes during 150 ns molecular dynamics simulation

Before further analysis, it is crucial to assess the stability of the simulated complexes to ensure the reliability of the data obtained. We calculated the average RMSD values from MD trajectories lasting 150 ns and displayed the results in Fig. 14. The mean RMSD values for chlorogenic acid_*DRD*_*4*_, quercetin 3-galactoside_*DRD*_*4*_, salvianolic acid A_*DRD*_*4*_, and fluoxetine_*DRD*_*4*_ were 4.06 Å, 3.38 Å, 3.77 Å, and 3.79 Å, respectively, relative to the unbound protein (3.41 Å). The RMSD profiles indicated that all complexes reached equilibrium within the first 10 ns of the simulation, confirming proper equilibration of the systems (Fig. 14). However, notable differences among the ligands were observed starting at 20 ns of simulation. Quercetin 3-galactoside_ *DRD*_*4*_ was the most stable of the complexes compared to the unbound protein. Salvianolic acid A_*DRD*_*4*_, on the other hand, competes well with the reference standard, fluoxetine_*DRD*_*4*_ complex (Table 6; Fig. 14). Chlorogenic acid, a phenolic compound, on the other hand, despite being known for its neuroprotective activity (Naveed et al. 2018), when bound to *DRD*_*4*_ was the least stable of the three metabolites, likely due to fluctuations between 60 ns that persist until the end of the simulation, thereby increasing the overall RMSD (4.06 Å). Moreover, this observation has been reported for chlorogenic acid with other antidepressant targets, showing greater fluctuations at the beginning of the simulation but stabilizing later towards the end. This also corroborates the need for longer simulations to capture a complete perception of the simulation (Barsalou 2009).

Quercetin 3-galactoside, a known quercetin in apples, exhibits strong stabilisation, consistent with quercetin’s role in reducing depression-like behavioural dysfunction (Wang et al. 2021). Its activity hinges on two functional groups behaving differently within a protein-binding site (Zheng et al. 2003). The flat flavanol ring, comprising a phenolic catechol ring (3′,4′ di-OH) and heterocyclic chromen-4-one, is rigid, planar, π-electron-rich, and hydrophobic (Zheng et al. 2003). This allows the molecule to interact: one side enters the protein pocket, the other interacts with surface residues and solvent. Stabilization mainly occurs through delocalized π electrons creating electrostatic interactions with aromatic residues, and hydrogen bonding via phenolic –OH groups, sugar –OH groups, a carbonyl, and ether oxygen. During simulation, hydrogen bonds persist, especially around 50 ns, with hydrophobicity aiding entry into active pockets, cavities, and grooves to avoid water (Fig. 14; Table 6). The sugar tail and phenolic OH groups reform hydrogen bonds continuously, resulting in more stable and effective binding of the quercetin 3-galactoside-DRD4 complex (Ferenczy and Kellermayer 2022).

The greater electrostatic contribution to binding free energy indicates that a balance of enthalpy and entropy drives flavonoid-protein interactions. Fluoxetine gains entropy from water displacement, adding a penalty to its binding. It is a flexible SSRI with limited hydrogen bonding. Quercetin-3-galactoside’s superior docking and MD stability arise from its polyhydroxylated scaffold, which enables hydrogen bonding and π–π interactions. The stability in MD trajectories highlights quercetin’s potential for neuropsychiatric drug development. (Table 7).

**Table 7:** The post dynamic data for the top three metabolites and fluoxetine complexed with DRD4

Dynamics	RMSD (Å)	RMSF (Å)	RoG (Å)	SASA (Å)	H-Bonds
*DRD*_*4*_	3.41 ± 0.63	2.35 ± 0.89	28.22 ± 0.35	18998.72 ± 472.28	167.76 ± 9.02
Fluoxetine_*DRD*_*4*_	3.79 ± 0.80	2.17 ± 0.96	29.13 ± 0.33	19908.92 ± 358.05	165.47 ± 10.25
Chlorogenic acid_ *DRD*_*4*_	4.06 ± 0.89	2.75 ± 1.19	28.36 ± 0.34	19814.31 ± 436.70	162.03 ± 9.09
Quercetin 3-galactoside_*DRD*_*4*_	3.38 ± 0.67	2.37 ± 1.08	28.44 ± 0.36	19762.66 ± 342.77	162.25 ± 9.10
Salvianolic acid A_ *DRD*_*4*_	3.77 ± 0.49	2.28 ± 1.05	28.48 ± 0.32	19564.68 ± 386.85	157.86 ± 8.85

### Residue flexibility and active site stabilization

**Fig. 15:**
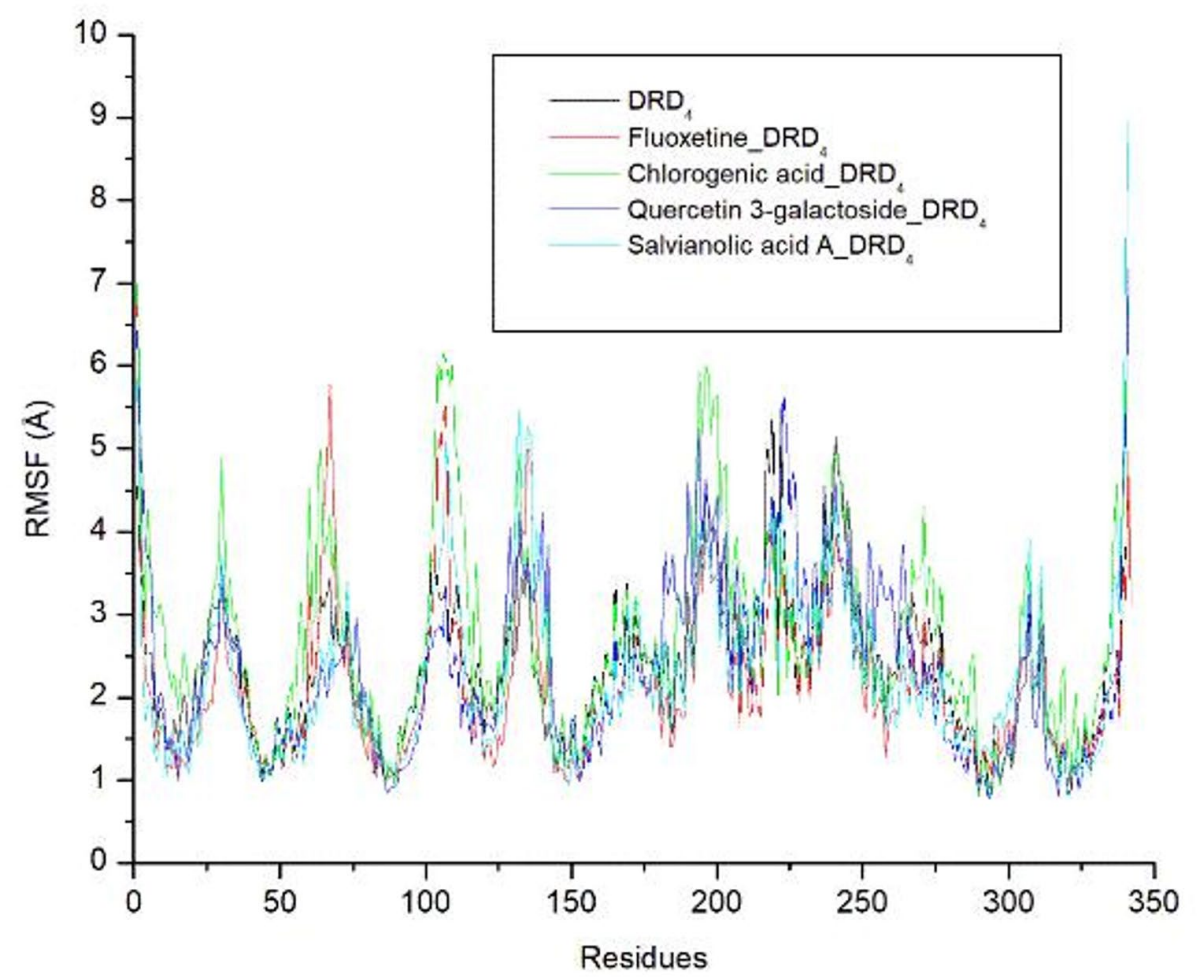
Residue flexibility (RMSF) analysis of *DRD*_*4*_–ligand complexes. Root-mean-square fluctuation (RMSF) values of amino acid residues were calculated to assess local flexibility of *DRD*_*4*_ in the presence of the tested ligands

Residue fluctuation analysis revealed the local flexibility of amino acids at the binding site. Quercetin 3-galactoside_ DRD4 (2.37 Å) and salvianolic acid A_ DRD4 (2.28 Å) showed significantly reduced fluctuations around key binding residues compared to the unbound protein (2.35 Å), yet remained more flexible than fluoxetine (2.17 Å) (Table 7; Fig. 15). This aligns with the RMSD values recorded (Table 7). Conversely, the quercetin 3-galactoside_ DRD4 complex had a slightly higher fluctuation (2.37 Å) than the unbound protein, suggesting it may restrict amino acid flexibility, with the 2 Å difference possibly due to minimal fluctuations around residues 200–250. Chlorogenic acid_DRD4 (2.75 Å) showed greater fluctuations, indicating weaker binding and intermittent interactions, consistent with its low entropy and enthalpy contributions, and resulted in the lowest binding free energy among the three metabolites and the highest RMSD (Fig. 14). The entropy gain from fluoxetine binding likely reduced RMSF due to water displacement, not strong interactions. While water displacement can stabilize the drug, it also increases rigidity, limiting movement and possibly decreasing protein fluctuations without stronger binding. This is supported by the RMSD and ROG values of fluoxetine_DRD4 compared to quercetin 3-galactoside_DRD4 and salvianolic acid A_DRD4.

### Hydrogen bonding and binding persistence

**Fig. 16:**
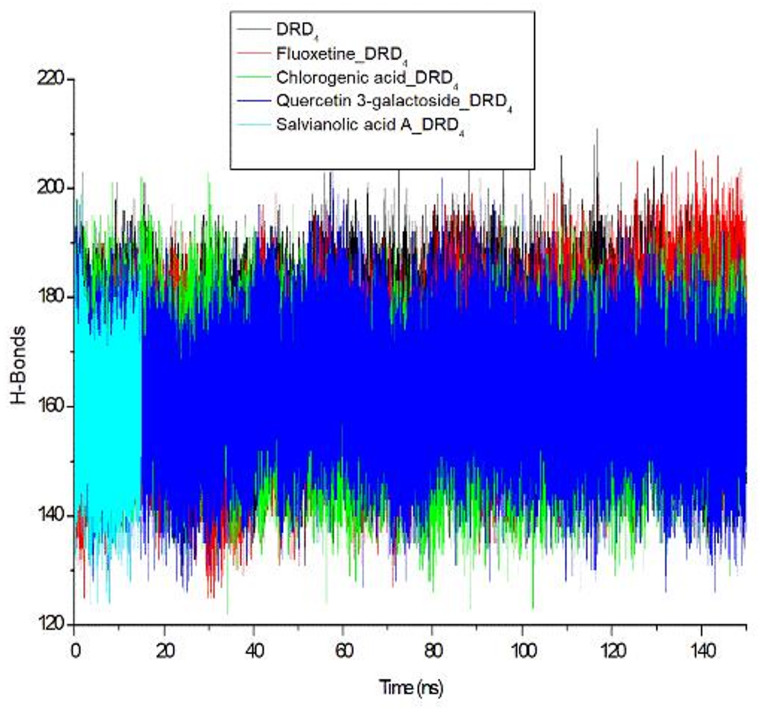
Time-dependent hydrogen bond interactions between *DRD*_*4*__top three metabolites. The number of intermolecular hydrogen bonds formed between each ligand and the active-site residues in *DRD*_*4*_ was calculated throughout the trajectory

All the top metabolites, namely quercetin 3-galactoside_ *DRD*_*4*_ (162), salvianolic acid A_*DRD*_*4*_ (162), and chlorogenic acid_ *DRD*_*4*_ (157), including the reference drug (165), exhibited fewer hydrogen bonds than the unbound protein (167), highlighting a key difference between the metabolites and the unbound protein (Table 7). This likely occurs because proteins are built on a backbone scaffold of α-helices and β-sheet strands, in which the numbers of hydrogen-bond donors and acceptors are perfectly balanced. Consequently, proteins without ligands compensate for the lack of binding stabilization by forming more intramolecular hydrogen-bond networks (Keiser et al. 2007). Therefore, when a metabolite binds to these residues, proteins naturally orient toward the ligand, thereby decreasing the total number of hydrogen bonds formed (Ferenczy and Kellermayer 2022). The new interactions show decreased flexibility and more conformational stabilization, not loss of stability (Fig. 16). This aligns with thermodynamic principles where enthalpic gains from specific contacts offset entropy loss due to structural ordering, as seen with quercetin 3-galactoside, salvianolic acid A, and chlorogenic acid_DRD4 complexes, which exhibited better binding energy, stability, and lower fluctuation (Tzeng and Kalodimos 2011).

The fluoxetine_*DRD*_*4*_ complex showed higher hydrogen-bonding values than other compounds. While hydrogen bonding stabilises solvation free energy, it doesn’t always lead to stronger binding due to desolvation penalties during transfer from the solvent to protein-binding sites. This was evident in the binding free energy and post-dynamics parameters for the fluoxetine_*DRD*_*4*_ complex (Tzeng and Kalodimos 2011).

### Compactness of the target/complex

**Fig. 17:**
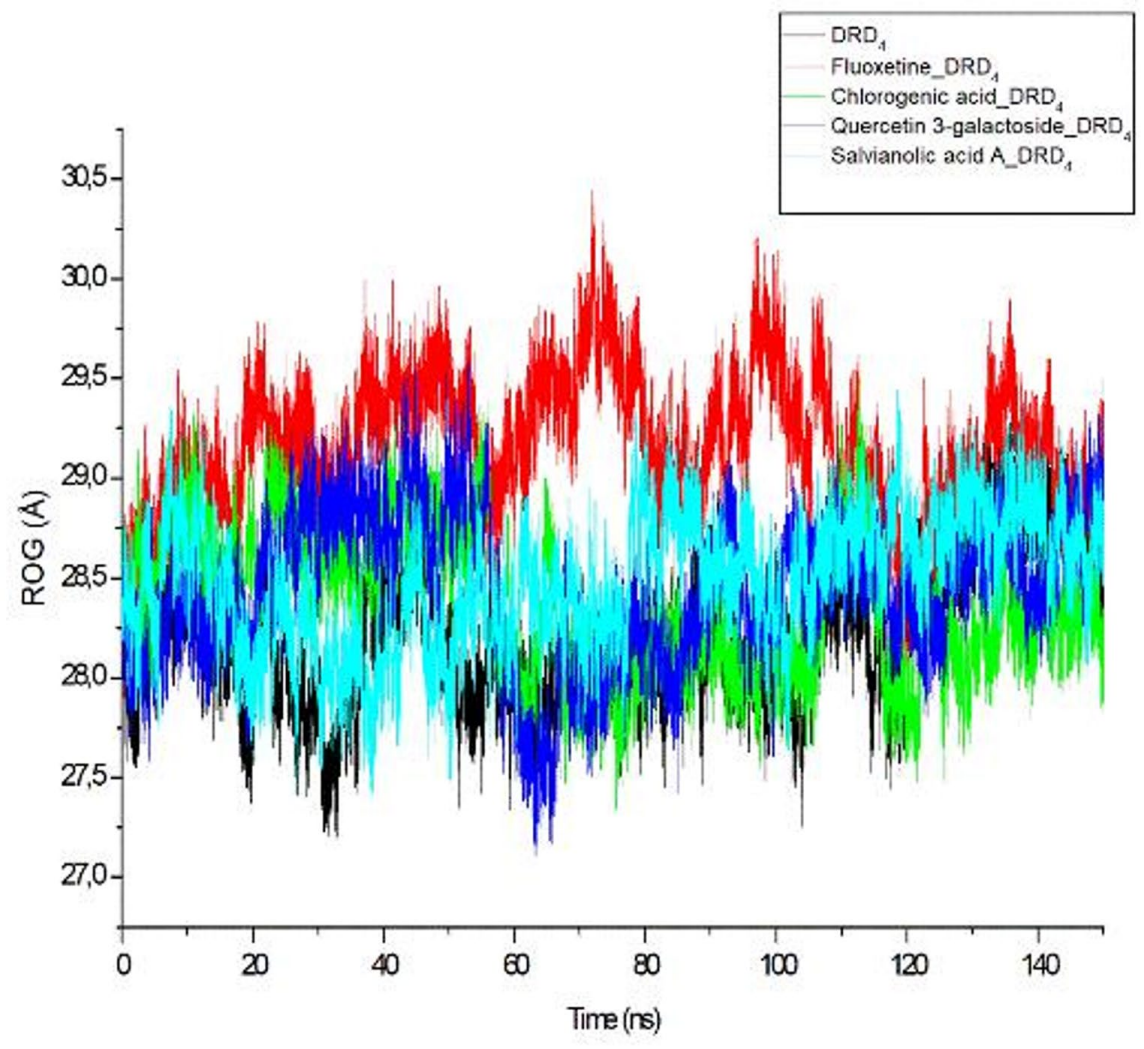
Compactness analysis of protein-ligand structures during simulation. The radius of gyration (ROG) was computed for *DRD*_*4*_ to assess the protein’s structural compactness throughout the simulation

The radius of gyration (ROG) reflects the protein’s compactness during simulation. A stable ROG indicates that ligand binding does not destabilize the tertiary structure of the protein. Evidently, there was only a marginal difference between the ROG values of the top metabolites, all at around 28 Å (Table 7; Fig. 17). This indicates that the protein remained tightly folded, confirming the RMSF and RMSD values. In contrast, fluoxetine caused a slight expansion of the protein (29.13 Å). This suggests that although fluoxetine binds correctly, it does not maintain the receptor’s structural integrity to the same extent as quercetin 3-galactoside, salvianolic acid A, and chlorogenic acid_*DRD*_*4*_.

### Solvent accessibility (SASA)

**Fig. 18:**
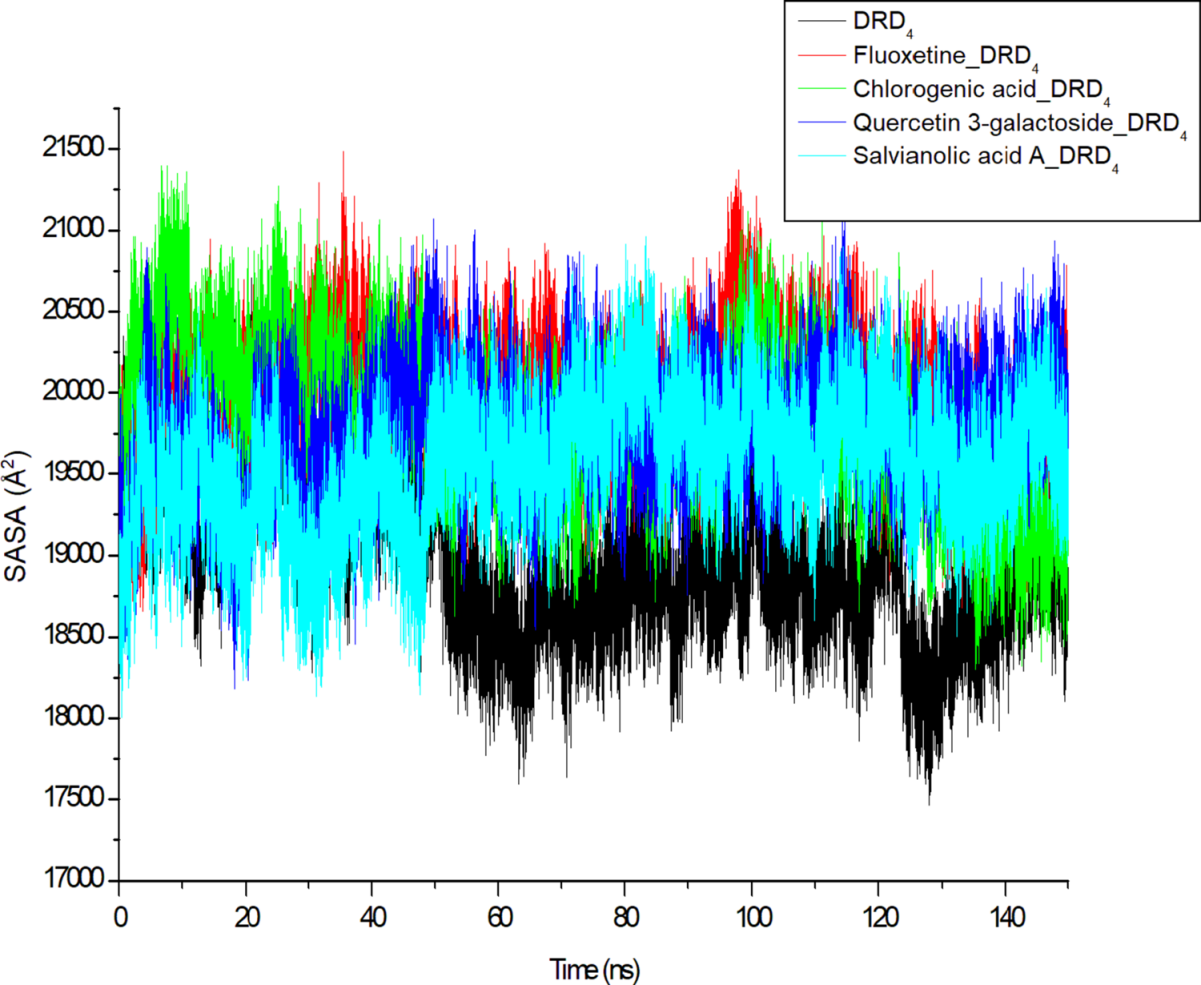
Solvent Accessible Surface Area (SASA) analysis of protein-ligand structures during simulation

Quercetin 3-galactoside_ *DRD*_*4*_ (19762.66 Å), salvianolic acid A_ *DRD*_*4*_ (19564.68 Å), and chlorogenic acid_*DRD*_*4*_ (19814.31 Å), including the reference drug (19908.92 Å), exhibited a higher SASA than the unbound protein (18998.72 Å). However, according to the induced-fit mechanism proposed by Koshland Jr (1995), ligand binding to a protein can induce conformational changes in the protein. This means some ligands induce loop rearrangement, active-site widening, and domain movement. The pocket expands, certain buried residues become partially exposed, and the overall solvent-accessible area increases (Koshland Jr 1995). The higher SASA values observed for the protein–metabolite complexes relative to the apo protein suggest increased solvent exposure upon ligand binding. This may arise from induced-fit conformational rearrangements, partial solvent exposure of the bound metabolite, and structural expansion of active-site regions. Importantly, the reduced RMSD and RMSF values indicate that the complexes remain structurally stable despite increased solvent accessibility. Therefore, the elevated SASA reflects conformational adaptation rather than destabilization (Table 7; Fig. 18).

## Conclusion

In this study, we demonstrated the potential of *L. leonurus* and *M. longifolia* as promising medicinal plants for further exploration in depression. Using a network pharmacology approach integrated with molecular docking and MD simulation, key depression-related targets and pathways were identified, including dopaminergic and serotonergic synapses, neuroactive ligand–receptor interactions, and *PI*_3_*K/Akt* signalling. *Leonotis leonurus* compounds such as procyanidin B5, chrysoeriol, and baicalin showed strong interactions with targets such as *SLC*_6_*A*_4_, *PTGS*_2_, *MAOA*, *AKT*_1_, and *GSK*_3_*β*, supporting their potential in modulating neurotransmission and inflammatory responses. Similarly in *M. longifolia*, compounds such as salvianolic acid A, rosmarinic acid, and hesperetin exhibited higher binding ability than fluoxetine when docked to *DRD*_4_, *DRD*_*3*_, and *GSK*_3_*β*, indicating they may have potential therapeutic significant comparable to conventional antidepressants.

Fluoxetine functions by blocking serotonin reuptake through inhibition of the serotonin transporter, while the MD simulation results suggest that baicalin, chrysoeriol, procyanidin B, quercetin 3-galactoside, salvianolic acid A, and chlorogenic acid may interact with the transporter in a similar but potentially stronger manner. The molecular dynamics simulation corroborated the docking predictions and demonstrated that the selected plant metabolites formed stable, energetically favourable complexes with the studied proteins. Among the tested compounds, baicalin and quercetin 3-galactoside exhibited the highest binding stability and affinity, suggesting they are the most promising. This is because flavonoids are known to influence monoaminergic neurotransmission, including serotonin pathways. The simulation findings support this pharmacological behavior at the molecular level. The ability to stabilize the transporter conformations (*SLC*_6_*A*_4_ and *DRD*_*4*_) and to maintain persistent binding suggests that these metabolites could potentially be natural serotonin reuptake inhibitors or transporter modulators. Additionally, their multi-target interactions with both *SLC*_6_*A*_4_ and *DRD*_*4*_ suggest polypharmacology, a desirable feature in neurological disorders, in which multiple pathways contribute to disease progression. However, Baicalin and procyanidin B5 exhibit extremely low oral bioavailability, often reported to be between 2.2% and 5% in animal models. This is attributed to its low aqueous solubility (16.82) and poor membrane permeability due to its glycosylated structure. This limitation can, however, be mitigated by subjecting these two to a structural enhancement or nanoformulations. Therefore, we propose that naturally occurring flavonoids may represent potential scaffolds for the development of novel serotonin transporter inhibitors or adjunct antidepressant agents. Together, these findings provide compelling evidence that both plants have the potential to exert antidepressant effects through multi-component, multi-target mechanisms involving neurotransmitter regulation, neuroplasticity, and immune modulation. While further experimental and clinical validation is necessary, this study establishes a strong pharmacological basis for the traditional use of *L*. *leonurus* and *M*. *longifolia* in mental health and supports their potential development as alternative or adjunctive therapies for depression.

## Electronic Supplementary Material

Below is the link to the electronic supplementary material.

Supplementary Material 1

## Data Availability

Supporting data are presented as part of the supplementary files associated with this submission.

## References

[CR1] Abdelhamid RE, Kovács KJ, Nunez MG, Larson AA (2014) Depressive behavior in the forced swim test can be induced by TRPV1 receptor activity and is dependent on NMDA receptors. Pharmacol Res 79:21–27 24200896 10.1016/j.phrs.2013.10.006 PMC3947229

[CR2] Adams R, Burnley RJ, Valenzano CR, Qureshi O, Doyle C, Lumb S, Del Carmen Lopez M, Griffin R, McMillan D, Taylor RD, Meier C, Mori P, Griffin LM, Wernery U, Kinne J, Rapecki S, Baker TS, Lawson AD, Wright M, Ettorre A (2017) Discovery of a junctional epitope antibody that stabilizes IL-6 and gp80 protein:protein interaction and modulates its downstream signaling. Sci Rep 7:37716 28134246 10.1038/srep37716 PMC5278397

[CR3] Aktar A, Bhuia S, Chowdhury R, Ferdous J, Khatun M, Hasan SA, Mia E, Hasan R, Islam MT (2024) An insight of plant source, toxicological profile, and pharmacological activities of iridoid loganic acid: a comprehensive review. Chem Biodivers 21:e202400874 39113595 10.1002/cbdv.202400874

[CR4] Al-Khatib Y, Akhtar MA, Kanawati MA, Mucheke R, Mahfouz M, Al-Nufoury M (2022) Depression and metabolic syndrome: a narrative review. Cureus 14:2168–8184 10.7759/cureus.22153 PMC8920832 35308733

[CR5] Al-Taie SSK, Al-Kenane NFM (2020) Study biochemistry of *Mentha longifolia* (L.) Huds.: a review. Herbs Spices :27

[CR6] Alam MA, Zaidul ISM, Ghafoor K, Sahena F, Hakim MA, Rafii MY, Abir HM, Bostanudin MF, Perumal V, Khatib A (2017) *In vitro* antioxidant and, α-glucosidase inhibitory activities and comprehensive metabolite profiling of methanol extract and its fractions from *Clinacanthus nutans*. BMC Complement Altern Med 17:181 28359331 10.1186/s12906-017-1684-5 PMC5374668

[CR7] Ali DH, Hegazy HG, Ali EHA, El-Tantawi H (2025) *Ginkgo biloba* L. leaf extract (EGb 761) alleviates reserpine-induced depression-like symptoms in aged rats by enhancing serotonin/norepinephrine levels and reducing oxidative/nitrosative stress. Naunyn Schmiedebergs Arch Pharmacol 398:12115–12131 40100376 10.1007/s00210-025-03972-9 PMC12449422

[CR8] Anwar F, Abbas A, Mehmood T, Gilani AH, Rehman, Nu (2019) Mentha: a genus rich in vital nutra-pharmaceuticals: a review. Phytother Res 33:2548–2570 31286590 10.1002/ptr.6423

[CR9] Barsalou LW (2009) Simulation, situated conceptualization, and prediction. Philos Trans Royal Soc B: Biol Sci 364:1281–1289 10.1098/rstb.2008.0319 PMC2666716 19528009

[CR10] Bi Y, Ren D, Guo Z, Ma G, Xu F, Chen Z, An L, Zhang N, Ji L, Yuan F, Liu L, Hou B, Yang F, Yu S, Yi Z, Xu Y, He L, Sun X, Dong Z, He G (2020) DRDInfluence and interaction of genetic, cognitive, neuroendocrine and personalistic markers to antidepressant response in Chinese patients with major depression. Prog Neuropsychopharmacol Biol Psychiatry 104:110036 32702381 10.1016/j.pnpbp.2020.110036

[CR11] Bonokwane MB, Lekhooa M, Struwig M, Aremu AO (2022) Antidepressant effects of South African plants: an appraisal of ethnobotanical surveys, ethnopharmacological and phytochemical studies. Front Pharmacol 13:895286 35846999 10.3389/fphar.2022.895286 PMC9277359

[CR12] Brändén G, Sjögren T, Schnecke V, Xue Y (2014) Structure-based ligand design to overcome CYP inhibition in drug discovery projects. Drug Discov Today 19:905–911 24642031 10.1016/j.drudis.2014.03.012

[CR13] Buza A, Türkeş C, Arslan M, Demir Y, Dincer B, Nixha AR, Beydemir Ş (2024) Novel benzenesulfonamides containing a dual triazole moiety with selective carbonic anhydrase inhibition and anticancer activity. Res Rev Articles Med Chem 16:324–345 10.1039/d4md00617h PMC11525713 39493223

[CR14] Case DA, Aktulga HM, Belfon K, Ben-Shalom IY, Berryman JT, Brozell SR, Cerutti DS, Cheatham TE III, Cisneros GA, Cruzeiro VWD (2023) Amber 2023. University of California, San Francisco, USA

[CR15] Chang Y-H, Lee S-Y, Chen S-L, Tzeng N-S, Wang T-Y, Lee IH, Chen PS, Huang S-Y, Yang YK, Ko H-C (2013) Genetic variants of the BDNF and DRD3 genes in bipolar disorder comorbid with anxiety disorder. J Affect Disord 151:967–972 24021960 10.1016/j.jad.2013.08.017

[CR18] Chen N, Shu B-C (2006) Dopamine D4 receptor gene and the– 521C > T polymorphism of the upstream region of the dopamine D4 receptor gene in schizophrenia. Psychiatr Genet 16:139–143 16829780 10.1097/01.ypg.0000199446.54420.ff

[CR16] Chen J, Wang M, Waheed Khan RA, He K, Wang Q, Li Z, Shen J, Song Z, Li W, Wen Z, Jiang Y, Xu Y, Shi Y, Ji W (2015) The *GSK3B* gene confers risk for both major depressive disorder and schizophrenia in the Han Chinese population. J Affect Disord 185:149–155 26186530 10.1016/j.jad.2015.06.040

[CR19] Chen Z-y, Xie D-f, Liu Z-y, Zhong Y-q, Zeng J-y, Chen Z, Chen X-l (2020) Identification of the significant pathways of *Banxia Houpu* decoction in the treatment of depression based on network pharmacology. PLoS ONE 15:e0239843 32997725 10.1371/journal.pone.0239843 PMC7527207

[CR17] Chen L, Cao H, Huang Q, Xiao J, Teng H (2022) Absorption, metabolism and bioavailability of flavonoids: a review. Crit Rev Food Sci Nutr 62:7730–7742 34078189 10.1080/10408398.2021.1917508

[CR20] Choudhary M, Shaikh M, Wahab A-T, Rahman A (2020) In silico identification of potential inhibitors of key SARS-CoV-2 3CL hydrolase (Mpro) via molecular docking, MMGBSA predictive binding energy calculations, and molecular dynamics simulation. PLoS ONE 15:e0235030 32706783 10.1371/journal.pone.0235030 PMC7380638

[CR21] Choy KW, Murugan D, Leong X-F, Abas R, Alias A, Mustafa MR (2019) Flavonoids as natural anti-inflammatory agents targeting nuclear factor-kappa B (NFκB) signaling in cardiovascular diseases: a mini review. Front Pharmacol 10:1295 31749703 10.3389/fphar.2019.01295 PMC6842955

[CR120] Coleman JA, Green EM, Gouaux E (2016) X-ray structures andmechanism of the human serotonin transporter. Nature 532:334-339 10.1038/nature17629 10.1038/nature17629 PMC4898786 27049939

[CR22] Dannlowski U, Domschke K, Birosova E, Lawford B, Young R, Voisey J, Morris CP, Suslow T, Konrad C, Kugel H, Ohrmann P, Bauer J, Schöning S, Zavorotnyy M, Diemer J, Arolt V, Baune BT, Zwanzger P (2013) Dopamine D3 receptor gene variation: impact on electroconvulsive therapy response and ventral striatum responsiveness in depression. Int J Neuropsychopharmacol 16:1443–1459 22093107 10.1017/S1461145711001659

[CR23] De Lima EP, Laurindo LF, Catharin VC, Direito R, Tanaka M, Jasmin Santos German I, Lamas CB, Guiguer EL, Araújo AC, Fiorini AM, Barbalho SM (2025) Polyphenols, alkaloids, and terpenoids against neurodegeneration: evaluating the neuroprotective effects of phytocompounds through a comprehensive review of the current evidence. Metabolites. 10.3390/metabo15020124 39997749 10.3390/metabo15020124 PMC11857241

[CR24] Dennis G, Sherman BT, Hosack DA, Yang J, Gao W, Lane HC, Lempicki RA (2003) DAVID: database for annotation, visualization, and integrated discovery. Genome Biol 4:1–1112734009

[CR25] Diniz BS, Talib LL, Giroud Joaquim HP, de Paula VRJ, Gattaz WF, Forlenza OV (2011) Platelet *GSK3B* activity in patients with late-life depression: marker of depressive episode severity and cognitive impairment? World J Biol Psychiatry 12:216–222 21314327 10.3109/15622975.2010.551408

[CR26] Dong C, Wong ML, Licinio J (2009) Sequence variations of *ABCB1*, *SLC6A2*, *SLC6A3*, *SLC6A4*, *CREB1*, *CRHR1* and *NTRK2*: association with major depression and antidepressant response in Mexican-Americans. Mol Psychiatry 14:1105–1118 19844206 10.1038/mp.2009.92 PMC2834349

[CR27] Dong Y, Tao B, Xue X, Feng C, Ren Y, Ma H, Zhang J, Si Y, Zhang S, Liu S, Li H, Zhou J, Li G, Wang Z, Xie J, Zhu Z (2021) Molecular mechanism of *Epicedium* treatment for depression based on network pharmacology and molecular docking technology. BMC Complement Med Ther 21:222 34479552 10.1186/s12906-021-03389-w PMC8417989

[CR28] Dubey P, Pandey KB (2023) Neurotransmitter modulation by phytochemicals. In: Egbuna C, Rudrapal M (eds) Phytochemical drug discovery for central nervous system disorders, John Wiley & Sons, Inc, pp 311–325

[CR29] Edmondson DE, Binda C (2018) Monoamine oxidases. Subcell Biochem 87:117–139 29464559 10.1007/978-981-10-7757-9_5

[CR30] Ekici M, Güngör H, Mert D (2023) Kaempferol and isorhamnetin alleviate lipopolysaccharide-induced anxiety and depression-like behavioral in Balb/C mice. J Hellenic Vet Med Soc 74:5749–5760

[CR31] El-Ansari MA, Aboutabl EA, Farrag ARH, Sharaf M, Hawas UW, Soliman GM, El-Seed GS (2009) Phytochemical and pharmacological studies on *Leonotis leonurus*. Pharm Biol 47:894–902

[CR32] Elshamy S, Abdel Motaal A, Abdel-Halim M, Medhat D, Handoussa H (2021) Potential neuroprotective activity of *Mentha longifolia* L. in aluminum chloride‐induced rat model of Alzheimer’s disease. J Food Biochem 45:1770 33587299 10.1111/jfbc.13644

[CR33] Fan N-S, Fu J-J, Huang D-Q, Ma Y-L, Lu Z-Y, Jin R-C, Zheng P (2021) Resistance genes and extracellular proteins relieve antibiotic stress on the anammox process. Water Res 202:117453 34320444 10.1016/j.watres.2021.117453

[CR34] Fang G, Cheng C, Zhang M, Ma X, Yang S, Hou X, Deng J, Hou Y, Bai G (2021) The glucuronide metabolites of kaempferol and quercetin, targeting to the AKT PH domain, activate *AKT*/*GSK3β* signaling pathway and improve glucose metabolism. J Funct Foods 82:104501

[CR35] Fatriansyah JF, Boanerges AG, Kurnianto SR, Pradana AF, Fadilah, Surip SN (2022) Molecular dynamics simulation of ligands from *Anredera cordifolia* (Binahong) to the main protease (Mpro) of SARS-CoV-2. J Trop Med 2022:1178228 36457332 10.1155/2022/1178228 PMC9708379

[CR36] Ferenczy GG, Kellermayer M (2022) Contribution of hydrophobic interactions to protein mechanical stability. Comput Struct Biotechnol J 20:1946–1956 10.1016/j.csbj.2022.04.025 PMC9062142 35521554

[CR37] Ferré S, Belcher AM, Bonaventura J, Quiroz C, Sánchez-Soto M, Casadó-Anguera V, Cai N-S, Moreno E, Boateng CA, Keck TM, Florán B, Earley CJ, Ciruela F, Casadó V, Rubinstein M, Volkow ND (2022) Functional and pharmacological role of the dopamine D4 receptor and its polymorphic variants. Front Endocrinol 13:1014678 10.3389/fendo.2022.1014678 PMC9578002 36267569

[CR38] Gan XX, Zhong LK, Shen F, Feng JH, Li YY, Li SJ, Cai WS, Xu B (2021) Network pharmacology to explore the molecular mechanisms of *Prunella vulgaris* for treating Hashimoto’s thyroiditis. Front Pharmacol 12:700896 34690752 10.3389/fphar.2021.700896 PMC8527019

[CR39] Gao L-q, Xu J, Chen S-d (2020) *In silico* screening of potential Chinese herbal medicine against COVID-19 by targeting SARS-CoV-2 3CLpro and angiotensin converting enzyme II using molecular docking. Chin J Integr Med 26:527–532 32632717 10.1007/s11655-020-3476-x PMC7338104

[CR41] Gao Y, Mu J, Xu T, Linghu T, Zhao H, Tian J, Qin X (2021) Metabolomic analysis of the hippocampus in a rat model of chronic mild unpredictable stress-induced depression based on a pathway crosstalk and network module approach. J Pharm Biomed Anal 193:113755 33190083 10.1016/j.jpba.2020.113755

[CR40] Gao S, Lu J, Gu Y, Zhang Y, Wang C, Gao F, Dai Z, Xu S, Zhang J, Yang Y, Lei H (2024) Revealing the mechanism of *Hemerocallis citrina* baroni in depression treatment through integrated network pharmacology and transcriptomic analysis. Pharmaceuticals 17:1704 39770546 10.3390/ph17121704 PMC11677347

[CR42] Ghasemzadeh Rahbardar M, Hosseinzadeh H (2020) Therapeutic effects of rosemary (*Rosmarinus officinalis* L.) and its active constituents on nervous system disorders. Iran J Basic Med Sci 23:1100–1112 32963731 10.22038/ijbms.2020.45269.10541 PMC7491497

[CR43] Gholami M, Salmani RHG, Madadlou SK, Khoshraftar P, Buttar HS, Abbasi-Maleki S, Kaur P (2025) Antidepressant effects of medicinal plants and their pharmacologically active ingredients: a systematic review focused on the serotonergic system. In: Sobti RC, Wilson DW, Buttar HS, Télessy IG, Sobti A (eds) Molecular medicine and biomedical research in the era of precision medicine, Academic press, pp 261–278

[CR44] Gu X, Zhang G, Wang Q, Song J, Li Y, Xia C, Zhang T, Yang L, Sun J, Zhou M (2022) Integrated network pharmacology and hepatic metabolomics to reveal the mechanism of *Acanthopanax senticosus* against major depressive disorder. Front Cell Dev Biol 10:900637 10.3389/fcell.2022.900637 PMC9389016 35990602

[CR45] Güleç Ö, Duran HE, Arslan M, Yıldıztekin G, Ece A, Türkeş C (2025) Chalcone-inspired indole, carbazole, and phenothiazine hybrids as potent aldose reductase inhibitors with selective anticancer potential: rational design, synthesis, and multi-level characterization. Bioorg Chem 164:108861 40789257 10.1016/j.bioorg.2025.108861

[CR46] Gundogdu S, Duran HE, Arslan M, Çetinkaya BD, Türkeş C (2025) Fluorenyl-phthalimide hybrids as potent aldose reductase inhibitors with selective anticancer activity: rational design, synthesis, and molecular insights. Bioorg Chem 163:108689 40570667 10.1016/j.bioorg.2025.108689

[CR47] He Y, Han Y, Liao X, Zou M, Wang Y (2022) Biology of cyclooxygenase-2: an application in depression therapeutics. Front Psychol 13:1037588 10.3389/fpsyt.2022.1037588 PMC9684729 36440427

[CR48] Huang T, Liu Y, Zhang C (2019) Pharmacokinetics and bioavailability enhancement of Baicalin: a review. Eur J Drug Metab Pharmacokinet 44:159–168 30209794 10.1007/s13318-018-0509-3

[CR49] Jatana N, Thukral L, Latha N (2015) Structural signatures of DRD4 mutants revealed using molecular dynamics simulations: implications for drug targeting. J Mol Model 22:14 26680992 10.1007/s00894-015-2868-x

[CR50] Joy N (2019) Molecular mimicry of anti-migraine drugs with the neurotransmitters, dopamine (DA) & serotonin (5-HT) and its role in the treatment of migraine. University of Canterbury

[CR51] Kambeitz JP, Howes OD (2015) The serotonin transporter in depression: meta-analysis of in vivo and post mortem findings and implications for understanding and treating depression. J Affect Disord 186:358–366 26281039 10.1016/j.jad.2015.07.034

[CR52] Kaur B, Singh P (2022) Inflammation: biochemistry, cellular targets, anti-inflammatory agents and challenges with special emphasis on cyclooxygenase-2. Bioorg Chem 121:105663 35180488 10.1016/j.bioorg.2022.105663

[CR53] Kautto M, Kampman O, Mononen N, Lehtimäki T, Haraldsson S, Koivisto PA, Leinonen E (2015) Serotonin transporter (5-HTTLPR) and norepinephrine transporter (NET) gene polymorphisms: susceptibility and treatment response of electroconvulsive therapy in treatment resistant depression. Neurosci Lett 590:116–120 25650523 10.1016/j.neulet.2015.01.077

[CR54] Keiser MJ, Roth BL, Armbruster BN, Ernsberger P, Irwin JJ, Shoichet BK (2007) Relating protein pharmacology by ligand chemistry. Nat Biotechnol 25:197–206 17287757 10.1038/nbt1284

[CR55] Khade OS, Sruthi K, Sonkar RM, Gade PS, Bhatt P (2023) Plant secondary metabolites: extraction, screening, analysis and their bioactivity. Int J Herb Med 11:01–17

[CR57] Kim S, Chen J, Cheng T, Gindulyte A, He J, He S, Li Q, Shoemaker BA, Thiessen PA, Yu B, Zaslavsky L, Zhang J, Bolton EE (2019) PubChem 2019 update: improved access to chemical data. Nucleic Acids Res 47:D1102–D1109 30371825 10.1093/nar/gky1033 PMC6324075

[CR56] Kim MH, Kwon SY, Woo S-Y, Seo WD, Kim DY (2021) Antioxidative effects of Chrysoeriol via activation of the Nrf2 signaling pathway and modulation of mitochondrial function. Molecules 26:313. 10.3390/molecules26020313 33435366 10.3390/molecules26020313 PMC7826659

[CR58] Kongolo Kalemba MR, Makhuvele R, Njobeh PB (2024) Phytochemical screening, antioxidant activity of selected methanolic plant extracts and their detoxification capabilities against AFB_1_ toxicity. Heliyon 10:e24435 10.1016/j.heliyon.2024.e24435 PMC10835242 38312698

[CR59] Korhonen T, Loukola A, Wedenoja J, Nyman E, Latvala A, Broms U, Häppölä A, Paunio T, Schrage AJ, Vink JM (2014) Role of nicotine dependence in the association between the dopamine receptor gene *DRD3* and major depressive disorder. PLoS ONE 9:e98199 24927283 10.1371/journal.pone.0098199 PMC4057087

[CR60] Koshland DE Jr (1995) The key–lock theory and the induced fit theory. Angewandte Chemie Int Ed Engl 33:2375–2378

[CR61] Kulikov AV, Gainetdinov RR, Ponimaskin E, Kalueff AV, Naumenko VS, Popova NK (2018) Interplay between the key proteins of serotonin system in SSRI antidepressants efficacy. Expert Opin Ther Targets 22:319–330 29542343 10.1080/14728222.2018.1452912

[CR62] Lai Z, Tsugawa H, Wohlgemuth G, Mehta S, Mueller M, Zheng Y, Ogiwara A, Meissen J, Showalter M, Takeuchi K, Kind T, Beal P, Arita M, Fiehn O (2018) Identifying metabolites by integrating metabolome databases with mass spectrometry cheminformatics. Nat Methods 15:53–56 29176591 10.1038/nmeth.4512 PMC6358022

[CR63] Lee S-Y, Chen S-L, Chen S-H, Chu C-H, Chang Y-H, Lin S-H, Huang S-Y, Tzeng N-S, Kuo P-H, Lee IH, Yeh TL, Yang YK, Lu R-B (2012) Interaction of the *DRD3* and *BDNF* gene variants in subtyped bipolar disorder. Prog Neuropsychopharmacol Biol Psychiatry 39:382–387 22877924 10.1016/j.pnpbp.2012.07.015

[CR64] León SL, Croes EA, Sayed-Tabatabaei FA, Claes S, Van Broeckhoven C, van Duijn CM (2005) The dopamine D4 receptor gene 48-base-pair-repeat polymorphism and mood disorders: a meta-analysis. Biol Psychiatry 57:999–1003 15860340 10.1016/j.biopsych.2005.01.030

[CR66] Li X, Wei S, Niu S, Ma X, Li H, Jing M, Zhao Y (2022) Network pharmacology prediction and molecular docking-based strategy to explore the potential mechanism of *Huanglian Jiedu* Decoction against sepsis. Comput Biol Med 144:105389 35303581 10.1016/j.compbiomed.2022.105389

[CR65] Li J, Wang N, Huang Q, Jiao C, Liu W, Yang C, Tang X, Mao R, Zhou Q, Ding Y, Shan B, Xu L (2025) Acute treatment with salvianolic acid a produces neuroprotection in stroke models by inducing excitatory long-term synaptic depression. ACS Chem Neurosci 16:659–672 39888337 10.1021/acschemneuro.4c00720

[CR68] Liu P-L, Song A-R, Dong C-D, Chu Q, Xu B-L, Liu J-M, Yan Z-J (2021a) Network pharmacology study on the mechanism of the herb pair of prepared *Rehmannia* root-Chinese arborvitae kernel for anxiety disorders. Annals Palliat Med 10:3313327–3313327 10.21037/apm-21-531 33849116

[CR69] Liu X-j, Wang Y-z, Wei F-x, Lv M, Qu P, Chen S-j, Li S-y, Qin X (2021b) The synergistic anti-depression effects of different efficacy groups of Xiaoyaosan as demonstrated by the integration of network pharmacology and serum metabolomics. J Pharm Biomed Anal 197:113949 33618131 10.1016/j.jpba.2021.113949

[CR67] Liu M, Fu L, Fu H, Chen Y, Wu M, Liu H (2024) Pharmacological underlying mechanisms of the anticancer effect of licorice: bioinformatics and experimental verification. J Holist Integr Pharm 5:45–55

[CR70] Lockwood L, Su S, Youssef NA (2022) Chap. 15 - epigenetics, stress, and depression. In: Youssef NA (ed) Epigenetics of Stress and Stress Disorders, Academic Press, pp 225–238

[CR71] Ma Z, Zeng P, Feng H, Ni L (2024) Network pharmacology and molecular docking to explore the treatment potential and molecular mechanism of Si-Miao decoction against gouty arthritis. Medicine 103:e38221 39259129 10.1097/MD.0000000000038221 PMC11142817

[CR72] Mallick K, Banerjee S (2023) Plants affecting serotonergic neurotransmission. In: Dhara AK, Mandal SC (eds) Role of herbal medicines: management of lifestyle diseases. Springer Nature Singapore, Singapore, pp 211–229

[CR73] Marafie SK, Alshawaf E, Al-Mulla F, Abubaker J, Mohammad A (2024) Targeting mTOR Kinase with natural compounds: potent ATP-competitive inhibition through enhanced binding mechanisms. Pharmaceuticals 17:1677. 10.3390/ph17121677 39770519 10.3390/ph17121677 PMC11677242

[CR74] Mathew B, Oh JM, Parambi DGT, Sudevan ST, Kumar S, Kim H (2024) Enzyme inhibition assays for monoamine oxidase. In: Ray SK (ed) Neuroprotection: method and protocols. Springer US, New York, NY, pp 329–336 10.1007/978-1-0716-3662-6_24 38427248

[CR75] Nakajima M, Hattori E, Yamada K, Iwayama Y, Toyota T, Iwata Y, Tsuchiya KJ, Sugihara G, Hashimoto K, Watanabe H (2007) Association and synergistic interaction between promoter variants of the *DRD4* gene in Japanese schizophrenics. J Hum Genet 52:86–91 17089069 10.1007/s10038-006-0084-3 PMC1705471

[CR76] Naoi M, Wu Y, Maruyama W, Shamoto-Nagai M (2025) Phytochemicals modulate biosynthesis and function of serotonin, dopamine, and norepinephrine for treatment of monoamine neurotransmission-related psychiatric diseases. Int J Mol Sci 26:2916. 10.3390/ijms26072916 40243512 10.3390/ijms26072916 PMC11988947

[CR77] Naveed M, Hejazi V, Abbas M, Kamboh AA, Khan GJ, Shumzaid M, Ahmad F, Babazadeh D, FangFang X, Modarresi-Ghazani F, WenHua L, XiaoHui Z (2018) Chlorogenic acid (CGA): a pharmacological review and call for further research. Biomed Pharmacother 97:67–74 29080460 10.1016/j.biopha.2017.10.064

[CR78] Nazem V, Sabzalian MR, Saeidi G, Rahimmalek M (2019) Essential oil yield and composition and secondary metabolites in self- and open-pollinated populations of mint (*Mentha* spp). Ind Crops Prod 130:332–340

[CR79] Nsuala BN, Enslin G, Viljoen A (2015) Wild cannabis: a review of the traditional use and phytochemistry of *Leonotis leonurus*. J Ethnopharmacol 174:520–539 26292023 10.1016/j.jep.2015.08.013

[CR80] Otasek D, Morris JH, Bouças J, Pico AR, Demchak B (2019) Cytoscape automation: empowering workflow-based network analysis. Genome Biol 20:185 31477170 10.1186/s13059-019-1758-4 PMC6717989

[CR81] Paudel P, Seong SH, Zhou Y, Park CH, Yokozawa T, Jung HA, Choi JS (2018) Rosmarinic acid derivatives’ inhibition of glycogen synthase kinase-3β Is the pharmacological basis of *Kangen-karyu* in Alzheimer’s disease. Molecules 23:2919. 10.3390/molecules23112919 30413117 10.3390/molecules23112919 PMC6278281

[CR82] Phootha N, Yongparnichkul N, Fang Z, Gan R-Y, Zhang P (2022) Plants and phytochemicals potentials in tackling anxiety: a systematic review. Phytomed Plus 2:100375

[CR83] Piñero J, Ramírez-Anguita JM, Saüch-Pitarch J, Ronzano F, Centeno E, Sanz F, Furlong LI (2020) The DisGeNET knowledge platform for disease genomics: 2019 update. Nucleic Acids Res 48:D845–D855 31680165 10.1093/nar/gkz1021 PMC7145631

[CR84] Pritchard AL, Ratcliffe L, Sorour E, Haque S, Holder R, Bentham P, Lendon CL (2009) Investigation of dopamine receptors in susceptibility to behavioural and psychological symptoms in Alzheimer’s disease. Int J Geriatr Psychiatry 24:1020–1025 19235789 10.1002/gps.2214

[CR85] Rebas E, Rzajew J, Radzik T, Zylinska L (2020) Neuroprotective polyphenols: a modulatory action on neurotransmitter pathways. Curr Neuropharmacol 18:431–445 31903883 10.2174/1570159X18666200106155127 PMC7457434

[CR86] Ripke S, Sanders AR, Kendler KS, Levinson DF, Sklar P, Holmans PA, Lin DY, Duan J, Ophoff RA, Andreassen OA, Scolnick E, Cichon S, St. Clair D, Corvin A, Gurling H, Werge T, Rujescu D, Blackwood DHR, Pato CN, Malhotra AK, Purcell S, Dudbridge F, Neale BM, Rossin L, Visscher PM, Posthuma D, Ruderfer DM, Fanous A, Stefansson H, Steinberg S, Mowry BJ, Golimbet V, De Hert M, Jönsson EG, Bitter I, Pietiläinen OPH, Collier DA, Tosato S, Agartz I, Albus M, Alexander M, Amdur RL, Amin F, Bass N, Bergen SE, Black DW, Børglum AD, Brown MA, Bruggeman R, Buccola NG, Byerley WF, Cahn W, Cantor RM, Carr VJ, Catts SV, Choudhury K, Cloninger CR, Cormican P, Craddock N, Danoy PA, Datta S, De Haan L, Demontis D, Dikeos D, Djurovic S, Donnelly P, Donohoe G, Duong L, Dwyer S, Fink-Jensen A, Freedman R, Freimer NB, Friedl M, Georgieva L, Giegling I, Gill M, Glenthøj B, Godard S, Hamshere M, Hansen M, Hansen T, Hartmann AM, Henskens FA, Hougaard DM, Hultman CM, Ingason A, Jablensky AV, Jakobsen KD, Jay M, Jürgens G, Kahn RS, Keller MC, Kenis G, Kenny E, Kim Y, Kirov GK, Konnerth H, Konte B, Krabbendam L, Krasucki R, Lasseter VK, Laurent C, Lawrence J, Lencz T, Lerer FB, Liang KY, Lichtenstein P, Lieberman JA, Linszen DH, Lönnqvist J, Loughland CM, MacLean AW, Maher BS, Maier W, Mallet J, Malloy P, Mattheisen M, Mattingsdal M, McGhee KA, McGrath JJ, McIntosh A, McLean DE, McQuillin A, Melle I, Michie PT, Milanova V, Morris DW, Mors O, Mortensen PB, Moskvina V, Muglia P, Myin-Germeys I, Nertney DA, Nestadt G, Nielsen J, Nikolov I, Nordentoft M, Norton N, Nöthen MM, O’Dushlaine CT, Olincy A, Olsen L, O’Neill FA, Ørntoft TF, Owen MJ, Pantelis C, Papadimitriou G, Pato MT, Peltonen L, Petursson H, Pickard B, Pimm J, Pulver AE, Puri V, Quested D, Quinn EM, Rasmussen HB, Réthelyi JM, Ribble R, Rietschel M, Riley BP, Ruggeri M, Schall U, Schulze TG, Schwab SG, Scott RJ, Shi J, Sigurdsson E, Silverman JM, Spencer CCA, Stefansson K, Strange A, Strengman E, Stroup TS, Suvisaari J, Terenius L, Thirumalai S, Thygesen JH, Timm S, Toncheva D, Van Den Oord E, Van Os J, Van Winkel R, Veldink J, Walsh D, Wang AG, Wiersma D, Wildenauer DB, Williams HJ, Williams NM, Wormley B, Zammit S Sullivan PF, O’Donovan MC, Daly MJ, Gejman PV (2011) Genome-wide association study identifies five new schizophrenia loci. Nat Genet 43:969–978 21926974 10.1038/ng.940 PMC3303194

[CR87] Roshanak S, Rahimmalek M, Goli SA (2016) Evaluation of seven different drying treatments in respect to total flavonoid, phenolic, vitamin C content, chlorophyll, antioxidant activity and color of green tea (*Camellia sinensis* or *C*. *assamica*) leaves. J Food Sci Technol 53:721–729 26787992 10.1007/s13197-015-2030-x PMC4711456

[CR88] Shen J, Qu C, Xu L, Sun H, Zhang J (2019) Resveratrol exerts a protective effect in chronic unpredictable mild stress–induced depressive-like behavior: involvement of the AKT/GSK3β signaling pathway in hippocampus. Psychopharmacology 236:591–602 30374891 10.1007/s00213-018-5087-1

[CR90] Shi Y, Chen D, Ma S, Xu H, Deng L (2021) Identification of potential biomarkers of depression and network pharmacology approach to investigate the mechanism of key genes and therapeutic traditional Chinese medicine in the treatment of depression. Evid-Based Complement Altern Med 2021:2165632 10.1155/2021/2165632 PMC8741373 35003290

[CR89] Shi Q, Malik H, Crawford RM, Streeter J, Wang J, Huo R, Shih JC, Chen B, Hall D, Abel ED, Song L-S, Anderson EJ (2024) Cardiac monoamine oxidase-A inhibition protects against catecholamine-induced ventricular arrhythmias via enhanced diastolic calcium control. Cardiovascular Res 120:596–611 10.1093/cvr/cvae012 PMC11074799 38198753

[CR91] Short B, Fong J, Galvez V, Shelker W, Loo CK (2018) Side-effects associated with ketamine use in depression: a systematic review. Lancet Psychiatry 5:65–78 28757132 10.1016/S2215-0366(17)30272-9

[CR92] Silva dos Santos J, Gonçalves Cirino JP, de Oliveira Carvalho P, Ortega MM (2021) The pharmacological action of kaempferol in central nervous system diseases: a review. Front Pharmacol 11:565700 33519431 10.3389/fphar.2020.565700 PMC7838523

[CR93] Sivaraman B, Maji L, Chagaleti BK, Muthukumaradoss K (2026) Exploration of a Novel Imidazo[4,5-b]pyridine–dihydropyrimidinone hybrids as aurora kinase a inhibitors: integrated pharmacophore modelling, docking and simulation approaches. Mol Divers. 10.1007/s11030-026-11537-y 10.1007/s11030-026-11537-y 41961391

[CR94] Stafford GI, Pedersen ME, van Staden J, Jäger AK (2008) Review on plants with CNS-effects used in traditional South African medicine against mental diseases. J Ethnopharmacol 119:513–537 18775771 10.1016/j.jep.2008.08.010

[CR95] Szklarczyk D, Gable AL, Nastou KC, Lyon D, Kirsch R, Pyysalo S, Doncheva NT, Legeay M, Fang T, Bork P, Jensen LJ, von Mering C (2021) The STRING database in 2021: customizable protein-protein networks, and functional characterization of user-uploaded gene/measurement sets. Nucleic Acids Res 49:D605–D612 33237311 10.1093/nar/gkaa1074 PMC7779004

[CR96] Tao W, Xu X, Wang X, Li B, Wang Y, Li Y, Yang L (2013) Network pharmacology-based prediction of the active ingredients and potential targets of Chinese herbal *Radix Curcumae* formula for application to cardiovascular disease. J Ethnopharmacol 145:1–10 23142198 10.1016/j.jep.2012.09.051

[CR97] Tsugawa H, Cajka T, Kind T, Ma Y, Higgins B, Ikeda K, Kanazawa M, VanderGheynst J, Fiehn O, Arita M (2015) MS-DIAL: data-independent MS/MS deconvolution for comprehensive metabolome analysis. Nat Methods 12:523–526 25938372 10.1038/nmeth.3393 PMC4449330

[CR98] Tzeng S-R, Kalodimos CG (2011) Protein dynamics and allostery: an NMR view. Curr Opin Struct Biol 21:62–67 21109422 10.1016/j.sbi.2010.10.007

[CR99] Vikhar Danish Ahmad A, Khan SW, Ali SA, Yasar Q (2024) Network pharmacology combined with molecular docking and experimental verification to elucidate the effect of flavan-3-ols and aromatic resin on anxiety. Sci Rep 14:9799 38684743 10.1038/s41598-024-58877-z PMC11058257

[CR100] Wang C, Lin H, Yang N, Wang H, Zhao Y, Li P, Liu J, Wang F (2019) Effects of Platycodins folium on depression in mice based on a UPLC-Q/TOF-MS serum assay and hippocampus metabolomics. Molecules 24:1712. 10.3390/molecules24091712 31052597 10.3390/molecules24091712 PMC6540008

[CR103] Wang W, Song J, Chuai Y, Chen F, Song C, Shu M, Wang Y, Li Y, Zhai X, Han S (2021) The mining and construction of a knowledge base for gene-disease association in mitochondrial diseases. Sci Rep 11:23909 34903783 10.1038/s41598-021-03249-0 PMC8668972

[CR102] Wang H, Liu J, He J, Huang D, Xi Y, Xiao T, Ouyang Q, Zhang S, Wan S, Chen X (2022a) Potential mechanisms underlying the therapeutic roles of *sinisan* formula in depression: based on network pharmacology and molecular docking study. Front Psychiatry 13:1063489 36440424 10.3389/fpsyt.2022.1063489 PMC9681910

[CR104] Wang W, Zhang Y, Yang Y, Gu L (2022b) Network pharmacology and molecular docking to explore the mechanism of Kangxian decoction for epilepsy. Evid-Based Complement Alter Med 2022:3333878 10.1155/2022/3333878 PMC9525756 36193133

[CR101] Wang D, Ren Y-M, Guo Y-X, Zhang Z-Q, Sui H-, Zhang H-Y (2024) The effects of baicalin in depression: preclinical evidence construction based on meta-analysis. Front Pharmacol 15:1425094 39114351 10.3389/fphar.2024.1425094 PMC11303225

[CR105] Xiang L, Szebeni K, Szebeni A, Klimek V, Stockmeier CA, Karolewicz B, Kalbfleisch J, Ordway GA (2008) Dopamine receptor gene expression in human amygdaloid nuclei: elevated D4 receptor mRNA in major depression. Brain Res 1207:214–224 18371940 10.1016/j.brainres.2008.02.009 PMC2577810

[CR106] Yan T, He B, Wan S, Xu M, Yang H, Xiao F, Bi K, Jia Y (2017) Antidepressant-like effects and cognitive enhancement of *Schisandra chinensis* in chronic unpredictable mild stress mice and its related mechanism. Sci Rep 7:6903 28761074 10.1038/s41598-017-07407-1 PMC5537344

[CR108] Yang L, Zhao Y, Qu R, Fu Y, Zhou C, Yu J (2023) A network pharmacology and molecular docking approach to reveal the mechanism of Chaihu Anxin capsule in depression. Front Endocrinol 14:1256045 10.3389/fendo.2023.1256045 PMC10513492 37745719

[CR107] Yang F, Yang Z, Yan Y, Gu Y, Wang P, Wang M, Chen J, Du X, Wang G (2024) Exploring the mechanism of fibrates regulating HIF-1A in the treatment of ischemic stroke based on network pharmacology and molecular docking. BMC Res Notes 17:387 39726005 10.1186/s13104-024-07031-z PMC11670374

[CR109] Ylilauri M, Pentikäinen OT (2013) MMGBSA as a tool To understand the binding affinities of filamin–peptide interactions. J Chem Inf Model 53:2626–2633 23988151 10.1021/ci4002475

[CR110] Young SN, Leyton M (2002) The role of serotonin in human mood and social interaction: Insight from altered tryptophan levels. Pharmacol Biochem Behav 71:857–865 11888576 10.1016/s0091-3057(01)00670-0

[CR111] Zareei S, Pourmand S, Eskandarzadeh M, Massahi S (2024) *In silico* anti-alzheimer study of phytochemicals from Lamiaceae family through *GSK3*-β inhibition. Sci Rep 14:834 38191548 10.1038/s41598-023-47069-w PMC10774376

[CR113] Zhang K, Yang C, Xu Y, Sun N, Yang H, Liu J, Xu Q, Shen Y (2010) Genetic association of the interaction between the *BDNF* and *GSK3B* genes and major depressive disorder in a Chinese population. J Neural Transm 117:393–401 20033742 10.1007/s00702-009-0360-4

[CR114] Zhang L, Wang Y, Li D, Ho C-T, Li J, Wan X (2016) The absorption, distribution, metabolism and excretion of procyanidins. Food Funct 7:1273–1281 26814915 10.1039/c5fo01244a

[CR112] Zhang C, Niu Z, He Z, Ding Y, Wu G, Wu H, Chen W, Dong C, Ye Z, Gu F, Hu W (2024) Molecular interaction of soybean protein and piperine by computational docking analyses. Food Hydrocolloids 146:109249

[CR115] Zhang S, Liu K, Liu Y, Hu X, Gu X (2025) The role and application of bioinformatics techniques and tools in drug discovery. Front Pharmacol 16:1547131 40017606 10.3389/fphar.2025.1547131 PMC11865229

[CR116] Zhao Q, Dai W, Chen H, Jacobs R, Zlokovic B, Lund B, Montagne A, Bonnin A (2022) Prenatal disruption of blood–brain barrier formation via cyclooxygenase activation leads to lifelong brain inflammation. Proc Nat Acad Sci 119:e2113310119 35377817 10.1073/pnas.2113310119 PMC9169666

[CR117] Zheng Y, Wang CY, Wang SY, Zheng W (2003) Effect of high-oxygen atmospheres on blueberry phenolics, anthocyanins, and antioxidant capacity. J Agric Food Chem 51:7162–7169 14611188 10.1021/jf030440k

[CR118] Zhong J, Li G, Xu H, Wang Y, Shi M (2019) Baicalin ameliorates chronic mild stress-induced depression-like behaviors in mice and attenuates inflammatory cytokines and oxidative stress. Braz J Med Biol Res 52:e8434 31241715 10.1590/1414-431X20198434 PMC6596363

[CR119] Zognjani B, Nixha AR, Duran HE, Arslan M, Yıldıztekin G, Ece A, Türkeş C (2025) N-substituted phthalimide–carboxylic acid hybrids as dual-targeted aldose reductase inhibitors: Synthesis, mechanistic insights, and cancer-relevant profiling. Bioorg Chem 163:108788 40716159 10.1016/j.bioorg.2025.108788

