# Supplementary material for: Therapeutic potential of Leonotis leonurus and Mentha longifolia in depression: insights from network pharmacology and molecular dynamics stimulation-based study
Source: In Silico Pharmacol. 2026 Jul 13;14(2):193. doi: 10.1007/s40203-026-00689-2 (PMC13365280; doi:10.1007/s40203-026-00689-2)
Supplement: Supplementary file 1 — Supplementary Material 1 [file 40203_2026_689_MOESM1_ESM.docx]

**Supplementary files**


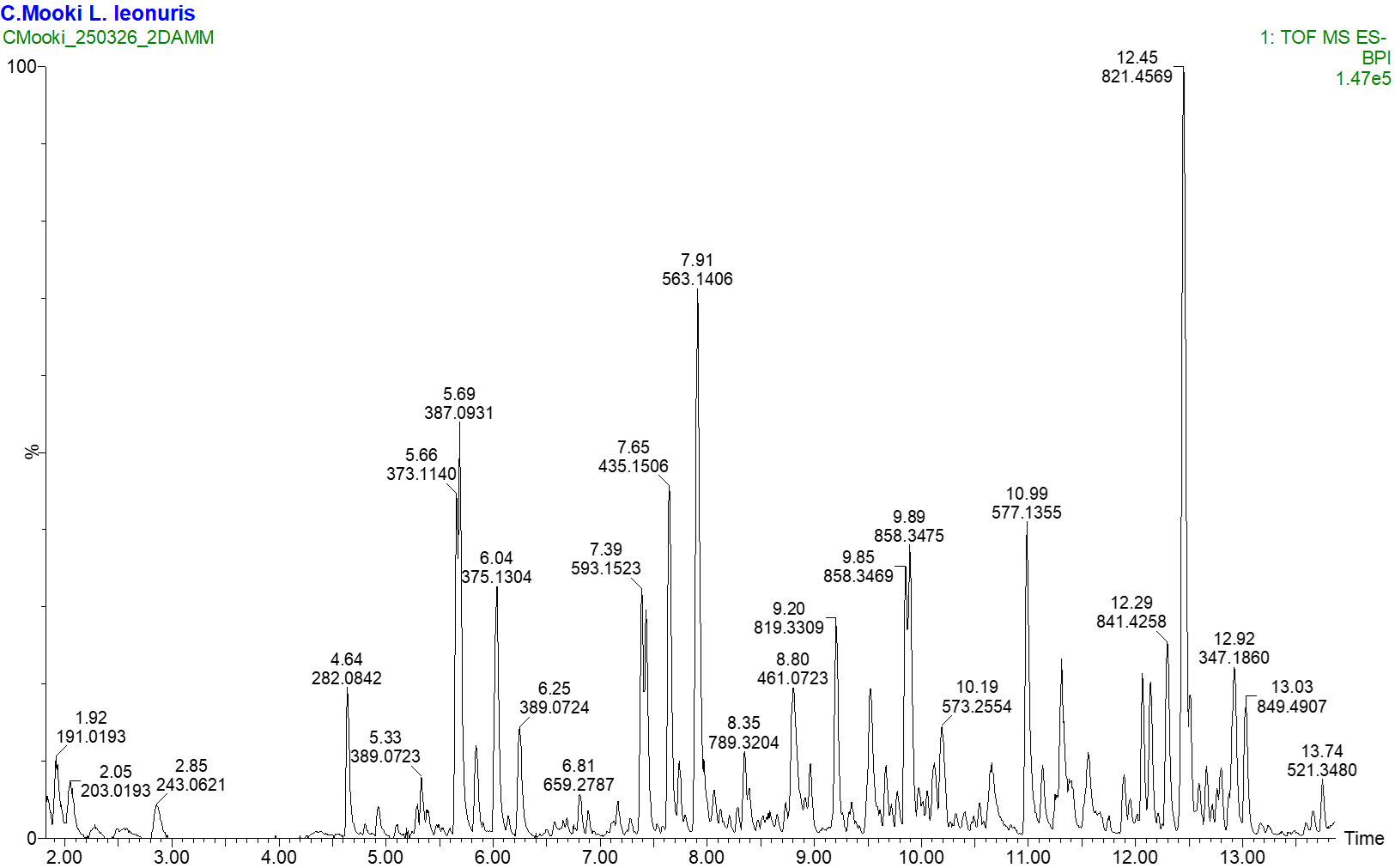


**Figure S1**: Representative chromatograms obtained for *Leonotis leonurus* powder using liquid chromatography-mass spectrometry (LC-MS).


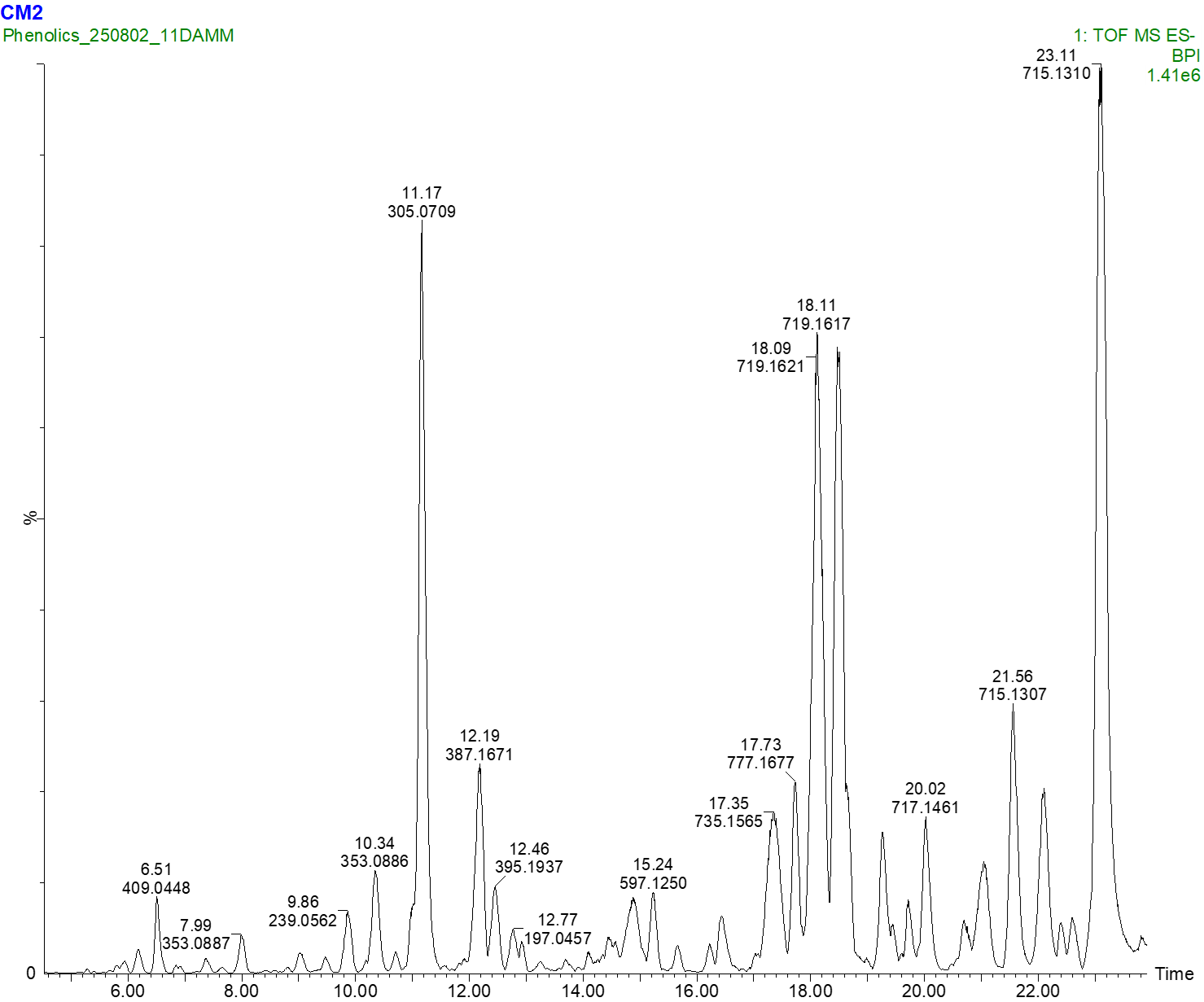


**Figure S2**: Representative chromatograms obtained for *Mentha longifolia* powder using liquid chromatography-mass spectrometry (LC-MS).


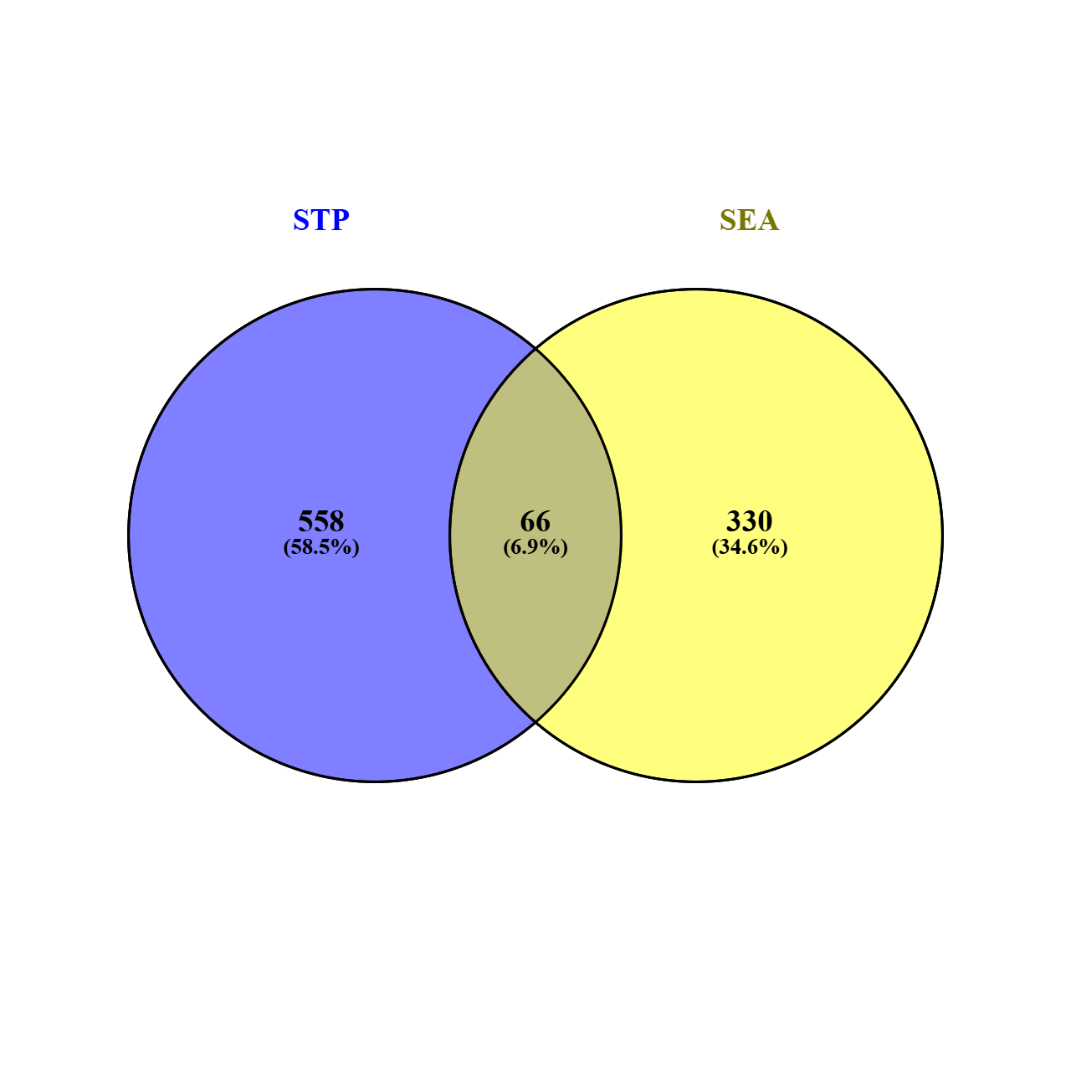

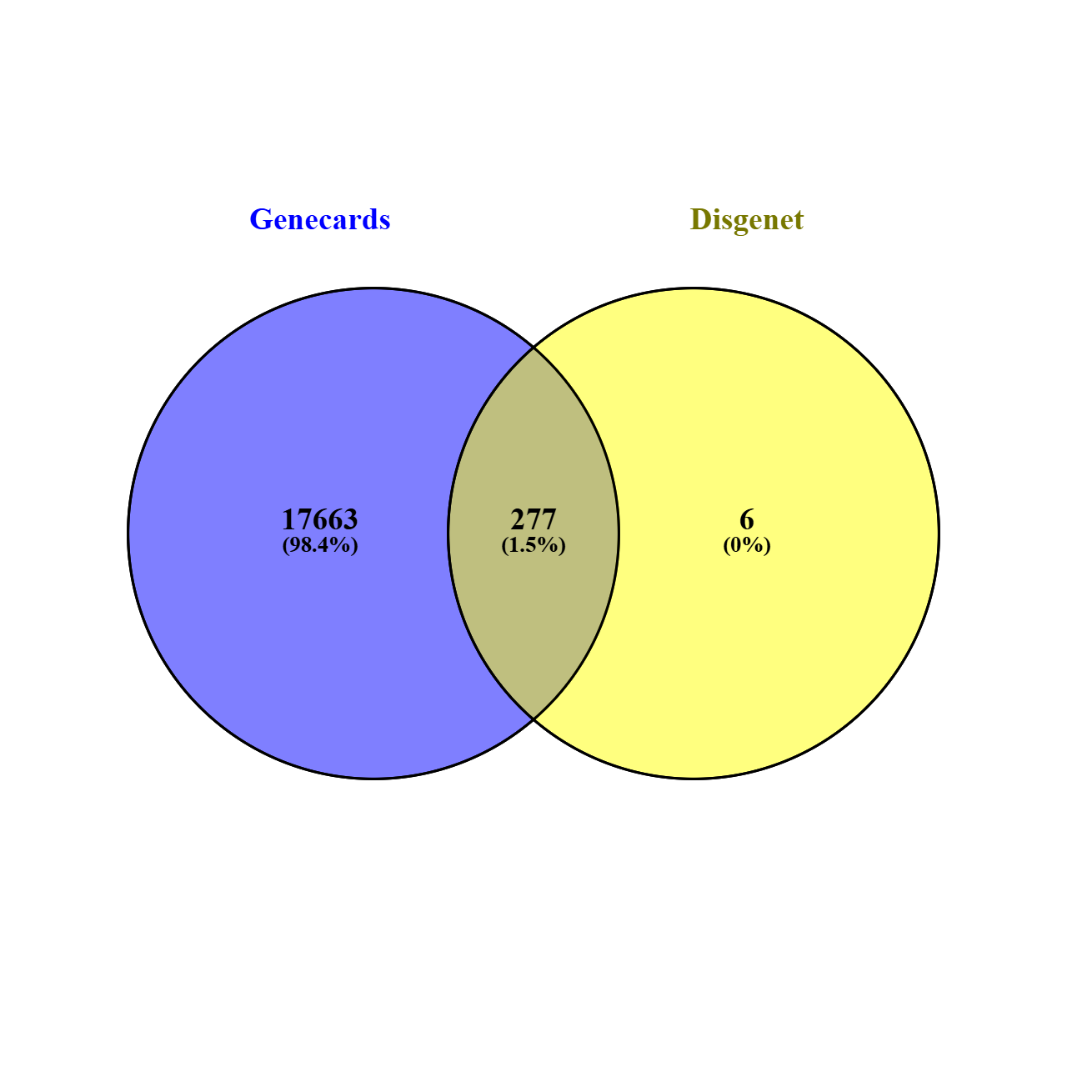


**B**

**A**


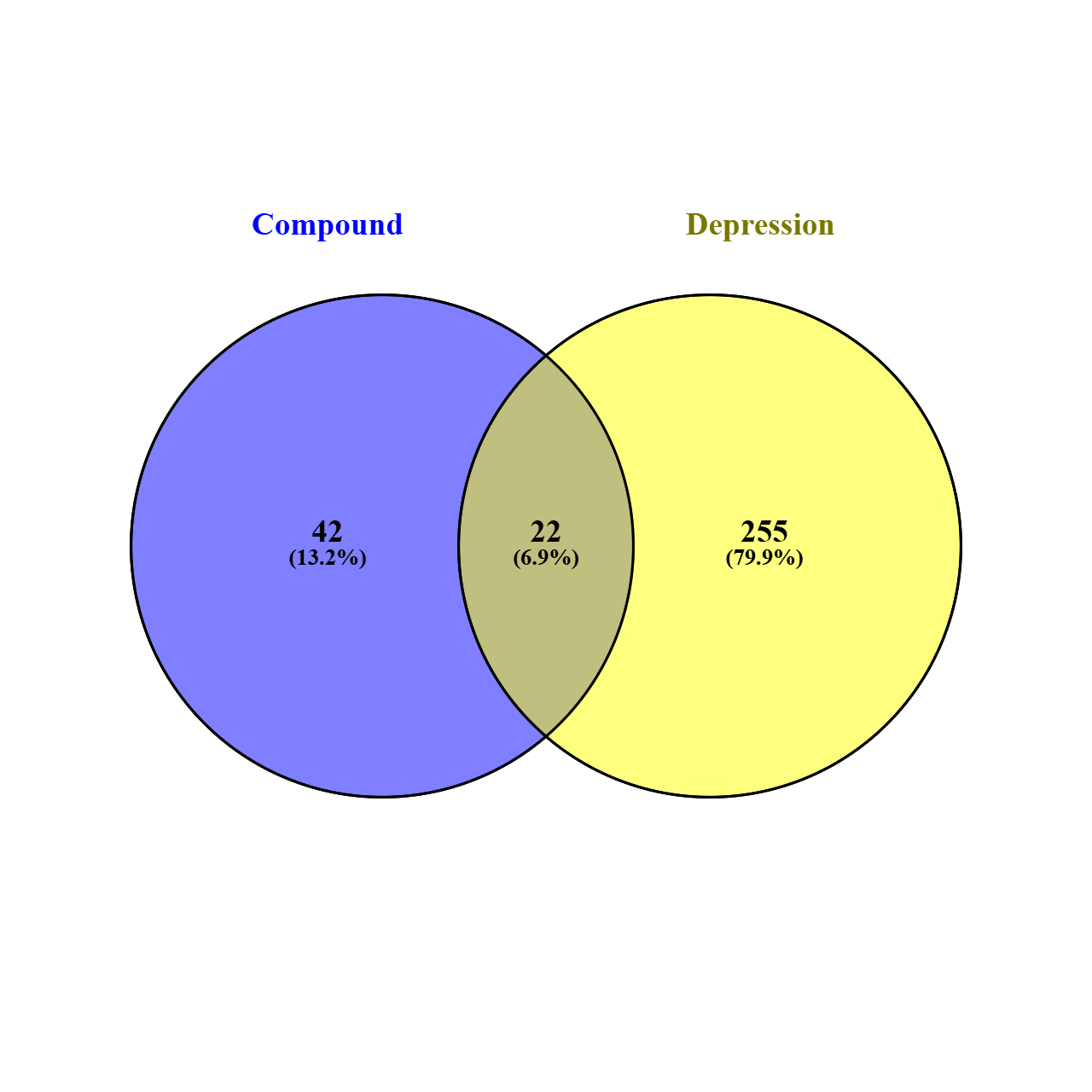


**C**

**Figure S3**: Identification of common target genes of *Leonotis leonurus* in relation to depression. (**A**) Venn diagram showing the overlapping genes associated with *L. leonurus* compounds predicted from STP and SEA databases. (**B**) Depression-related genes obtained from the GeneCards and DisGeNET databases. (**C**) Intersection analysis between *L. leonurus* compound targets and depression-related genes, highlighting the common targets potentially involved in the treatment of depression.


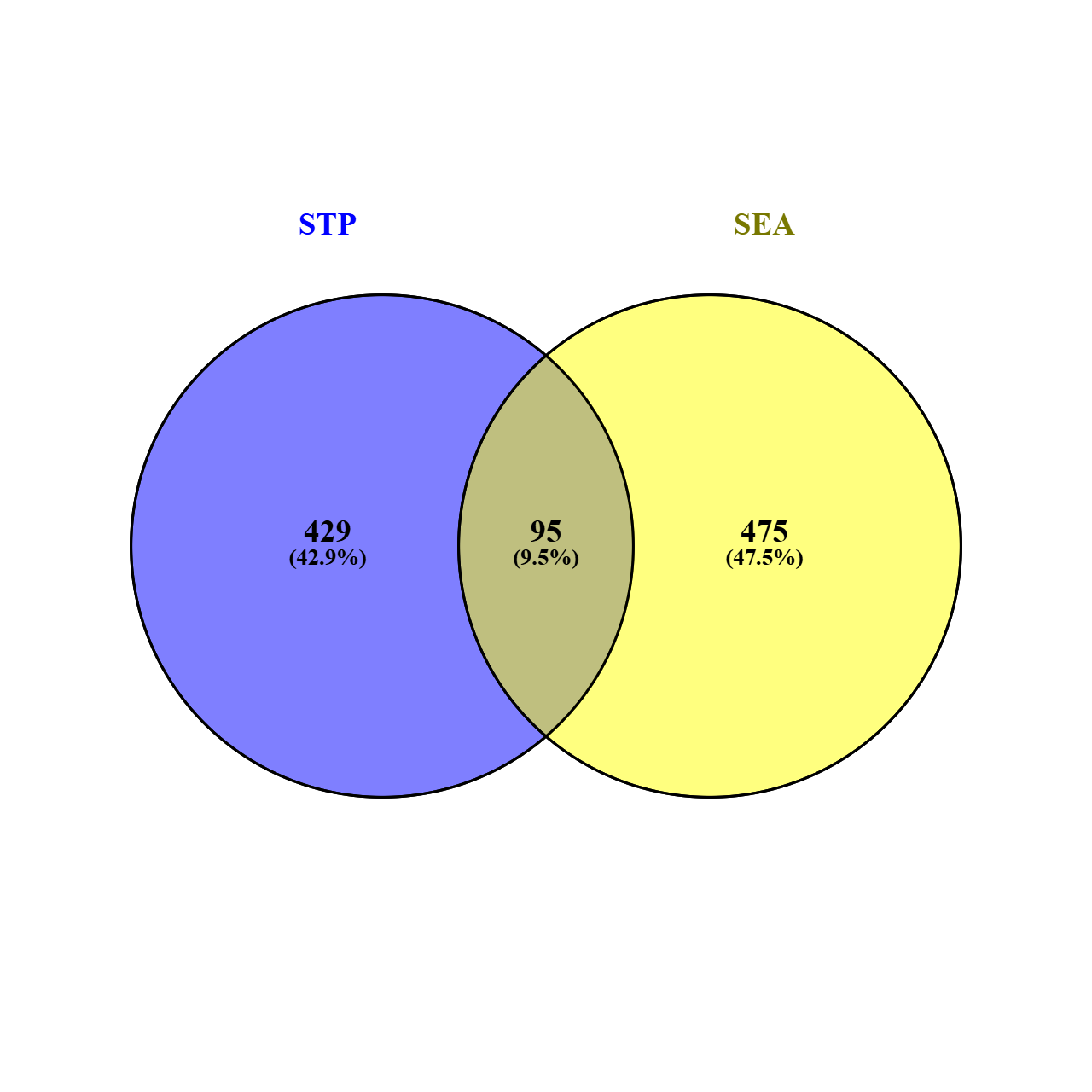


**A**


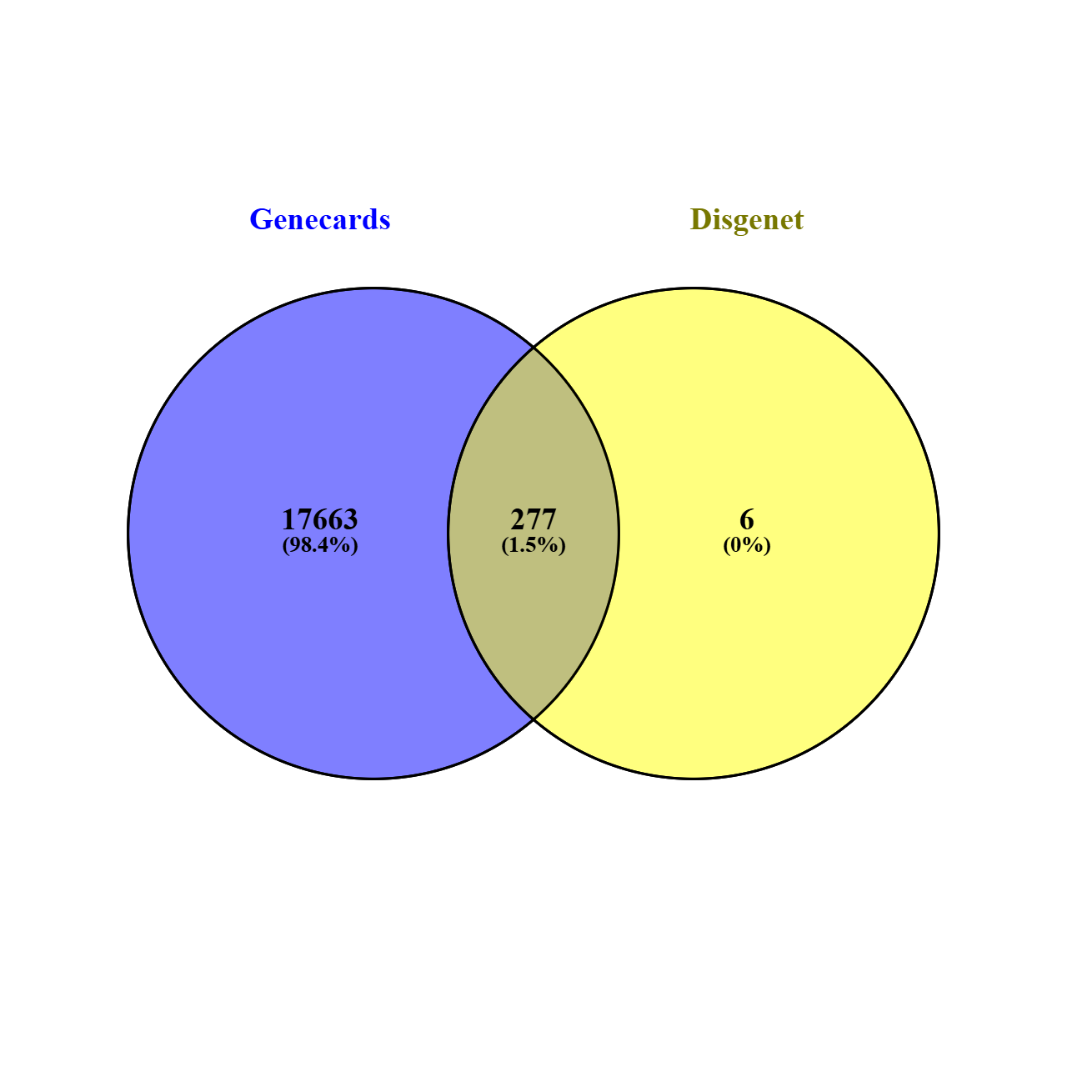


**B**


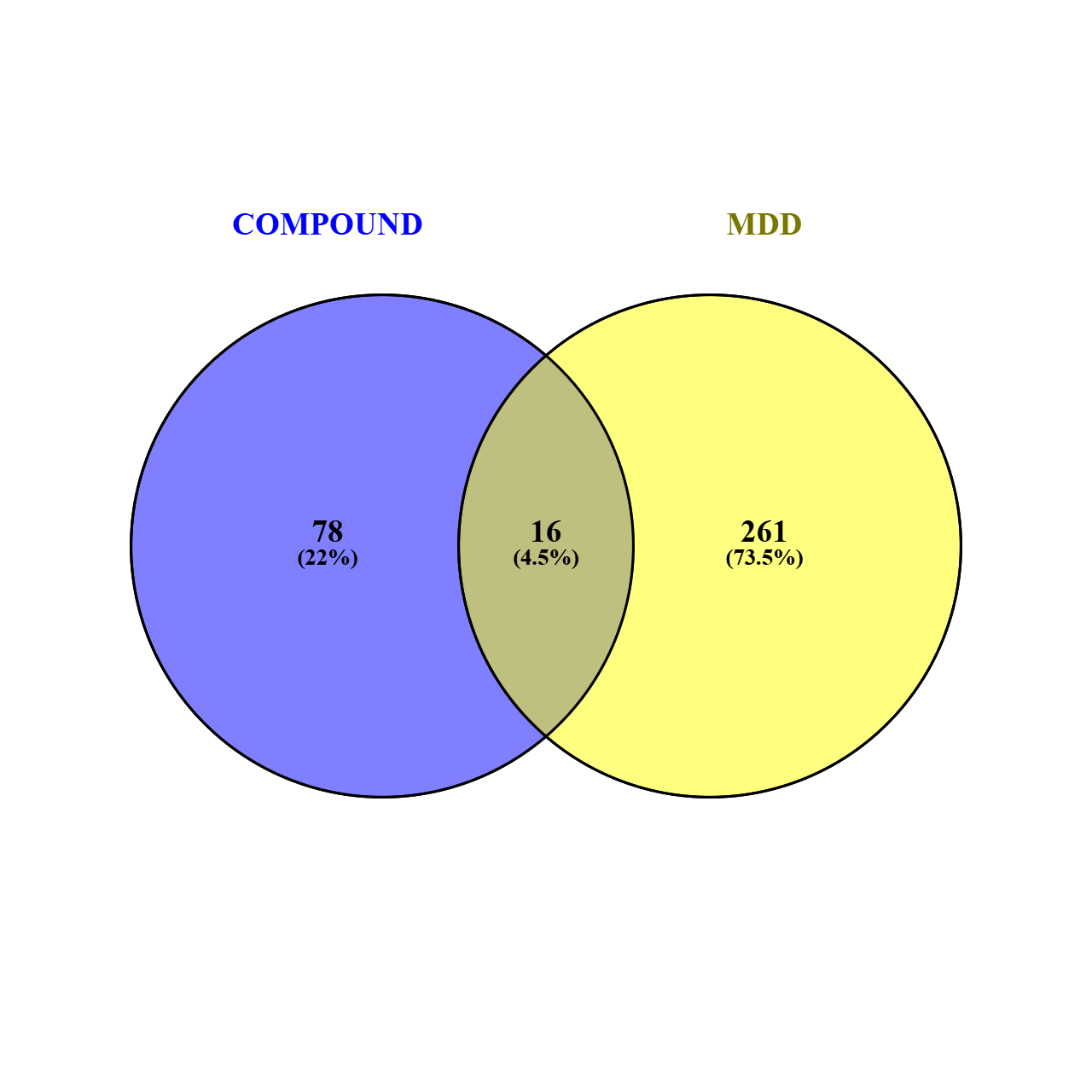


**C**

**Figure S4**. Identification of common target genes of *Mentha longifolia* associated with depression. (**A**) Venn diagram illustrating overlapping targets predicted from STP and SEA databases. (**B**) Depression-related genes retrieved from GeneCards and DisGeNET databases. (**C**) Intersection analysis of *M. longifolia* compound targets and depression-related genes, highlighting shared targets potentially implicated in antidepressant effects.


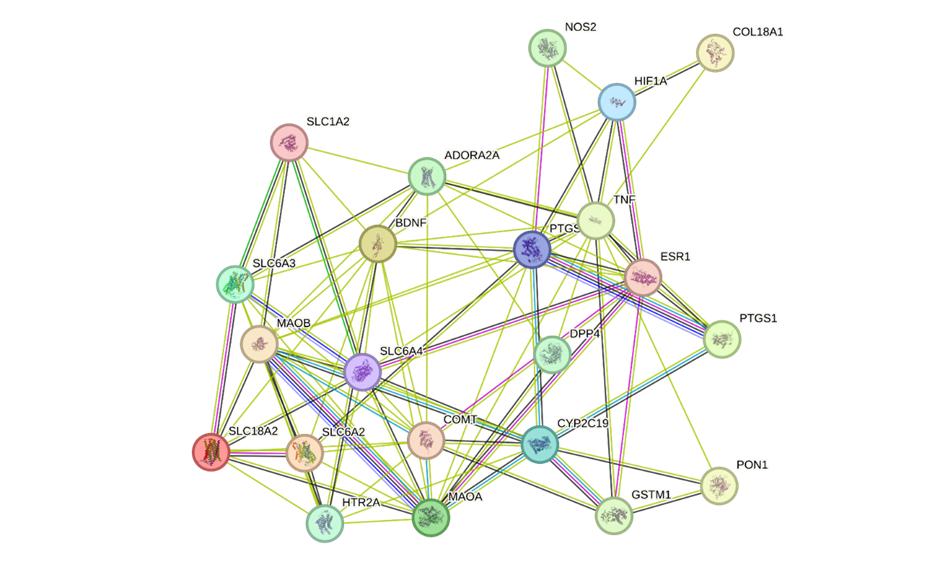


**Figure S5:** Protein–Protein Interaction (PPI) network formed between common genes of *Leonotis leonurus*.


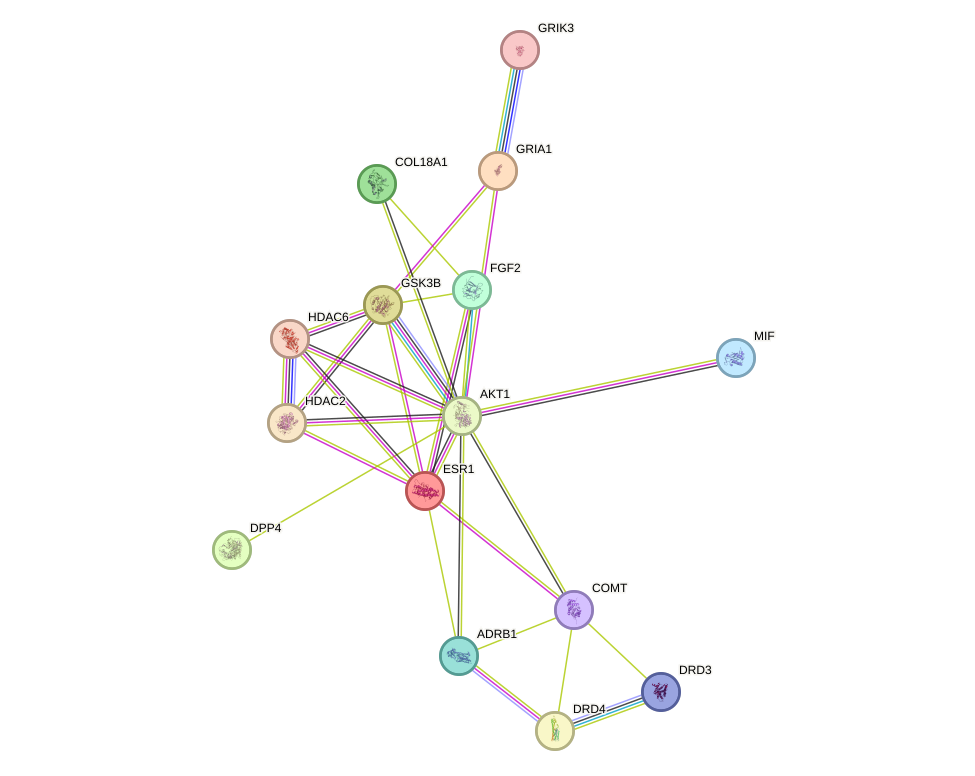


**Figure S6**: Protein–Protein Interaction (PPI) network formed between common genes of *Mentha longifolia.*


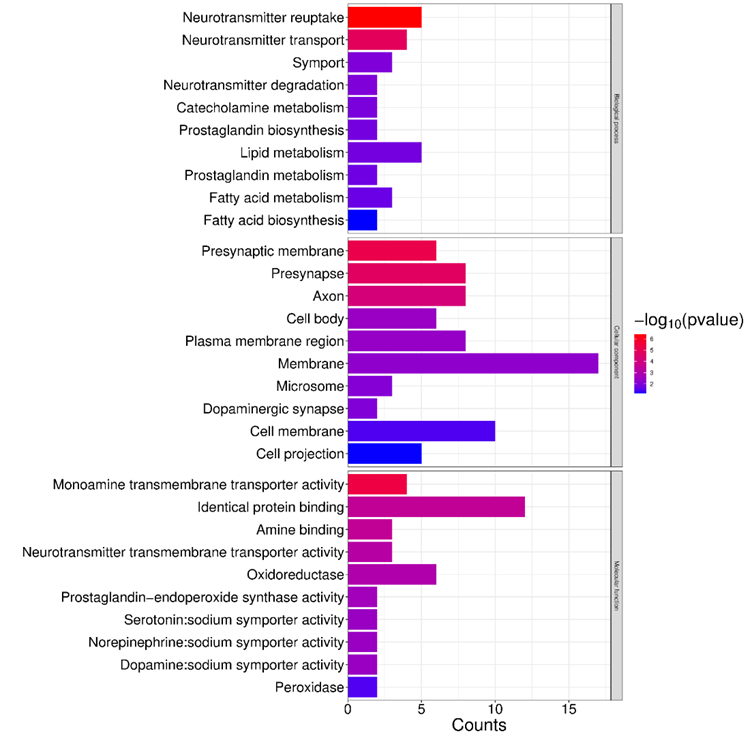


**Figure S7**: Gene Ontology (GO) analysis of the potential core targets of *Leonotis leonurus*. The enrichment colour bar plot of the top 10 biological processes (BP), cellular components (CC), and molecular function analysis of GO enrichment. GO terms are displayed on the y-axis, while enrichment significance and gene association patterns are represented by color intensity. The color scale reflects the statistical significance of enrichment based on adjusted p-values (false discovery rate, FDR), where darker or more intense colors indicate higher statistical significance (lower FDR values). Gene Ontology enrichment was performed using a hypergeometric test, and p-values were corrected for multiple testing using the Benjamini–Hochberg method. The enrichment factor represents the ratio of observed gene counts to expected gene counts for each GO term relative to the background reference set. Only GO terms with FDR < 0.05 were considered statistically significant.


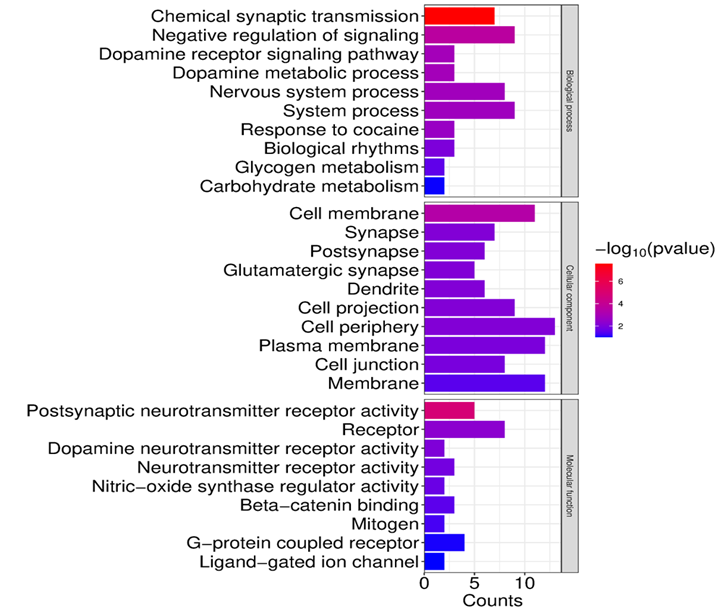


**Figure S8**: Gene Ontology (GO) analysis of the potential core targets of *Mentha longifolia*. The enrichment colour bar plot of the top 10 biological processes (BP), cellular components (CC), and molecular functions analysis of GO enrichment. GO terms are displayed on the y-axis, while enrichment significance and gene association patterns are represented by color intensity. The color scale reflects the statistical significance of enrichment based on adjusted p-values (false discovery rate, FDR), where darker or more intense colors indicate higher statistical significance (lower FDR values). Gene Ontology enrichment was performed using a hypergeometric test, and p-values were corrected for multiple testing using the Benjamini–Hochberg method. The enrichment factor represents the ratio of observed gene counts to expected gene counts for each GO term relative to the background reference set. Only GO terms with FDR < 0.05 were considered statistically significant.


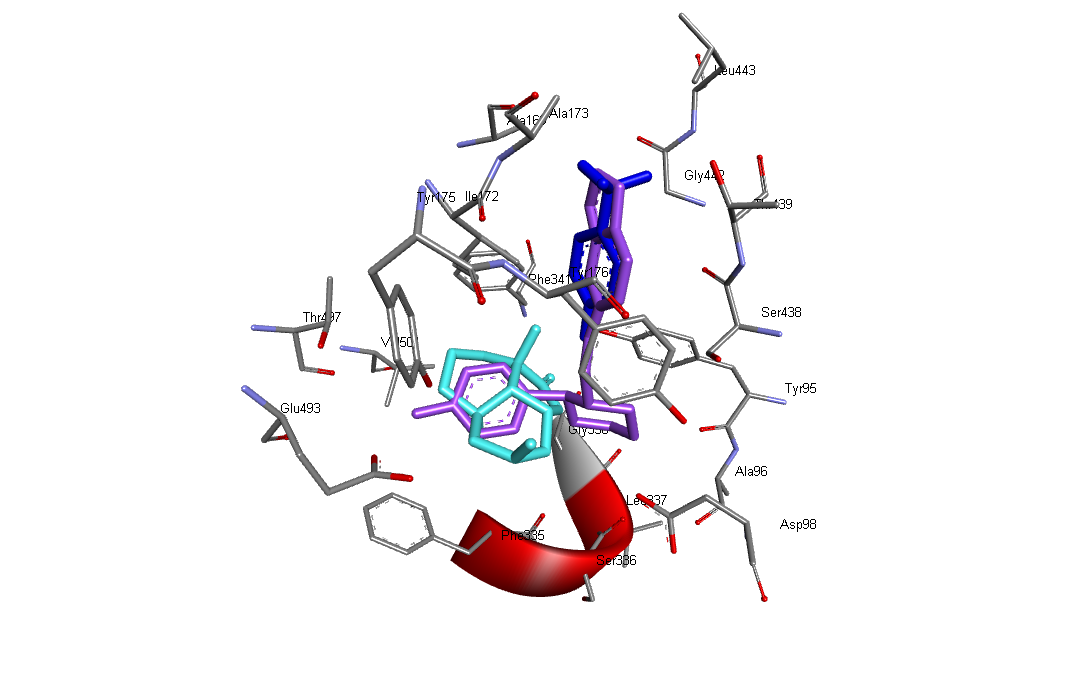


**Figure S9:** Docking validation of SLC_6_A_4_ demonstrating superimposition of the redocked co-crystallized ligand coloured in purple (standard, yellow) and the top three phytochemical ligands (green) within the active site pocket.


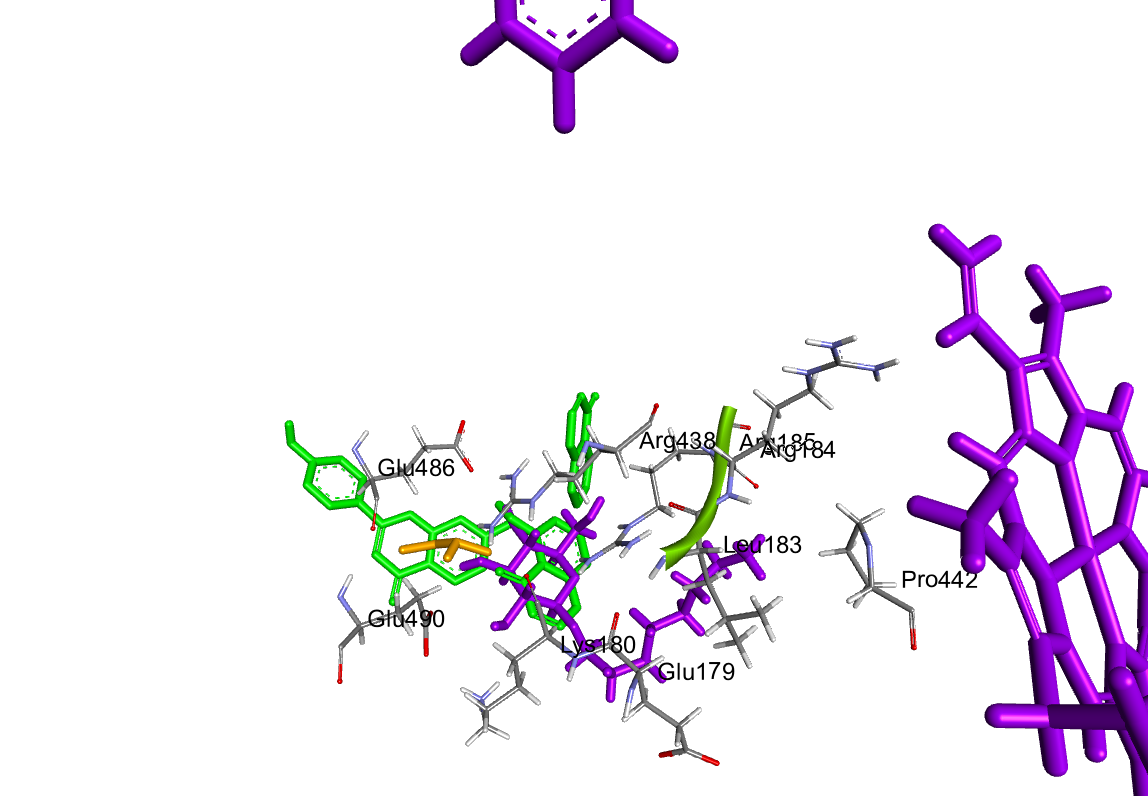


**Figure S10:** Docking validation of PTSG_2_ demonstrating superimposition of the redocked co-crystallized ligand coloured in purple (standard, yellow) and the top three phytochemical ligands (green) within the active site pocket.


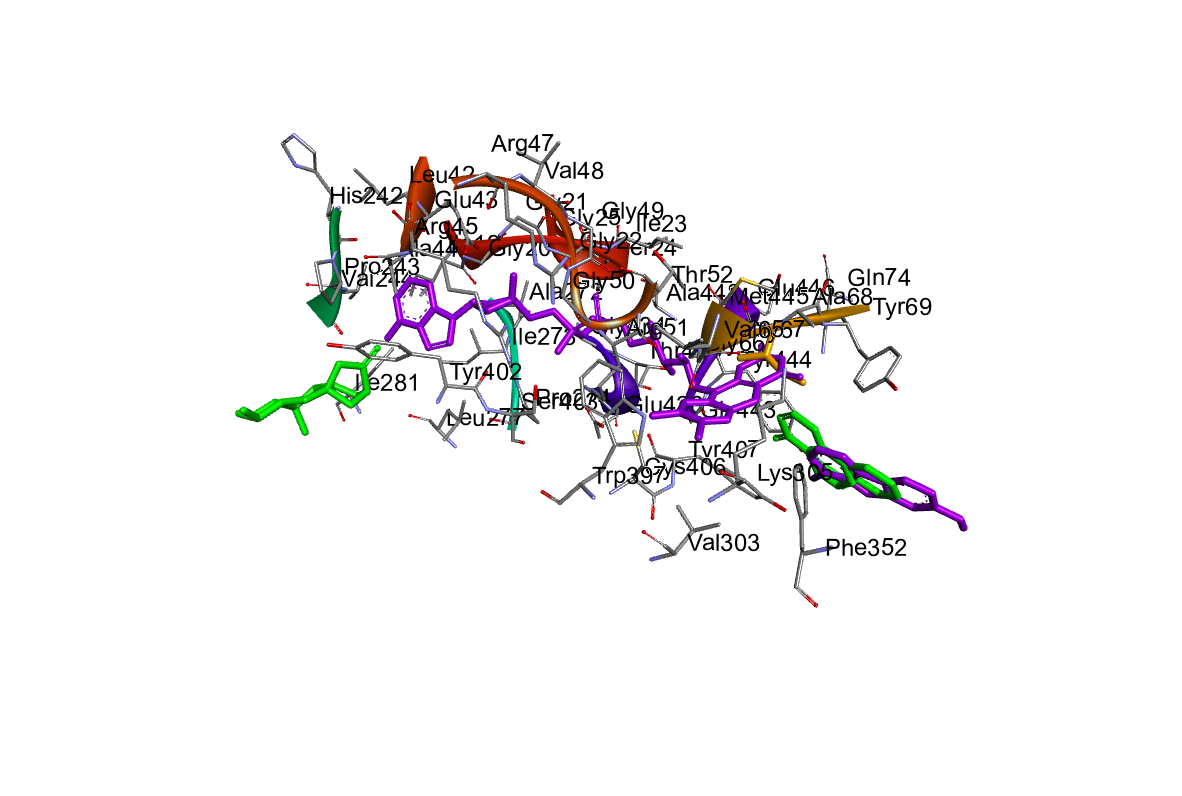


**Figure S11:** Docking validation of MAOA demonstrating superimposition of the redocked co-crystallized ligand coloured in purple (standard, yellow) and the top three phytochemical ligands (green) within the active site pocket.


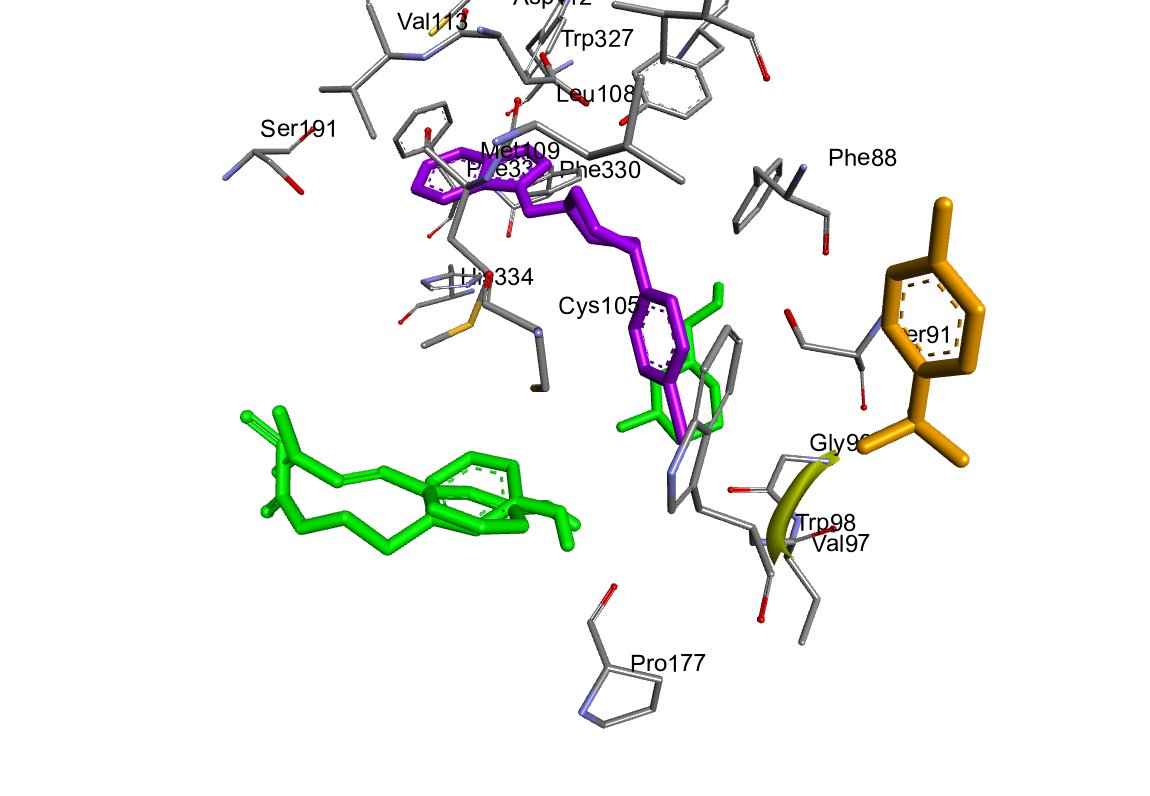


**Figure S12:** Docking validation of DRD_4_ demonstrating superimposition of the redocked co-crystallized ligand coloured in purple (standard, yellow) and the top three phytochemical ligands (green) within the active site pocket**.**


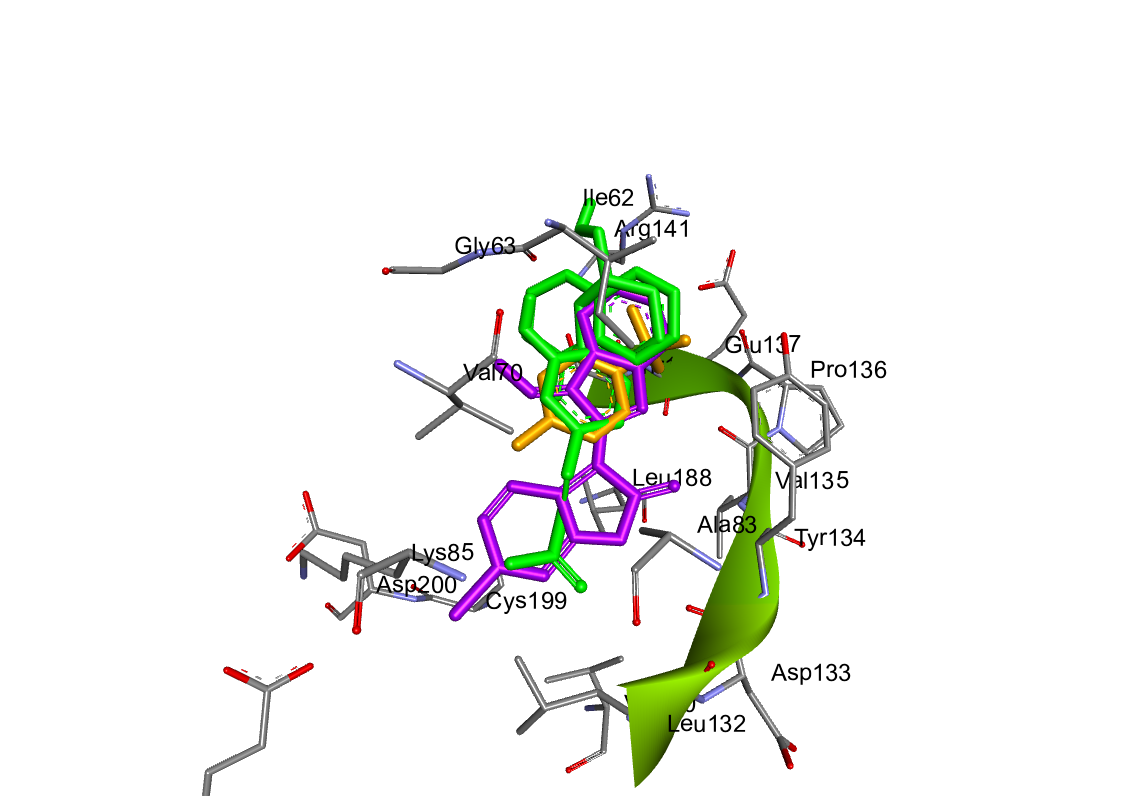


**Figure S13:** Docking validation of GSK_3_β demonstrating superimposition of the redocked co-crystallized ligand coloured in purple (standard, yellow) and the top three phytochemical ligands (green) within the active site pocket.


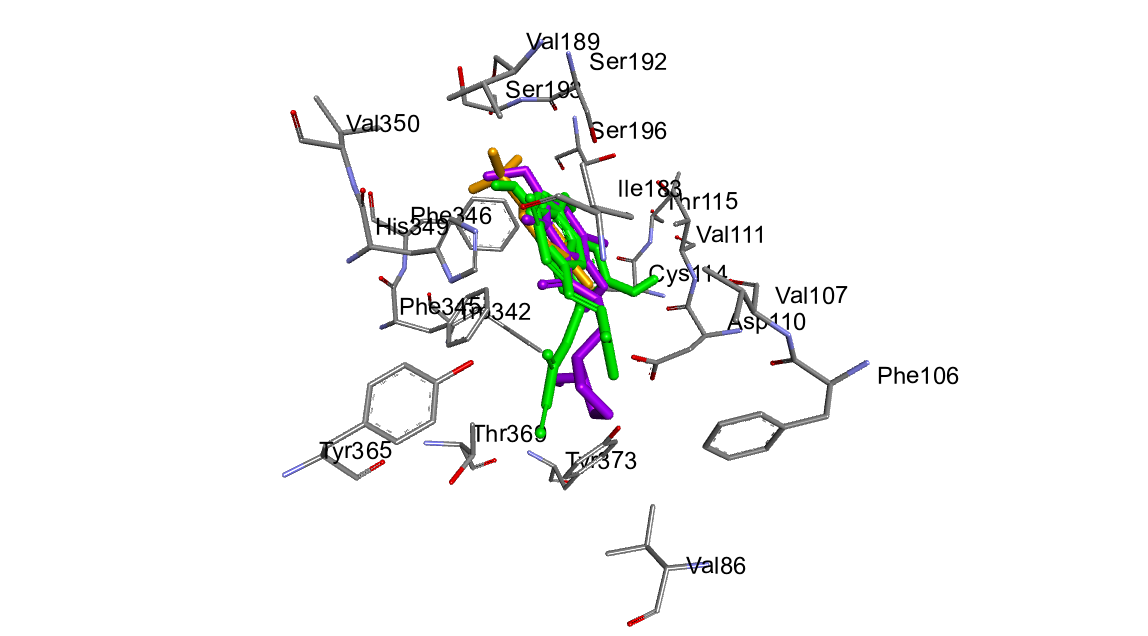


**Figure S14:** Docking validation of DRD_3_ demonstrating superimposition of the redocked co-crystallized ligand coloured in purple (standard, yellow) and the top three phytochemical ligands (green) within the active site pocket.


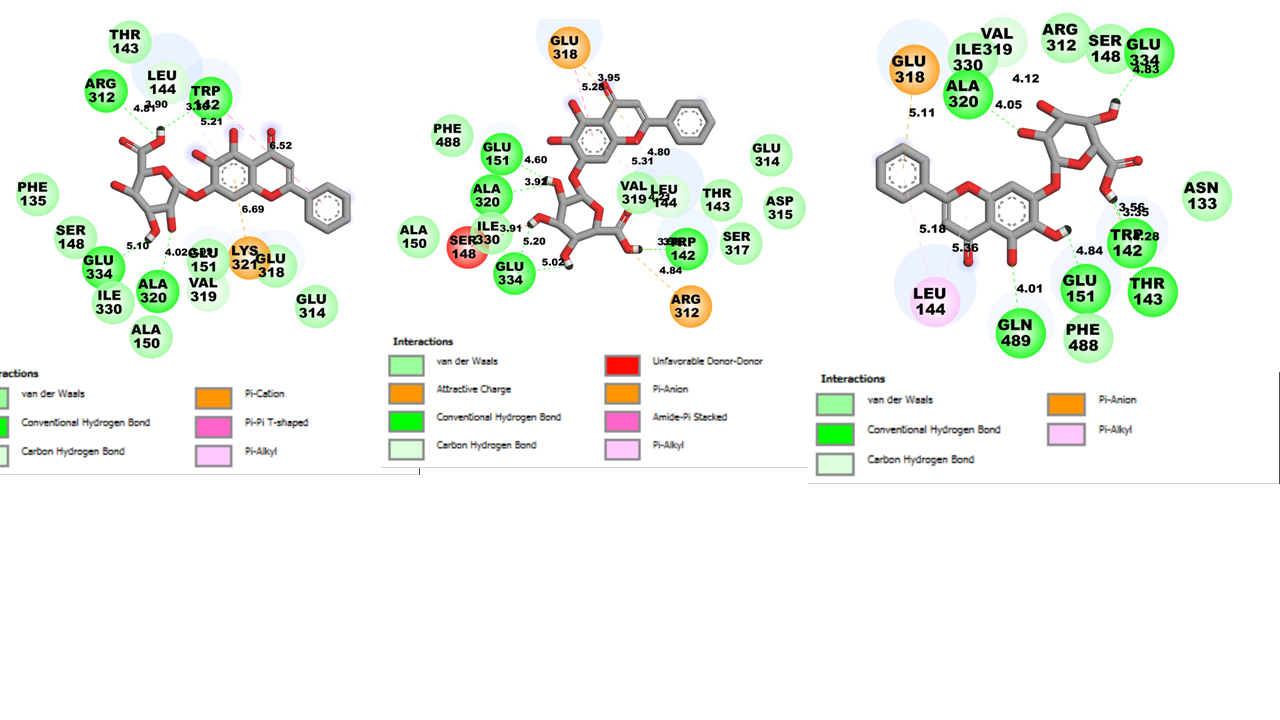
 **Figure S15**: Snapshot for Baicalin_*SLC_6_A_4_* at 50 ns, 100 ns, and 150 ns


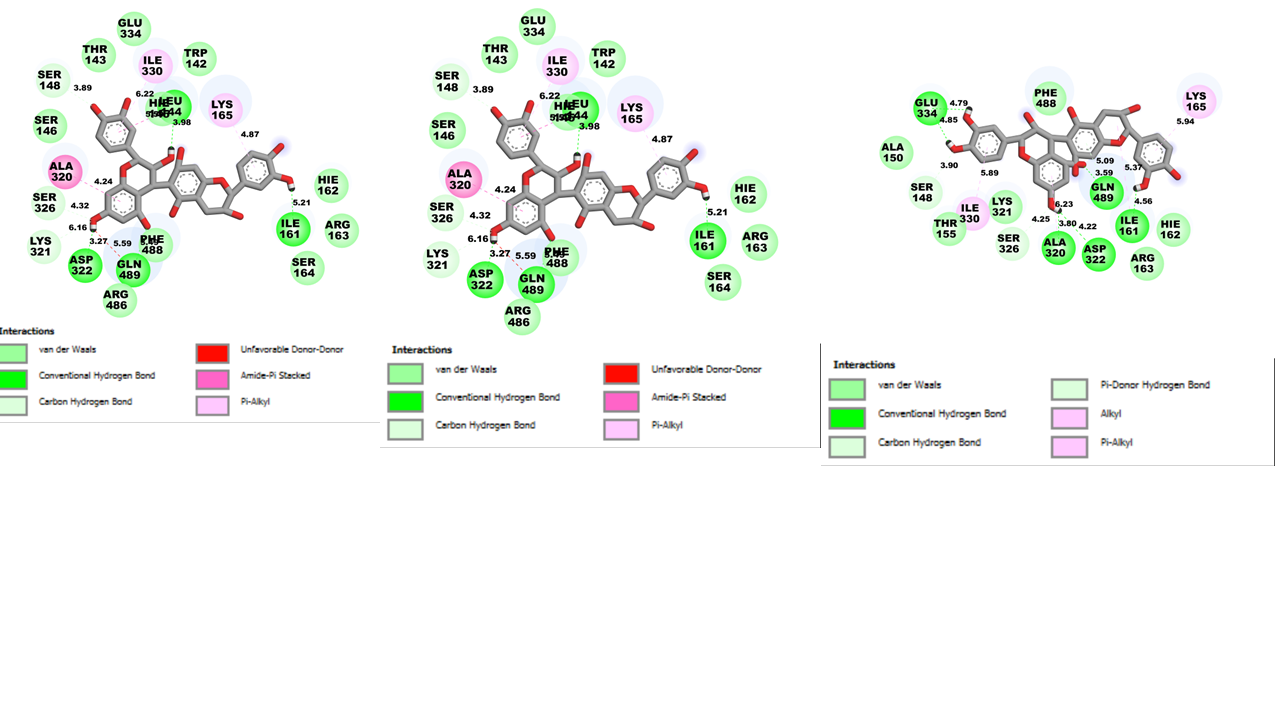


**Figure S16**: Snapshot for Procyanidin B5_*SLC_6_A_4_* at 50 ns, 100 ns, and 150 ns


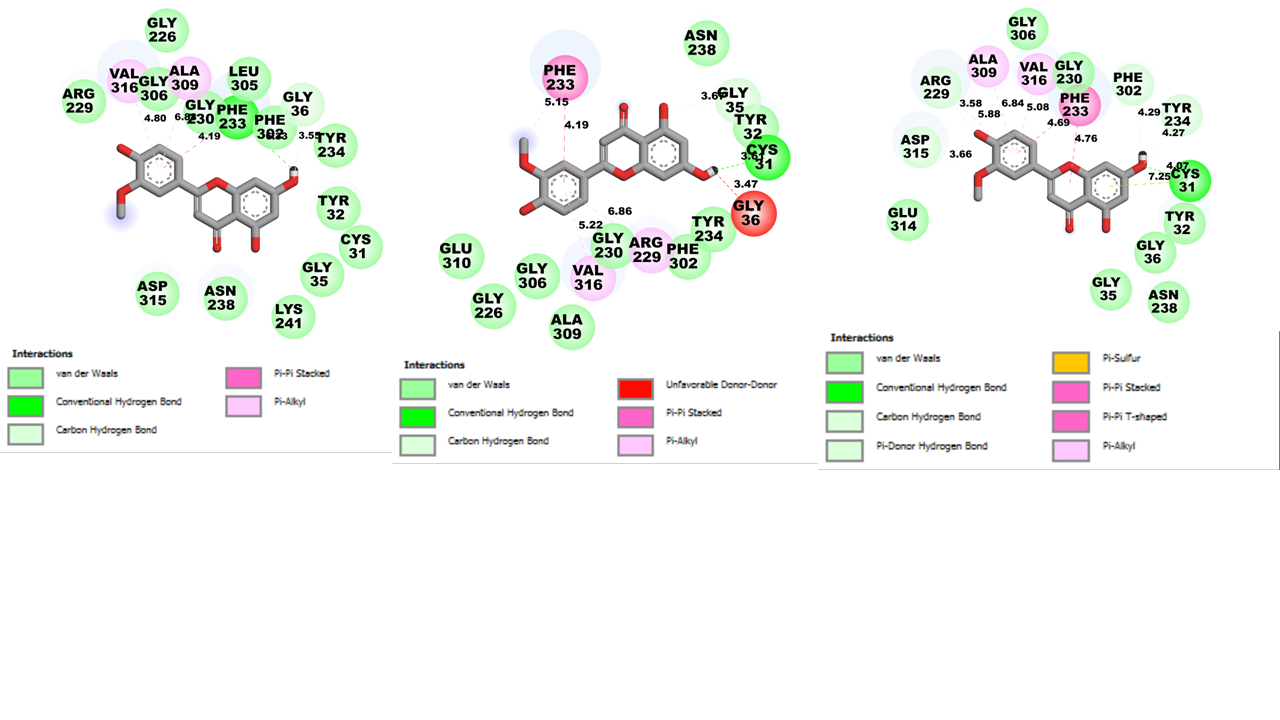


**Figure S17**: Snapshot for Chrysoeriol_*SLC_6_A_4_* at 50 ns, 100 ns, and 150 ns


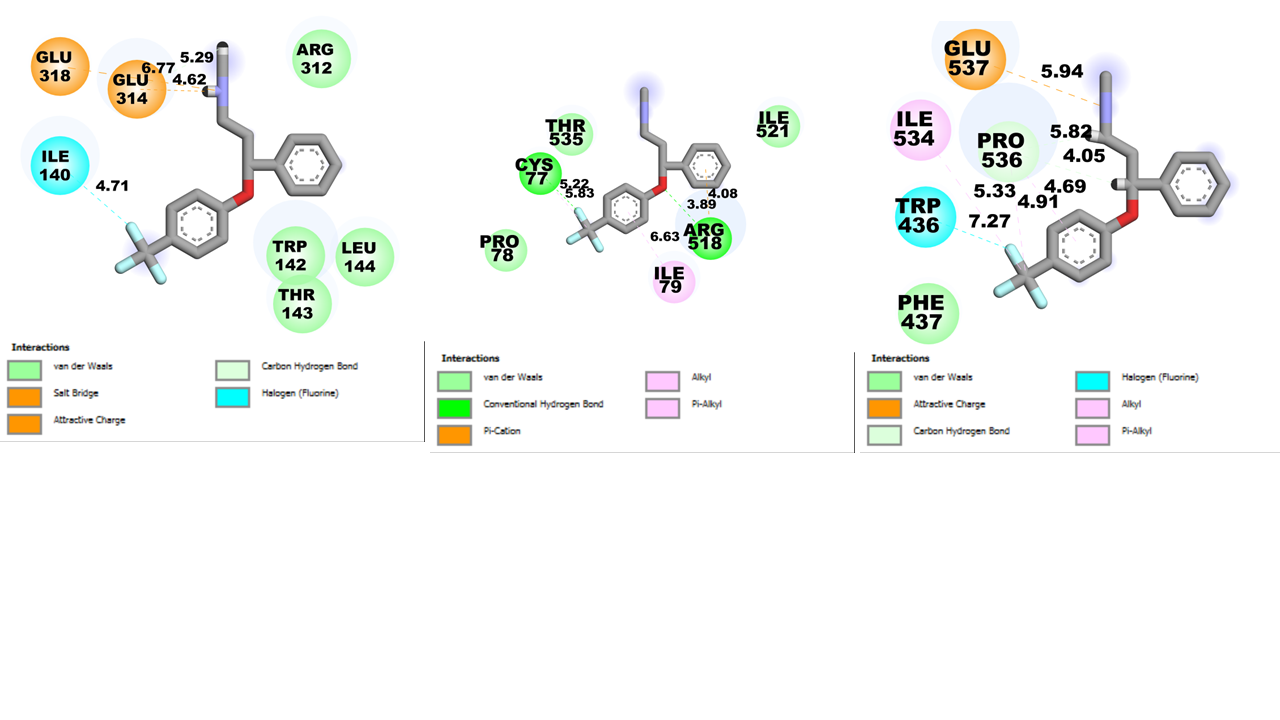


**Figure S18**: Snapshot for Fluoxetine_ *SLC_6_A_4_* at 50 ns, 100 ns, and 150 ns


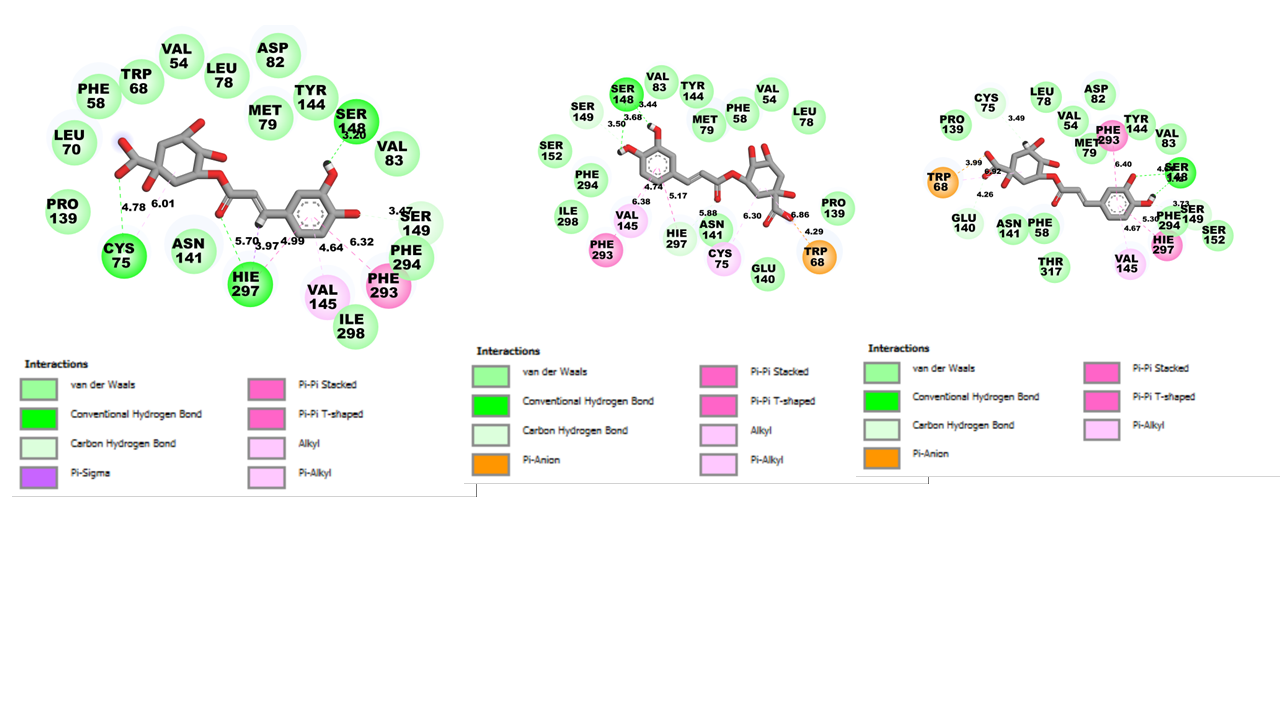


**Figure S19**: Snapshot for Chlorogenic acid_*DRD_4_* at 50 ns, 100 ns, and 150 ns


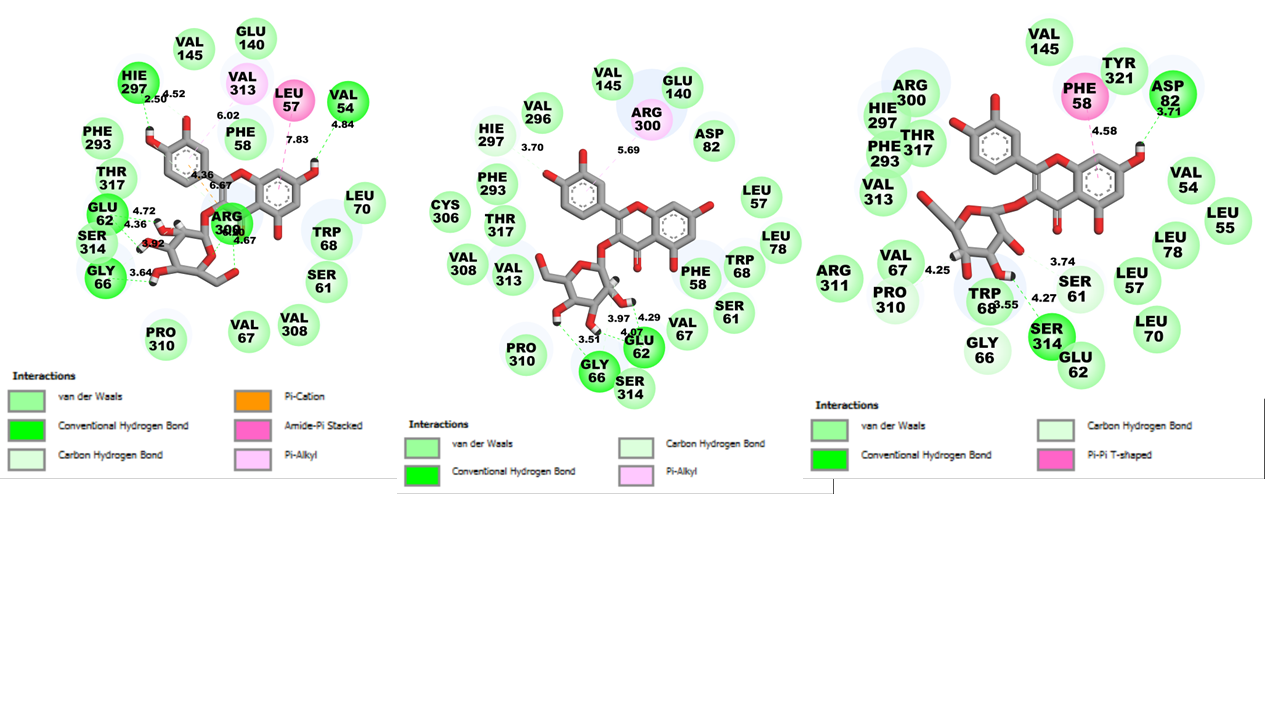
 **Figure S20**: Snapshot for Quercetin 3-galactoside_*DRD_4_* at 50 ns, 100 ns, and 150 ns


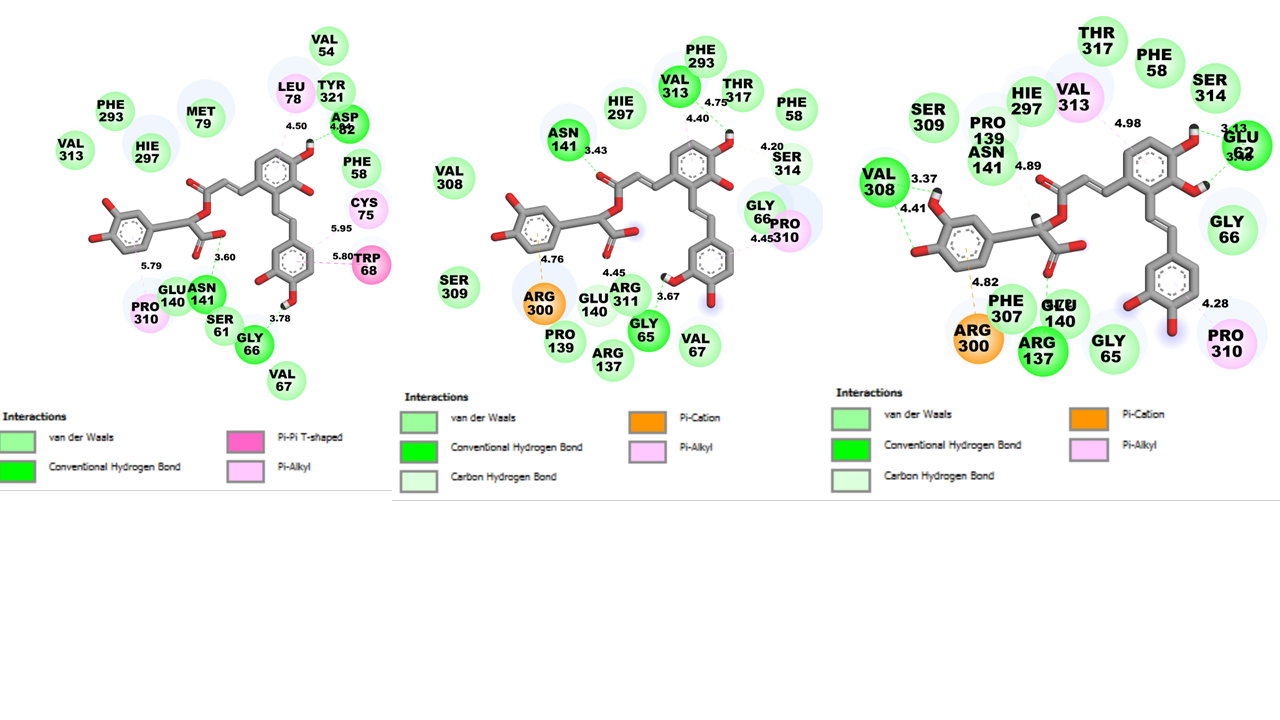


**Figure 21**: Snapshot for Salvianolic acid A_*DRD_4_* at 50 ns, 100 ns, and 150 ns


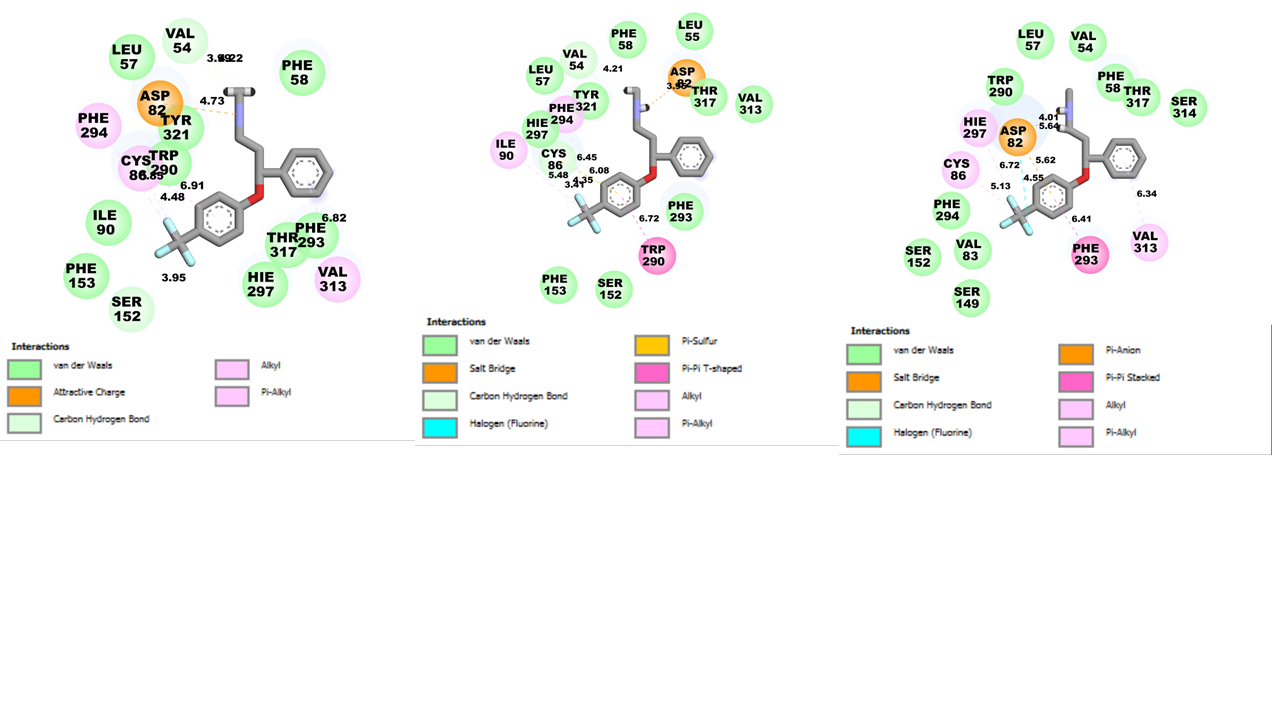


**Figure S22**: Snapshot for Fluoxetine_*DRD_4_* at 50 ns, 100 ns, and 150 ns

**Table S1**: The top 20 KEGG enrichment analysis results of *Leonotis leonurus*

| **Term description** | **Strength** | ***p*-value** | **Observed gene count** |
| --- | --- | --- | --- |
| 1. Serotonergic synapse | 1.82 | 1.13e-10 | 8 |
| 1. Cocaine addiction | 1.96 | 5.44e-07 | 5 |
| 1. Alcoholism | 1.57 | 1.43e-06 | 6 |
| 1. Synaptic vesicle cycle | 1.79 | 1.67e-06 | 5 |
| 1. Dopaminergic synapse | 1.55 | 1.94e-05 | 5 |
| 1. Drug metabolism - cytochrome P450 | 1.75 | 5.05e-05 | 4 |
| 1. Amphetamine addiction | 1.74 | 5.05e-05 | 4 |
| 1. Parkinson disease | 1.28 | 0.00024 | 5 |
| 1. Tyrosine metabolism | 1.89 | 0.00037 | 3 |
| 1. Arginine and proline metabolism | 1.76 | 0.00077 | 3 |
| 1. Arachidonic acid metabolism | 1.65 | 0.0014 | 3 |
| 1. Leishmaniasis | 1.59 | 0.0019 | 3 |
| 1. Chemical carcinogenesis | 1.55 | 0.0023 | 3 |
| 1. Metabolic pathways | 0.7 | 0.0024 | 8 |
| 1. Phenylalanine metabolism | 2.02 | 0.0045 | 2 |
| 1. Pathways in cancer | 0.94 | 0.0048 | 5 |
| 1. Histidine metabolism | 1.93 | 0.0059 | 2 |
| 1. Glycine | 1.67 | 0.0169 | 2 |
| 1. Tryptophan metabolism | 0.72 | 0.0185 | 2 |
| 1. Calcium signaling pathway | 0.6 | 0.0211 | 3 |

**Table S2**: The top 20 KEGG enrichment analysis results of *Mentha longifolia*

| **Term description** | **Strength** | ***p*-value** | **Observed gene count** |
| --- | --- | --- | --- |
| 1. Dopaminergic synapse | 1.8 | 1.29e-07 | 6 |
| 1. Thyroid hormone signaling pathway | 1.64 | 0.00032 | 4 |
| 1. Neuroactive ligand-receptor interaction | 1.3 | 0.00040 | 5 |
| 1. Breast cancer | 1.56 | 0.00040 | 4 |
| 1. Prolactin signaling pathway | 1.76 | 0.0013 | 3 |
| 1. EGFR tyrosine kinase inhibitor resistance | 1.71 | 0.0016 | 3 |
| 1. Pathways in cancer | 1.11 | 0.0016 | 5 |
| 1. Signaling pathways regulating pluripotency of stem cells | 1.45 | 0.0068 | 3 |
| 1. Gastric cancer | 1.43 | 0.0068 | 3 |
| 1. Tyrosine metabolism | 1.88 | 0.0119 | 2 |
| 1. Kaposi sarcoma-associated herpesvirus infection | 1.32 | 0.0119 | 3 |
| 1. Proteoglycans in cancer | 1.31 | 0.0119 | 3 |
| 1. cAMP signaling pathway | 1.28 | 0.0127 | 3 |
| 1. Regulation of lipolysis in adipocytes | 1.69 | 0.0195 | 2 |
| 1. Endometrial cancer | 1.66 | 0.0209 | 2 |
| 1. Longevity regulating pathway - multiple species | 1.63 | 0.0216 | 2 |
| 1. Amphetamine addiction | 1.61 | 0.0229 | 2 |
| 1. Melanoma | 1.56 | 0.0264 | 2 |
| 1. Chronic myeloid leukemia | 1.54 | 0.0271 | 2 |
| 1. B cell receptor signaling pathway | 1.53 | 0.0277 | 2 |

**Table S3**: Enrichment Analysis Results (GO) of *Leonotis leonurus*

| **Term** | **Count** | **P-Value** | **Class** |
| --- | --- | --- | --- |
| 1. Neurotransmitter transport | 4 | 1.4E-5 | Biological process |
| 1. Symport | 3 | 1.0E-2 | Biological process |
| 1. Neurotransmitter degradation | 2 | 1.0E-2 | Biological process |
| 1. Catecholamine metabolism | 2 | 1.2E-2 | Biological process |
| 1. Lipid metabolism | 5 | 1.4E-2 | Biological process |
| 1. Prostaglandin biosynthesis | 2 | 1.4E-2 | Biological process |
| 1. Prostaglandin metabolism | 2 | 1.7E-2 | Biological process |
| 1. Fatty acid metabolism | 3 | 1.9E-2 | Biological process |
| 1. Fatty acid biosynthesis | 2 | 6.7E-2 | Biological process |
| 1. Neurotransmitter reuptake | 5 | 4.02e-07 | Biological process |
| 1. Membrane | 17 | 5.3E-3 | Cellular component |
| 1. Microsome | 3 | 8.3E-3 | Cellular component |
| 1. Cell membrane | 10 | 3.4E-2 | Cellular component |
| 1. Cell projection | 5 | 6.6E-2 | Cellular component |
| 1. Presynaptic membrane | 6 | 6.55e-06 | Cellular component |
| 1. Presynapse | 8 | 1.81e-05 | Cellular component |
| 1. Axon | 8 | 6.44e-05 | Cellular component |
| 1. Cell body | 6 | 0.0036 | Cellular component |
| 1. Plasma membrane region | 8 | 0.0040 | Cellular component |
| 1. Dopaminergic synapse | 2 | 0.0094 | Cellular component |

**Table S4**: Enrichment Analysis Results (GO) of *Mentha longifolia*

| **Term** | **Count** | **P-Value** | **Class** |
| --- | --- | --- | --- |
| Biological rhythms | 3 | 1.0E-2 | Biological process |
| Glycogen metabolism | 2 | 2.7E-2 | Biological process |
| Carbohydrate metabolism | 2 | 9.4E-2 | Biological process |
| Chemical synaptic transmission | 7 | 2.67e-08 | Biological process |
| Dopamine receptor signalling pathway | 3 | 0.0012 | Biological process |
| Negative regulation of signalling | 9 | 0.00023 | Biological process |
| Dopamine metabolic process | 3 | 0.0012 | Biological process |
| Nervous system process | 8 | 0.0015 | Biological process |
| System process | 9 | 0.0017 | Biological process |
| Response to cocaine | 3 | 0.0024 | Biological process |
| Cell membrane | 11 | 3.7E-4 | Cellular component |
| Membrane | 12 | 3.2E-2 | Cellular component |
| Dendrite | 6 | 0.0078 | Cellular component |
| Cell projection | 9 | 0.0078 | Cellular component |
| Synapse | 7 | 0.0078 | Cellular component |
| Cell periphery | 13 | 0.0078 | Cellular component |
| Postsynapse | 6 | 0.0078 | Cellular component |
| Glutamatergic synapse | 5 | 0.0078 | Cellular component |
| Plasma membrane | 12 | 0.0105 | Cellular component |
| Cell junction | 8 | 0.0117 | Cellular component |
| Receptor | 8 | 5.0E-3 | Molecular function |
| Mitogen | 2 | 5.0E-2 | Molecular function |
| G-protein coupled receptor | 4 | 8.8E-2 | Molecular function |
| Ligand-gated ion channel | 2 | 9.7E-2 | Molecular function |
| Postsynaptic neurotransmitter receptor activity | 5 | 1.39e-05 | Molecular function |
| Dopamine neurotransmitter receptor activity | 2 | 0.0089 | Molecular function |
| Neurotransmitter receptor activity | 3 | 0.0142 | Molecular function |
| Nitric-oxide synthase regulator activity | 2 | 0.0199 | Molecular function |
| Beta-catenin binding | 3 | 0.0303 | Molecular function |

**Table S5**: Integrated summary of top phytochemical-target-pathway interactions and computational outcomes.

| **Compound** | **Predicted target** | **Pathway** | **Docking score (kcal/mol)** | **MD outcome** | **MMGBSA (kcal/mol)** | **Key residues** | **Biological Interpretation** |
| --- | --- | --- | --- | --- | --- | --- | --- |
| Procyanidin B5 | *SLC_6_A_4_* | Serotonergic synapse | -9.5 |  |  |  | Potential serotonergic modulation linked to antidepressant activity. |
| Baicalin | *PTSG_2_* | Neuroinflammation pathway | -6.5 |  |  |  | May contribute to anti-inflammatory effects associated with depression |
| Chrysoeriol | *MAOA* | Monoaminergic signaling | -9.5 |  |  |  | Potential regulation of monoamine metabolism. |
| Salvianolic acid A | *DRD_4_* | Dopaminergic signaling pathway | -8.4 |  |  |  | Potential modulation of dopaminergic signaling associated with antidepressant-related mechanisms. |
| Rosmarinic acid | *GSK_3_β* | PI_3_K-Akt / Neuroplasticity-related signaling | -8.7 |  |  |  | May contribute to neuroprotective and antidepressant-related mechanisms through GSK3β modulation. |
| Salvianolic acid A | *DRD_3_* | Dopaminergic signaling pathway | -9.5 |  |  |  | Potential involvement in dopaminergic neurotransmission linked to antidepressant activity |
